# Circulating Myokine Responses to Acute Endurance Exercise and Their Role in Immunoregulation: A Systematic Review and Meta‐Analysis

**DOI:** 10.1096/fj.202504780R

**Published:** 2026-02-09

**Authors:** Miriam Ringleb, Fabian Fabritius, Jakob Godde, Christian Puta, Wilhelm Bloch, Florian Javelle

**Affiliations:** ^1^ NeuroPyschoImmunology Research Unit, Department for Molecular and Cellular Sports Medicine, Institute of Cardiovascular Research and Sports Medicine German Sport University Cologne Cologne Germany; ^2^ Center for Interdisciplinary Prevention of Diseases Related to Professional Activities Friedrich‐Schiller‐University Jena Jena Germany; ^3^ Department of Sports Medicine and Health Promotion Friedrich‐Schiller‐University Jena Jena Germany; ^4^ Center for Sepsis Control and Care (CSCC), Jena University Hospital Friedrich‐Schiller‐University Jena Jena Germany; ^5^ Department for Molecular and Cellular Sports Medicine, Institute of Cardiovascular Research and Sports Medicine German Sport University Cologne Cologne Germany

**Keywords:** acute effects, endurance exercise, exerkines, immune system, inflammation, myokines

## Abstract

Acute endurance exercise influences metabolic and immune functions, with myokines playing a key role in mediating communication between these two systems and in transforming transient inflammatory signals into long‐term anti‐inflammatory adaptations. However, the magnitude of these anti‐inflammatory effects varies widely depending on exercise type, intensity, and duration. Accordingly, the objective of this meta‐analysis is to assess changes in myokine concentrations following endurance exercise and establish a potential dose–response relationship between exercise‐related parameters. A systematic literature search was conducted in June 2024 for endurance exercise studies measuring changes in interleukin (IL‐) 6, IL‐10, IL‐1ra, tumor necrosis factor (TNF‐) α, IL‐15, IL‐7, IL‐8, transforming growth factor β1, and fractalkines before and immediately after exercise in healthy individuals. Independent random‐effect meta‐analyses were performed for each myokine (PROSPERO: CRD42024535364). Significant small‐moderate to very large positive effect sizes were observed for IL‐6, IL‐10, IL‐1ra, IL‐8, IL‐15, and TNF‐α. These effects were significantly moderated by risk of bias, sex, age, V̇O_2_peak, experience, exercise type, intensity, duration, dose, sample, fasting state, and time of day. This meta‐analysis demonstrates that myokine responses following endurance exercise are clearly increased and moderated by factors such as exercise duration, intensity, exercise type, and training status, allowing their targeted modulation for therapeutic use. Even “suboptimal exercise conditions” can elicit significant increases in immunoregulatory myokines, thereby promoting beneficial immune responses, suggesting that endurance exercise is a key component in the treatment of a wide range of diseases.

## Graphical abstract

This systematic review and meta‐analysis, which examined the effects of acute endurance training on immunoregulatory myokines, showed significant small‐moderate to very large positive effect sizes for IL‐6, IL‐10, IL‐1ra, IL‐8, IL‐15, and TNF‐α. These effects were significantly moderated by risk of bias, sex, age, V̇O_2_peak, experience, type of exercise, intensity, duration, dose, sample, fasting status, and time of day. This suggests that endurance training is a key component in the treatment of a variety of diseases.

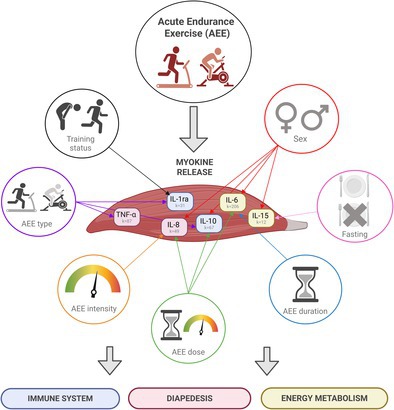

**Abbreviations**
AMP: Adenosine‐monophosphate
AMPK: AMP‐activated protein kinase
ATP: Adenosine‐triphosphate
BMI: Body mass index
CD: Cluster of differentiation
ELISA: Enzyme‐linked immunosorbent assay
FKN: Fractalkines
*g*
: Hedges' *g*
HIIT: High‐intensity interval training
ICAM‐1: Intercellular adhesion molecule‐1
IL: Interleukin
MCT: Moderate continuous training
MET: Metabolic equivalent
NAD^+^
: Nicotinamide adenine dinucleotide
NK cell: Natural killer cell
PGC‐1α: Peroxisome proliferator‐activated receptor‐γ coactivator 1α
PI: Prediction interval
SD: Standard deviation
SIRT1: Sirtuin 1
SMD: Standardized mean differences
TGF: Transforming growth factor
TNF: Tumor necrosis factor
Treg: Regulatory T cell
VCAM‐1: Vascular cell adhesion molecule‐1
VO_2_peak: Peak oxygen consumption

## 1 Introduction

Physical exercise is widely recognized as a key modulator of metabolic and immune function, exerting systemic health benefits through both acute and chronic physiological adaptations. It decreases the risk of obesity, cardiovascular diseases, Type 2 diabetes mellitus, cognitive decline, and many cancers [1, 2]. Regular exercise reduces adipose tissue and prevents excessive inflammation by maintaining a balance between pro‐ and anti‐inflammatory immune responses [3, 4]. While long‐term exercise leads to sustained metabolic improvements and reduced inflammation [5], acute exercise triggers short‐term immune responses that help maintain metabolic homeostasis and support tissue repair. It is characterized by a transient inflammatory response, counterbalanced by coordinated anti‐inflammatory cellular and humoral mechanisms [6]. Exerkines, defined as signal molecules released in response to exercise [7], play a key role in creating this anti‐inflammatory environment induced by acute physical activity. In response to both acute and chronic physical exercise, exerkines are secreted and act through multiple signaling pathways [8]. Depending on their tissue of origin, exerkines can be cardiokines (heart), hepatokines (liver), adipokines (white adipose tissue), baptokines (brown adipose tissue), neurokines (brain), and myokines (skeletal muscle) [8].

Among these, myokines have been particularly well‐studied, with interleukin (IL‐) 6 being the most extensively investigated. First identified as a myokine in the early 2000s by Pedersen's group [9], IL‐6 was shown to have different effects when secreted during chronic inflammation or sepsis compared to when secreted transiently and locally following exercise from the working muscle [10]. While chronic inflammation is characterized by a sustained systemic increase in IL‐6 alongside other pro‐inflammatory signals, such as tumor necrosis factor‐alpha (TNF‐α) and immune cell activation, exercise‐induced IL‐6 peaks quickly, directly at the end of the exercise, without simultaneously rising more pro‐inflammatory messengers [11]. This rise in IL‐6 and further myokines leads to changes in the metabolic system and the immune response.

In the context of endurance exercise, characterized by a high metabolic demand [10], myokines, particularly exercise‐induced IL‐6, are secreted from skeletal muscle primarily in response to changing energy requirements. This process is partly triggered by the activation of AMP‐activated protein kinase (AMPK), the key regulator of cellular energy homeostasis. AMPK is activated by shifts in adenine nucleotide ratios, particularly a decrease in ATP and an increase in AMP [12]. The following phosphorylation of peroxisome proliferator‐activated receptor‐γ coactivator 1α (PGC‐1α) coactivates multiple transcription factors, leading to increased expression of IL‐6‐related genes [13, 14]. In parallel, this signaling pathway stimulates transcriptional programs involved in mitochondrial biogenesis, oxidative phosphorylation, fatty acid oxidation, gluconeogenesis, and thermogenesis [15]. The subsequent rise in exercise‐induced IL‐6 promotes glycogen breakdown, thereby increasing substrate availability for ATP synthesis to meet the elevated energy demands during exercise [16]. AMPK activation also increases intracellular nicotinamide adenine dinucleotide (NAD^+^) availability, in part by promoting mitochondrial oxidation of cytosolic reducing equivalents. This enhances the activity of sirtuin 1 (SIRT1), a NAD^+^‐dependent energy sensor [17, 18], which deacetylates PGC‐1α and further amplifies its transcriptional activity [17]. Collectively, this signaling cascade promotes the expression and secretion of IL‐6 [19], which in this context acts primarily as a metabolic regulator rather than a classical inflammatory marker.

Nonetheless, IL‐6 also acts as an immunoregulator by stimulating the production of anti‐inflammatory cytokines such as IL‐10 and IL‐1ra [20]. A central mediator of the acute anti‐inflammatory effects of exercise is the sympathetic nervous system, which is activated during acute exercise and characterized by increased levels of catecholamines, epinephrine, and norepinephrine [21]. This neuroendocrine response induces a transient mobilization of immune cells, particularly lymphocytes and natural killer (NK) cells, into the circulation [22]. In addition, the cytolytic activity of NK cells increases following acute exercise, independent of changes in their absolute numbers [23]. Together with contraction‐induced signaling in skeletal muscle, this immune cell mobilization contributes to the coordinated release of immunoregulatory exerkines. For example, transforming growth factor (TGF)‐β1 promotes regulatory T‐cell (Treg) expansion and suppresses the differentiation of pro‐inflammatory T helper cell subsets, such as Th1 cells [24]. IL‐10 and IL‐15 further enhance NK cell activity and numbers [25]. Moreover, IL‐10 also supports the differentiation of B cells and Tregs [26, 27], and inhibits the recruitment of monocytes and macrophages as well as the differentiation of proinflammatory T cells [28, 29]. Additional exerkines, including fractalkines (FKN), IL‐7, and IL‐8, contribute to immune cell activation, homeostasis, and recruitment [30, 31, 32].

**FIGURE 1:**
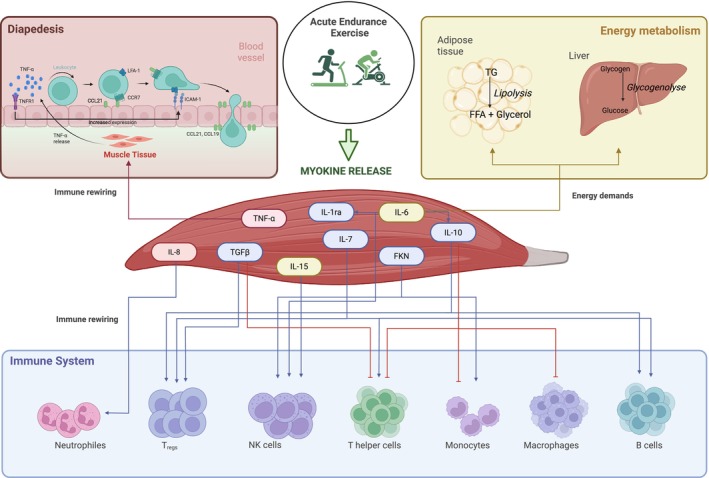
Myokine release in response to acute endurance exercise and its impact on immune system, diapedesis, and energy metabolism. Acute endurance exercise triggers the release of immunoregulatory myokines from the working skeletal muscle. Among these, IL‐6 plays a dual role: It acts as a key energy sensor by promoting lipolysis in adipose tissue and glycogenolysis in the liver, thereby increasing substrate availability to meet energy demands. In addition, IL‐6 stimulates the production of the anti‐inflammatory cytokines IL‐10 and IL‐1ra, the latter of which blocks the IL‐1 receptor to further dampen inflammation. TNF‐α promotes the transmigration of immune cells into the muscle tissue, known as diapedesis. It activates the vascular endothelium by increasing the expression of adhesion molecules such as ICAM‐1, which enable leukocytes to adhere to the vessel walls. Additionally, several myokines directly influence immune cell differentiation and muscle adaptation. TGF‐β1 also modulates immune responses by promoting Treg expansion and inhibiting the differentiation of pro‐inflammatory Th1 cells. IL‐10 and IL‐15 enhance the activity and number of NK cells, while IL‐10 additionally supports the differentiation of B cells and Tregs, and inhibits the recruitment of monocytes and macrophages, as well as the differentiation of pro‐inflammatory T cells. FKN further supports the activation of monocytes and NK cells, whereas IL‐7 ensures T‐cell homeostasis, and IL‐8 boosts the recruitment and activity of granulocytes. CCL, CC Motif Chemokine Ligand; CCR, CC motif chemokine receptor; FFA, free fatty acid; FKN, fractalkine; ICAM‐1, intercellular adhesion molecule 1; IL, interleukin; LFA‐1, lymphocyte function‐associated antigen 1; NK cell, natural killer cell; ra, receptor antagonist; TG, triglycerides; TGF‐β1, Transforming growth factor β1; TNFR1, Tumor necrosis factor receptor 1; TNF‐α, Tumor necrosis factor α; Tregs, regulatory T cell. Created with biorender.com.

Collectively, these responses illustrate how acute exercise induces a tightly regulated, transient immunoregulatory environment that integrates neural, muscular, and immune signals. Figure 1 provides a simplified overview of these exercise‐induced interactions between immunoregulatory myokines and other tissues and organs.

The primary aim of this analysis i2s to assess changes in myokine concentrations following endurance exercise and examine a potential dose–response relationship with exercise‐related parameters. In this context, the exercise *dose* can be defined as the combined influence of exercise intensity and duration [33] on post‐exercise myokine responses. Establishing such a dose–response relationship may enable the development of more tailored exercise prescriptions, particularly for populations with specific needs, including individuals with metabolic disorders, autoimmune diseases, or psychiatric conditions. Numerous studies have examined the acute effects of exercise on circulating concentrations of immunoregulatory myokines and on skeletal muscle mRNA expression. However, findings across the literature remain inconsistent with respect to the effect magnitude. Furthermore, while we demonstrated in a previous meta‐analysis that acute resistance exercise elicits moderate effects on IL‐6 and IL‐1ra immediately post‐exercise, and no significant effect on IL‐10 [33], a large share of studies investigating acute endurance exercise report much stronger responses [34, 35, 36]. Nevertheless, so far, no review or meta‐analysis has quantified the effects of acute endurance exercise on immunoregulatory myokines and investigated possible moderators of these effects.

Therefore, this meta‐analysis examines the acute response of circulating immunoregulatory myokines following a single endurance exercise session. The immediate post‐exercise time point was selected as it reflects the peak of metabolic and physiological stress, during which transient but functionally relevant changes in myokine concentrations are most pronounced. The included myokines (IL‐6, IL‐10, IL‐1ra, TNF‐α, IL‐15, IL‐7, IL‐8, TGF‐β1, and FKN) were selected based on their established or proposed roles in immunomodulation, responsiveness to acute exercise stimuli, and regulation by metabolic and cardiovascular stress.

## 2 Materials and Methods

The literature search and writing process were performed according to the Preferred Reporting Items for Systematic Reviews and Meta‐Analyses (PRISMA) 2020 guidelines [37]. The PRISMA checklist is provided in the Appendix S1. The protocol was pre‐registered on PROSPERO (registration number: CRD42024535364, last amendment date: 25/06/24). Independent meta‐analyses were conducted for IL‐6, IL‐10, IL‐1ra, IL‐8, IL‐15, and TNF‐α. All data, computation files, and scripts are openly available on Open Science Framework (https://doi.org/10.17605/OSF.IO/T6ZND).

### 2.1 Literature Search

The databases MEDLINE (via PubMed), Web of Science, Cochrane Library, and SPORTDiscus were systematically searched on June 13th, 2024. The search query was created based on MeSH terms and related vocabulary covering the main domains of endurance exercise and interleukins (Table 1). In addition, the reference lists of the included studies were screened.

**TABLE 1:** Exemplified search query.

MEDLINE (via PubMed)
((endurance*[Title/abstract] OR aerobic*[Title/abstract] OR “interval training” [Title/abstract] OR cycling [Title/abstract] OR running [Title/abstract] OR marathon [Title/abstract] OR swimming [Title/abstract] OR rowing [Title/abstract]) AND (Interleukin‐6 [Title/abstract] OR Interleukine‐6 [Title/abstract] OR IL‐6 [Title/abstract] OR Interleukin‐6 [Mesh] OR “Interleukin 1 Receptor Antagonist Protein” [Title/abstract] OR “Interleukin 1 Receptor Antagonist” [Title/abstract] OR IL‐1ra [Title/abstract] OR “Interleukin 1 Receptor Antagonist Protein” [Mesh] OR IL‐8 [Title/abstract] OR Interleukin‐8 [Title/abstract] OR Interleukin‐10 [Title/abstract] OR IL‐10 [Title/abstract] OR cytokin* [Title/abstract] OR myokin* [Title/abstract] OR exerkin* [Title/abstract] OR Interleukin‐7 [Title/abstract] OR IL‐7 [Title/abstract] OR Interleukin‐15 [Title/abstract] OR IL‐15 [Title/abstract] OR Fractalkine [Title/abstract] OR CX3CL1 [Title/abstract] OR “tumor necrosis factor” [Title/abstract] OR “tumor necrosis factor” [Title/abstract] OR tumor‐necrosis‐factor [Title/abstract] OR TNF [Title/abstract] OR TGFβ1 [Title/abstract] OR “Transforming growth factor” [Title/abstract] OR TGF‐beta [Title/abstract])) NOT (infant [MeSH] OR child [MeSH] OR adolescent [MeSH] OR review [Publication Type] OR Systematic Review [Publication Type])

### 2.2 Eligibility Criteria

Eligibility criteria were defined using the PICOS framework (Population, Intervention, Comparison, Outcomes, Study design).

#### 2.2.1 Population

Only studies investigating healthy humans between 18 and 50 years were included. Studies involving individuals over 50 years or with any acute or chronic illness or injury were excluded to avoid confounding immune alterations.

#### 2.2.2 Interventions

Studies examining a single bout of endurance exercise (≥ 20 min, e.g., running or cycling) were included. If an acute pre–post measurement was embedded in a training program, only the acute session was considered. Studies combining endurance with resistance training, long‐term programs, or additional treatments were excluded, as were non‐endurance activities (e.g., yoga, stretching). For multiarm studies, only endurance‐specific interventions were included.

#### 2.2.3 Comparison

To be included, studies were required to measure outcome parameters both before exercise (on the same day) and immediately post‐exercise (within 5 min). Studies with no baseline measurement or follow‐up measurements later than 5 min post‐exercise were excluded from the review.

#### 2.2.4 Outcomes

Studies assessing changes in blood serum or plasma concentrations of IL‐6, IL‐10, IL‐1ra, TNF‐α, IL‐15, IL‐7, TGF‐β1, FKN, and IL‐8 via enzyme‐linked immunosorbent assay (ELISA) were included in the review. Other measurement techniques (e.g., flow cytometry) were excluded.

#### 2.2.5 Study Design

Randomized and non‐randomized clinical trials published in English, French, or German in a peer‐reviewed journal were included in this review. Case studies, animal studies, reviews, cross‐sectional or retrospective studies, and longitudinal study designs were excluded.

### 2.3 Study Selection

**FIGURE 2:**
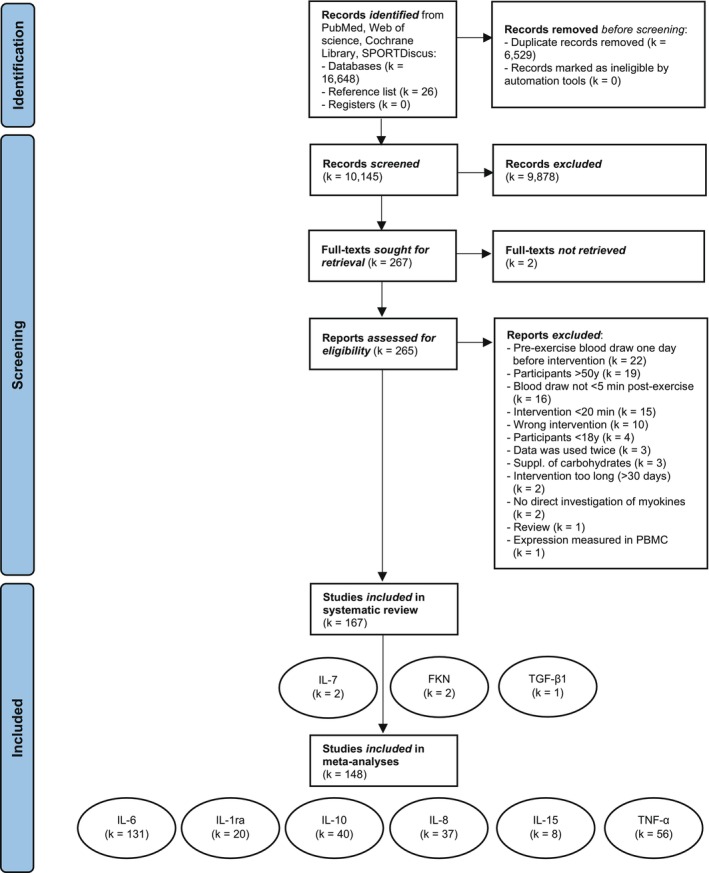
Flow diagram of the literature search. *K* is intended as the number of included studies. The number of effects (*k*_effects_) per meta‐analysis is presented in Figure 4.

Records identified through database searches were imported into Rayyan (https://www.rayyan.ai/) for blinded, independent screening by MR and FF. After removing duplicates, titles and abstracts were assessed, and ineligible studies were excluded. Disagreements were solved through discussion. Figure 2 summarizes the selection process.

### 2.4 Data Extraction

Data extraction was performed by MR. Information collected included study details (authors, design, sample size), myokine concentrations (pre‐ and post‐intervention with mean and standard deviation (SD)), participant characteristics (e.g., sex, age, body mass index (BMI)), and training status via peak oxygen consumption (VO_2_peak) and classification based on sample description. Exercise‐related variables ‐ type, load, duration, intensity, dose ‐ were also extracted. Intensity was classified as light, moderate, vigorous, or maximal according to ACSM guidelines [38]. Exercise *dose* was measured in MET·min. Additional data on sampling conditions (e.g., time of day, fasting state, sample type) were recorded. Details on the explanation and categorization of the different moderators are available in the Appendix S1.

The WebPlotDigitizer digitization program (https://automeris.io/WebPlotDigitizer/) was used to extract plotted data if the corresponding authors did not provide exact values or if the mean or SD was missing. Studies whose data were extracted using this program are indicated with an asterisk in Table S2 in the Appendix S1.

### 2.5 Risk of Bias Assessment

The risk of bias in individual studies was assessed using the ROBINS‐I V2 tool [39]. It consists of seven domains appraising the risk of bias: Risk of bias due to confounding (Domain 1), risk of bias in classification of interventions (Domain 2), risk of bias in selection of participants into the study or into the analysis (Domain 3), risk of bias due to deviations from intended interventions (Domain 4), risk of bias due to missing data (Domain 5), risk of bias arising from measurement of the outcome (Domain 6), and risk of bias in selection of the reported result (Domain 7). In this review, domain 2 was not considered as we used a pre–post interventional design. The individual domains were classified as either low, moderate, serious, or critical risk of bias. Overall, studies were rated as low risk if all domains showed low risk, moderate risk if at least one domain showed moderate risk, serious risk if at least one domain showed serious risk but none critical, and critical risk if any domain showed critical risk. The risk of bias assessment was done independently by FF and JG. The interrater coefficient for the overall risk of bias judgment was 0.92.

### 2.6 Effect Sizes

Standardized mean differences (SMDs) were calculated using Hedges' *g* to quantify changes in myokine concentrations, providing a bias correction for studies with small or unequal sample sizes [40]. All studies used within‐subject pre‐post designs, with positive values indicating an increase in the measured myokines. By convention, effect sizes of 0.2, 0.5, and 0.8 represent small, moderate, and large effects, respectively.

SMDs and their standard errors were computed following the guidelines by Borenstein et al. (section 4.15, p. 24) [41] and the Cochrane Handbook (chapter 23, section 23.2.7.2). A correlation coefficient of 0.80 between pre‐ and post‐values was imputed based on prior literature and available data [36, 42, 43, 44, 45, 46, 47, 48, 49]. To test the robustness of this assumption, sensitivity analyses were conducted with values ranging from 0.7 to 0.9. These analyses revealed no variations in main effects, outlier detection, trim‐and‐fill, and adjustments. However, several moderator effects varied across sensitivity analyses, warranting cautious interpretation of the following findings. Detailed results can be found in the limitations and the Appendix S1.

### 2.7 Statistics

All statistical analyses were performed in R (version 4.5.0) using the packages meta, metafor, and metaviz [50]. The complete R script and corresponding CSV files, including those used for sensitivity analyses, are publicly available via the Open Science Framework (https://doi.org/10.17605/OSF.IO/T6ZND).

Effect sizes were computed using a random‐effects model [51]. Separate meta‐analyses were conducted for each targeted myokine. Following the approach described by Viechtbauer and Cheung [50], potential outliers were identified through studentized deleted residuals and DFBETAS diagnostics. Data points were flagged as outliers if their studentized residuals exceeded ±3 and marked for closer examination if they exceeded ±1.96. DFBETAS values would have been used to determine the influence of these observations, with values above 1 indicating a potentially influential case in small to medium‐sized datasets, which was not the case in the present analyses.

### 2.8 Heterogeneity

Between‐study heterogeneity was quantified using *τ* ^2^ (the variance of true effects) based on Hedges' estimator [52]. Additionally, heterogeneity was evaluated using the *I* ^2^ statistic, which estimates the proportion of observed variance attributable to true effect differences rather than sampling error [53]. To reflect how results might vary across individual studies, prediction intervals (PIs) were calculated [54]. Confidence intervals and test statistics were adjusted using the Hartung–Knapp method [55, 56, 57].

Moderator analyses were conducted to explore potential sources of variability, including age, sex, BMI, training status, exercise type, load, duration, intensity, dose, V̇O_2_peak, time of day, sample type, fasting state, and risk of bias. Subgroup analyses addressed categorical moderators (e.g., sex, intensity), while continuous variables (e.g., age, duration) were examined via meta‐regression. Multivariate moderator analyses, conducted using the metafor package, focused on the exercise dose, investigating the effects of duration and intensity.

### 2.9 Small Study Effects

Potential small‐study effects, which may indicate publication bias, were initially evaluated through visual inspection of funnel plots. This was followed by Egger's test, with significance set at *p* < 0.100 (one‐tailed). If asymmetry was detected, the trim‐and‐fill method was applied to estimate the number of missing studies and adjust the overall Hedges' *g* accordingly [50, 58, 59].

### 2.10 Certainty of Evidence (GRADE Approach)

Following the analysis, MR and FJ independently evaluated the certainty of evidence using the GRADE (Grading of Recommendations, Assessment, Development, and Evaluation) framework [60]. Evidence quality was evaluated across eight domains. Five allowed for downgrading: risk of bias, inconsistency, indirectness, imprecision, and publication bias; and three domains allowed for upgrading: large magnitude of effect, presence of a dose–response relationship, and the likelihood of confounding factors reducing the observed effect. Downgrades were rated as none, serious (−1), or very serious (−2), while upgrades were scored as none, moderate (+1), or substantial (+2). The overall certainty of evidence for each meta‐analysis was then determined in accordance with GRADE criteria.

## 3 Results

### 3.1 Study Selection

A total of 16 648 records were identified across four databases. After removing duplicates, 10 145 studies remained for title and abstract screening. Of these, 267 full texts were assessed, with two not retrieved [61, 62] and 98 excluded studies (details in Appendix S1). Ultimately, 167 studies were included in the systematic review and 148 in the meta‐analyses (Figure 2). Nineteen studies could not be included in the meta‐analyses due to missing data.

### 3.2 Study Characteristics

This systematic review comprised six independent meta‐analyses. They covered a total of 294 endurance exercise interventions, most frequently measuring IL‐6, with 3622 participants ‐ predominantly male (80%) and on average 26.2 years old (range: 18–50 years old). Participants had a mean BMI of 23.4 ± 1.6 kg/m^2^ and an average V̇O_2_peak of 52.2 ± 8.2 mL/min/kg. Nearly half (47%) were trained, while 26% each were classified as active or untrained. Exercise sessions were primarily either running (58%) or cycling (42%), mostly continuous in nature (78%), and averaged 83 min in duration (ranging from 20 min to 24 h). Intensity varied, with most sessions being vigorous (53%), followed by moderate (32%), maximal (15%), and only one at light intensity. Most participants exercised fasted (69%) and in the morning (89%). Myokine levels were assessed in plasma (60%) or serum (40%). Details on all studies can be found in the Appendix S1 in Table S2.

### 3.3 Risk of Bias and Certainty of Evidence Assessment

**FIGURE 3:**
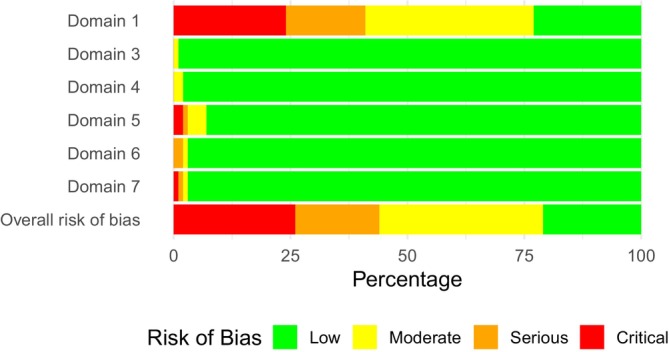
Results of the risk of bias assessment across all included studies. Risk of bias due to confounding (Domain 1), risk of bias in selection of participants into the study or into the analysis (Domain 3), risk of bias due to deviations from intended interventions (Domain 4), risk of bias due to missing data (Domain 5), risk of bias arising from measurement of the outcome (Domain 6), and risk of bias in selection of the reported result (Domain 7).

Thirty‐six studies were labeled to be at low risk of bias (21%), whereas 67 were deemed as moderate (40%). Thirty studies showed serious risk of bias (18%), and another 34 showed a critical risk of bias (20%). If one study was considered to be at serious or critical risk of bias, most inconsistencies occurred in the first, the confounding domain. The main reasons for the poor ratings in this domain were the lack of control of nutritional intake, caffeine, alcohol, or medication intake, or the lack of an indication of 24–48 h of rest before the intervention. Detailed results of the risk of bias assessment are presented in Table S3 (Appendix S1) and Figure 3.

The GRADE certainty of evidence was high for IL‐6, IL‐10, and IL‐8, low for IL‐1ra and IL‐15, and very low for TNF‐α. Detailed results can be found in Table S4 (Appendix S1).

### 3.4 Main Results

**FIGURE 4:**
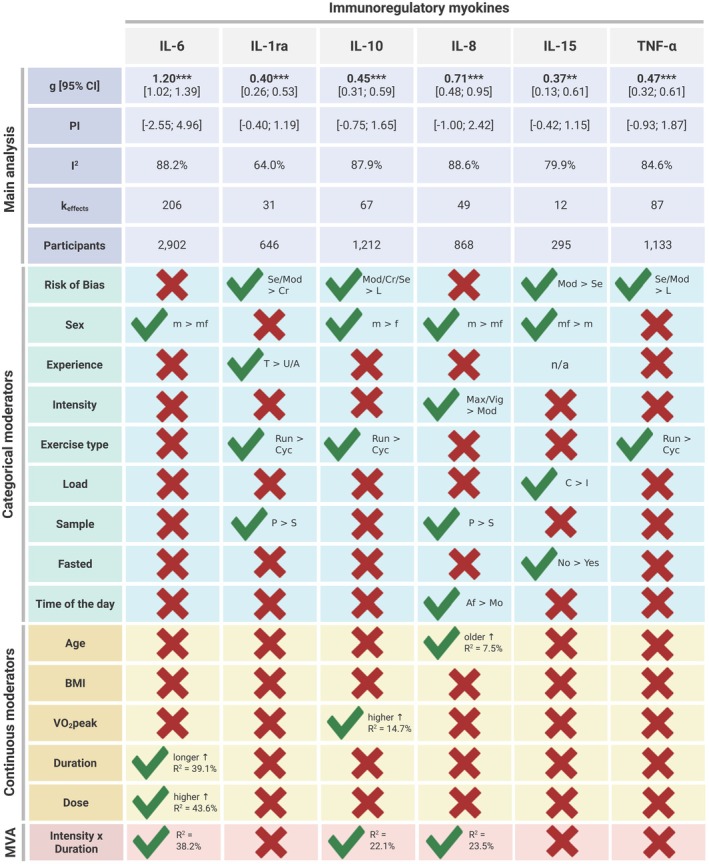
Main and moderator analysis results. ↑, higher effect; ↓, lower effect; A, active; Af, afternoon; BMI, Body Mass Index; C, continuous; CI, confidence interval; Cr, critical; Cyc, cycling; f, female; *g*, Hedges' *g*; I, intermittent; *I* ^2^, Heterogeneity between studies; IL, interleukin; *k*_effects_, number of effects; L, low; m, men; Max, maximal; mf, male and female; Mo, morning; Mod, moderate; MVA, multivariate analysis; n/a, not applicable; P, protein; PI, prediction interval; *R* ^2^, Goodness of fit; Run, running; S, serum; Se, serious; T, trained; U, untrained; Vig, Vigorous; V̇O_2_peak, peak oxygen consumption. **p < 0.01, ***p < 0.001.

Results are summarized in Figure 4. Overall, exercise induced a significant increase in all measured circulating myokines. The overall significant main effects ranged from small‐to‐moderate positive (IL‐15: *k*_effects_ = 12, *g* = 0.37, *p* < 0.010) to very large positive (IL‐6: *k*_effects_ = 206, *g* = 1.20, *p* < 0.001). The heterogeneity was large (PIs are presented in Figure 4), with a large part representing the variance of the true effect between studies (*I* ^2^ ranging from 64% to 88.2%). Asymmetry was detected visually and with the Egger's test for IL‐6 (*p* < 0.010), IL‐15 (*p* = 0.050), and TNF‐α (*p* < 0.010) (Figures S1, S5 and S6 in Appendix S1). However, the trim‐and‐fill analysis indicated that no additional record was needed to adjust the main effects. Detailed results can be found in Figure 4 and in the Appendix S1 (Table S5).

No meta‐analysis could be performed for IL‐7, FKN, and TGF‐β1 due to insufficient studies investigating those myokines. However, two studies, including four intervention groups investigating IL‐7, showed effect sizes ranging from *g* = 0.17 to 0.76 [63, 64]. Furthermore, two studies with three effects examined FKN with effect sizes ranging from *g* = 0.07 to 0.56 [63, 65]. One study looked into TGF‐β1 with four pooled intervention groups showing a large effect (*g* = 0.64) [66].

### 3.5 Moderator Analysis

The main moderators are presented below, while more detailed results on each post hoc analysis can be found in the Appendix S1.

Risk of bias emerged as a significant moderator in IL‐1ra, IL‐10, IL‐15, and TNF‐α. For IL‐1ra, studies rated as critical (*g* = 0.13) had significantly lower effect sizes compared to moderate (*g* = 0.45, *p* < 0.001) and serious (*g* = 0.61, *p* < 0.010). In contrast, the pattern was reversed for IL‐10 and TNF‐α. Studies with a low risk of bias (*g* = 0.00) showed significantly lower effect sizes than those with moderate (*g* = 0.71, *p* < 0.010), serious (*g* = 0.48, *p* < 0.050), or critical risk of bias (*g* = 0.50, *p* < 0.050) for IL‐10. Additionally, studies with low risk (*g* = 0.15) of bias assessing TNF‐α had significantly lower effect sizes compared to studies at moderate (*g* = 0.58, *p* < 0.010) or serious risk of bias (*g* = 0.70, *p* < 0.010). For IL‐15, studies at moderate risk of bias (*g =* 0.42) showed higher effect sizes compared to studies at serious risk (*g* = −0.51, *p* < 0.001). However, no study showed low or critical risk of bias.

Sex also significantly influenced outcomes in four of the six analyses. Men exhibited larger responses than mixed groups in IL‐6 (men: *g* = 1.27, mixed: *g* = 0.83, *p* < 0.010) and IL‐8 (men: *g* = 0.92, mixed: *g* = 0.37, *p* < 0.050). For IL‐10, men (*g* = 0.60) showed bigger effect sizes compared to women (*g* = 0.12, *p* < 0.050). Additionally, mixed groups (*g* = 0.58) exhibited higher effects compared to male groups (*g* = 0.18, *p* < 0.050) in IL‐15.

Training status significantly moderated the effects of IL‐1ra only, with trained individuals (*g* = 0.57) showing more pronounced responses than untrained (*g* = 0.23, *p* < 0.050) or moderately active ones (*g* = 0.09, *p* < 0.001). Exercise intensity significantly influenced IL‐8 levels: the effects observed at maximal (*g* = 0.96, *p* < 0.010) and vigorous (*g* = 0.85, *p* < 0.050) intensity were significantly higher compared to exercise performed at moderate intensity (*g* = 0.44). Regarding exercise modality, running (run) led to greater increases in IL‐10 (run: *g* = 0.63, cycl.: *g* = 0.24, *p* < 0.010), IL‐1ra (run: *g* = 0.52, cycl.: *g* = 0.21, *p* < 0.010), and TNF‐α (run: *g* = 0.65, cycl.: *g* = 0.36, *p* < 0.010) compared to cycling (cycl.). Continuous protocols (*g* = 0.42) yielded stronger IL‐15 responses than intermittent ones (*g* = −0.03; *p* < 0.001). IL‐1ra (pl: *g* = 0.52, se: *g* = 0.17, *p* < 0.010) and IL‐8 (pl: *g* = 0.84, se: *g* = 0.44, *p* < 0.050) effects were more pronounced when measured in plasma (pl) rather than serum (se). Nutritional status influenced IL‐15 responses, with lower effects seen in fasted participants (*g* = 0.00) compared to non‐fasted individuals (*g* = 0.63, *p* < 0.010). Timing mattered as well; IL‐8 responses were stronger during afternoon sessions (*g* = 1.17) compared to morning ones (*g* = 0.50, *p* < 0.010).

While BMI was not a significant moderator in any myokine, age and fitness level moderated some effects. Older individuals had greater IL‐8 responses (*R* ^2^ = 7.46%, *p* < 0.050), and higher VO_2_peak was associated with larger IL‐10 effects (*R* ^2^ = 14.66%, *p* < 0.010). Lastly, for IL‐6, longer exercise sessions correlated with stronger effects (*R* ^2^ = 39.08%, *p* < 0.001), and a higher exercise dose led to higher effects (*R* ^2^ = 43.60%, *p* < 0.001).

Multivariate analyses showed that intensity and duration together could significantly explain 38.23% of the variance of the effect of IL‐6 (*p* < 0.001), 22.08% of IL‐10 (*p* < 0.050), and 23.49% of IL‐8 (*p* < 0.050).

## 4 Discussion

This systematic review and meta‐analysis is the first to characterize the immediate effects of acute endurance exercise on IL‐6, IL‐10, IL‐1ra, IL‐8, IL‐15, and TNF‐α. Overall, acute exercise induced positive responses across all examined myokines, with effect sizes ranging from small‐to‐moderate for IL‐15 (*g* = 0.37) to very large for IL‐6 (*g* = 1.20). Among the most noticeable moderator analyses, one can note that IL‐8 was the only one significantly influenced by exercise intensity. On the other hand, IL‐6 was the only one showing stronger effects with longer duration. However, the magnitude of IL‐6, IL‐10, and IL‐8 responses was significantly modulated by the overall exercise *dose*, reflecting the combined influence of intensity and duration. One can also note that running induced greater increases in IL‐1ra, IL‐10, and TNF‐α compared to cycling.

### 4.1 Metabolic Demand

The pronounced IL‐6 response to acute endurance exercise reflects its high metabolic demands. It was shown that the IL‐6, as well as IL‐10 and IL‐8 responses, are significantly moderated by the exercise *dose* further underlining the increasing energy demand with increasing intensity, duration, and muscle mass involved [67]. As glycogen stores are depleted during exercise, the body activates hormonal, enzymatic, and cellular mechanisms to mobilize alternative energy sources and maintain energy balance in the short term. Acting as a key energy sensor, IL‐6 promotes the mobilization of energy reserves by stimulating lipolysis in adipose tissue and glycogenolysis in the liver, thereby increasing the availability of free fatty acids and glucose, respectively (Figure 1) [68, 69]. These substrates are essential for meeting the heightened energy requirements during exercise. Notably, this metabolic activation appears to be significantly greater during endurance exercise compared to resistance exercise, which elicits only moderate‐to‐large IL‐6 responses, as shown in our previous meta‐analysis [33]. Moreover, while the immediate increase in circulating IL‐6 is likely driven primarily by pre‐existing protein rapidly released from muscle, IL‐6 mRNA expression in muscle tissue also rises following acute endurance exercise, suggesting a more sustained regulation at the transcriptional level [46, 70, 71].

Furthermore, several studies have shown that exercising in an overall glycogen‐depleted state (thus, leading to even less energy availability) results in a greater increase in IL‐6, supporting the idea of IL‐6 as an energy sensor [72]. During acute exercise, untrained muscles rely heavily on glycogen as a substrate [11]. This allows trained muscles to use fat more efficiently during intense or prolonged exercise, thereby consuming less glycogen compared to untrained muscles [11]. Existing literature also indicates that when muscle glycogen levels are low, IL‐6 transcription is upregulated, resulting in a greater relative IL‐6 production for the same exercise workload compared to conditions of high glycogen availability. Consequently, as trained individuals deplete less glycogen, they are likely to exhibit a blunted acute IL‐6 response following exercise at a given intensity compared to untrained individuals [11]. Although training status did not reach statistical significance in this meta‐analysis, untrained participants displayed higher IL‐6 effect sizes than trained or active individuals (*p* = 0.160).

IL‐15 was the only myokine whose response was significantly influenced by participants' nutritional state, with fasted individuals exhibiting greater increases than non‐fasted individuals. By enhancing muscle oxidative metabolism and insulin sensitivity [73], IL‐15 improves the body's metabolic flexibility and energy efficiency. This effect appears particularly relevant during exercise in a fasted state, where elevated IL‐15 levels may help to compensate for reduced energy availability. As noted above, IL‐6, being glycogen‐dependent, might also be expected to respond to fasting. However, the fasting periods in the included studies were relatively short (eight to 12 h), likely sufficient to deplete some liver glycogen but not substantially affect muscle glycogen. Influencing muscle glycogen would require prolonged fasting (> 24 h) or a low‐carbohydrate diet, conditions not recorded in the current analysis. Therefore, it is possible that IL‐6 responses are more closely dependent on muscle glycogen than general glycogen.

Engaging more muscle during exercise increases glycogen utilization across more muscle cells, likely reflecting the higher overall metabolic demand of full‐body activity. This may help explain why running, which engages more muscle groups than cycling, results in higher IL‐6 levels. The notion that muscle mass influences myokine release is supported by the larger effect sizes seen after running compared to cycling [67].

Exercise *dose* (and thus, the dose–response relationship) was assessed in two different ways ‐ either by total exercise dose (in MET·min) as a continuous moderator or by the interaction between intensity and duration in a multivariate analysis. The moderator analysis revealed that *dose* ‐ whether modeled as a continuous variable in MET·min or as a combination of intensity and duration in a multivariate analysis ‐ accounts for approximately 40% of the variance in IL‐6 responses across the included studies. This underscores the substantial influence of exercise intensity and duration on IL‐6 concentrations. However, only the multivariate analysis revealed a significant effect on IL‐10 and IL‐8 as well. This shows in particular that MET·min is the most robust indicator of metabolic demand over time, as it also combines information about energy consumption during exercise together with the duration of the activity. It may be that the dose–response relationship is primarily driven by a metabolic demand, thereby influencing mainly myokines directly involved in energy metabolism (such as IL‐6). This would further highlight IL‐6's unique role as an energy sensor and illustrate the diverse functions of exercise‐induced myokines.

Although this is first a metabolic response, the rise in IL‐6 and further myokines post‐exercise has additional far‐reaching effects, particularly at the immunological level.

### 4.2 Immunological Alterations

IL‐6 not only affects energy metabolism but is also involved in the immune system's response to physical activity. By activating and promoting the migration of NK cells into the blood, IL‐6 leads to heightened immune surveillance to fight possible pathogens [74]. Additionally, IL‐6 is involved in the release of IL‐10 and IL‐1ra [20]. IL‐10 promotes the recruitment and expansion of Tregs, which in turn secrete additional IL‐10 [26], establishing a positive feedback loop that reinforces immune regulation and suppresses inflammation. Our results support this finding as there is a significant increase seen in IL‐1ra and IL‐10 counterbalancing the rise in IL‐6, exerting further anti‐inflammatory effects by blocking the IL‐1 receptor [75], and orchestrating the exercise‐induced immune response (IL‐10 [76] in Figure 1). It should also be noted that Treg differentiation is induced by additional exercise‐derived metabolites not included in the meta‐analysis, particularly kynurenine metabolites. Kynurenine and kynurenic acid are ligands of the aryl hydrocarbon receptor and promote the differentiation of naïve CD4^+^ T cells into regulatory T cells [77, 78]. Although exercise‐induced increases in IL‐6 activate indoleamine 2,3‐dioxygenase, thereby enhancing the conversion of tryptophan to kynurenine, establishing a perfect linear relationship between post‐exercise IL‐6 and IL‐10 levels alone remains challenging.

When comparing the findings with our meta‐analysis on resistance exercise, similar effects were observed for IL‐1ra. However, in contrast to the present results, no significant changes in IL‐10 were detected following resistance exercise [33], possibly due to delayed activation. Additionally, unlike IL‐6, which is primarily secreted by muscle cells, IL‐10 is not only released by muscle cells but also by monocytes and lymphocytes in response to exercise [79, 80]. Since endurance exercise typically triggers a stronger activation of leukocytes due to its sustained cardiovascular load [81], it is more likely to elicit a noticeable increase in IL‐10. However, it should be noted that this meta‐analysis, as well as the previous one [33], was limited to measurements obtained within 5 min after exercise cessation. It is, therefore, possible that IL‐10 responses occur later during recovery and were not captured in the available data. Given that IL‐6 release precedes IL‐10 upregulation and that delayed anti‐inflammatory signaling has been proposed in relation to exercise‐induced immune responses and the kynurenine pathway [78], future studies should investigate the kinetics of IL‐10 following resistance exercise. Notably, metabolically demanding resistance exercise may elicit immune responses comparable to endurance exercise; however, further controlled studies are required to confirm potential anti‐inflammatory effects, including delayed IL‐10 responses. For example, preliminary findings from our research group indicate that high‐intensity interval resistance exercise results in greater changes in immune inflammation indices (i.e., neutrophil‐to‐lymphocyte ratio, platelet‐to‐lymphocyte ratio, and systemic immune inflammation index) 45 min post‐exercise compared to traditional resistance exercise [82]. While these observations are indirect, they support the possibility that higher‐intensity resistance exercise may be associated with delayed anti‐inflammatory responses.

Our results also show a clear IL‐8 increase following acute endurance exercise, with effect sizes up to *g* = 3.212. The release of IL‐8 is triggered by metabolic stress and possible microtrauma in the muscle tissue [83], recruiting granulocytes and promoting and coordinating muscle regeneration [32]. The increase in IL‐8 concentration is supported by an increase in its muscle mRNA expression [46, 70, 71]. Among the investigated myokines, IL‐8 was the only one whose response was significantly influenced by the time of day the exercise session was conducted, with evening sessions eliciting greater effects than those performed in the morning. Furthermore, individual studies included in this meta‐analysis also reported higher IL‐6 responses in the evening compared to the morning [84]. However, this diurnal variation was not statistically significant in the overall meta‐analytic model for IL‐6, suggesting that the time‐of‐day effect may not be consistent across all study protocols. One explanation for this potential diurnal variation could, however, be changes in catecholamines, which are activated due to a metabolic demand as well, as they greatly impact immunological responses by inhibiting or promoting different immune cells like Th1 and Th2 cells, as well as NK cells or monocytes, and, therefore, subsequently influencing cytokines [85]. They promote the release of IL‐8 and IL‐6 from various cells and tissues [86, 87] while inhibiting Th1 cells and their cytokine production [85, 87]. Given that endurance exercise performed in the evening induces a greater epinephrine response than morning sessions [84], this temporal difference may partly account for the heightened exerkine release observed during evening exercise.

Although a meta‐analysis was not possible due to limited data, two studies reported an immediate small‐to‐moderate increase in IL‐7 following acute endurance exercise [63, 64]. As IL‐7 plays a central role in T‐cell homeostasis, especially in memory T cells and naïve T cells [31], a redistribution of T cells following exercise could lead to the release of IL‐7 from the working muscle to maintain a balance in T lymphocytes. Like IL‐7, FKN [63] and TGF‐β1 [66] were also reported to increase in response to endurance exercise, but each with only one study showing a moderate and large effect size, respectively. By increasing monocytes and cytotoxic NK cells, FKN can have a positive impact, for example, on reducing tumor cell growth [86]. By inducing the differentiation of Tregs from naïve T cells, TGF‐β1 regulates the immune response in an anti‐inflammatory way [88].

IL‐15, like IL‐6, is involved in recruiting NK cells and, therefore, exerting further immunoregulatory effects [89]. Our findings display a significant increase in IL‐15 following acute endurance exercise (*g =* 0.37). However, interestingly, in almost all individual studies, the effects and samples were too small to reach significance. Besides, studies investigating IL‐15 mRNA expression in the skeletal muscle show small‐to‐moderate negative effects following acute endurance exercise [70, 71, 90]. This suggests a difference in response between IL‐15 concentration in the blood and its mRNA expression in muscle tissue, potentially due to differences in recruitment of secreting cells (e.g., muscle vs. immune cells) [91].

Although the literature suggests that acute exercise increases IL‐6 without a concurrent rise in TNF‐α [11], this pattern was not observed in the present meta‐analysis or on the one assessing resistance exercise [33]. This increase in TNF‐α blood concentration is supported by the significant increase in skeletal muscle mRNA expression underlining its sustainability [46]. This transient increase in TNF‐α is most likely due to leukocytosis, which occurs after acute (endurance) exercise due to muscle damage and the resulting need for tissue repair [92]. The role of TNF‐α is to promote the transmigration of immune cells into the (muscle) tissue, known as diapedesis [92]. TNF‐α activates the vascular endothelium by increasing the expression of adhesion molecules such as intercellular adhesion molecule (ICAM)‐1 or vascular cell adhesion molecule (VCAM)‐1, which enable leukocytes to adhere to the vessel walls [93]. In addition, it increases permeability and recruits transmigration factors to allow the migration of immune cells through the endothelium [93]. In this way, the acutely increased TNF‐α response enables diapedesis, a natural, temporary inflammatory response aimed at healing and adapting muscle tissue.

### 4.3 Population‐Dependent Effects

IL‐8 was the only myokine responding to age, with older participants showing higher effects to an acute endurance exercise session compared to younger participants. With increasing age, there is a state of chronically slightly increased inflammatory activity in the body (“inflammaging”) [94, 95]. As a result, certain signaling pathways that promote pro‐inflammatory cytokines such as IL‐8 are more sensitive or already pre‐activated. The body might react to acute stimuli such as physical exercise with an increased IL‐8 response because it is already in a pre‐activated state. Still, the range was rather small (18–50 years), which also explains why the other myokines did not respond to this moderator.

Regarding sex, men exhibited larger responses than mixed groups in IL‐6 and IL‐8. For IL‐10, men showed bigger effect sizes compared to women, while mixed groups exhibited higher effects compared to male groups in IL‐15. There are well‐documented sex‐related differences in body composition, as men tend to have a higher lean body mass than women [96]. As muscle mass is positively associated with myokine release [67], it can be assumed that men exhibit higher effects than women [97]. This seems especially true, as expression levels of most myokines were similar between sexes [98]. Sex hormones may also play a crucial role in modulating immune responses. Acute bouts of exercise can further elevate testosterone levels [99, 100], potentially amplifying its regulatory effects on various myokines. However, it is important to acknowledge that the majority of included studies primarily investigated male participants, raising the question of whether observed sex‐related differences reflect true biological variation or are instead a consequence of the limited representation of female participants in the available literature.

BMI was not a significant moderator in any of the myokines, indicating a BMI‐independent effect of endurance exercise on immunoregulatory myokines. Thus, endurance exercise seems to induce beneficial immunoregulatory myokine responses regardless of an individual's body composition. It essentially supports the idea that exercise is an effective immunomodulatory stimulus across diverse populations, which strengthens the case for recommending endurance training as part of preventive and therapeutic programs without BMI‐specific restrictions. However, it should be noted that, due to eligibility requirements (i.e., only healthy individuals), the BMI ranged only from 19.99 kg/m^2^ to 31.90 kg/m^2^. Therefore, our data are limited and do not include, for example, very thin individuals (< 18 kg/m^2^) or those with severe obesity (Classes II or III). It is possible that chronic low‐grade inflammation in individuals with obesity, reflected by elevated baseline levels of C‐reactive protein, TNF‐α, or IL‐6 [101], may alter their response to acute (endurance) exercise. Only a few studies have examined this with regard to an intensity‐dependent effect, and further research is warranted to clarify the acute exercise effects in this population. Finally, BMI was based on cohort means and is a limited measure of body composition; thus, these results should be interpreted with caution.

### 4.4 Additional Moderators

A significant difference in effect sizes between serum and plasma samples was observed for IL‐1ra and IL‐8, with studies utilizing plasma reporting greater effects compared to those analyzing serum. This discrepancy is likely attributable to the different protein concentrations, with serum containing fewer proteins due to the process of coagulation [102], therefore, potentially leading to higher effects seen in plasma compared to serum.

Although the moderator analysis for risk of bias yielded significant findings, a consistent directional pattern across the examined myokines was not evident. For TNF‐α and IL‐10, studies with low risk of bias reported the smallest effect sizes, suggesting a potential overestimation of effects in studies with higher risk of bias ‐ possibly due to confounding factors such as uncontrolled medication use, caffeine or alcohol intake, or recent physical activity prior to the intervention. In contrast, the opposite trend was observed for IL‐1ra and IL‐15, where studies with a lower risk of bias reported larger effect sizes.

### 4.5 Therapeutic Importance

According to the concept of hormesis, whereby greater acute stimuli may translate into long‐term immunoregulatory benefits, it can be assumed that although this meta‐analysis focused on acute exercise responses, all tested forms of endurance activity induce longer‐term anti‐inflammatory effects. Notably, increased exercise duration and intensity lead to a more pronounced myokine release (IL‐6, IL‐10, IL‐8), while excessively prolonged efforts, such as ultra‐endurance events, may have detrimental effects on immune function due to an overactivation [103]. Moreover, larger effect sizes were observed when greater muscle mass was recruited (IL‐10, IL‐1ra, TNF‐α), indicating that whole‐body exercises (e.g., running) may enhance anti‐inflammatory responses more effectively than isolated muscle group training. These findings support the integration of full‐body endurance activities into regular training regimens. The observed intensity‐independent effects further suggest that exercise prescriptions can be adapted to meet the needs of specific populations. For example, high‐intensity interval training (HIIT) may be advantageous for time‐constrained individuals or cardiovascular patients, whereas moderate continuous training (MCT) may be more appropriate for older adults or those with orthopedic limitations ‐ both approaches provide comparable immunoregulatory benefits shown by the current analysis.

However, it should be considered that the main driver of myokine release is the metabolic demand represented by the exercise *dose* in MET·min. Therefore, increasing dose, either by increasing the energy consumption of the (endurance) exercise session or by increasing the exercise duration, will also lead to a greater release of IL‐6, IL‐10, and IL‐8, leading to subsequent anti‐inflammatory immune responses.

### 4.6 Limitations and Research Perspectives

One limitation is that we did not assess muscle‐derived cytokines only, but all circulating cytokines, including those expressed by other secretory organs as well [33]. Nevertheless, changes measured in muscle tissue show the same positive effects as those measured in blood, although there are not enough studies to perform meta‐analytical research on mRNA data and a possible delay in mRNA expression must be considered. Additionally, if myokines are viewed as exerkines, their assessment in blood remains valid. Still, more studies on muscle tissue are necessary, assessing changes in immune cells at the same time, to deduce the proportion of both in the systemic rise in myokines.

Although a clear dose–response relationship could be shown for IL‐6, IL‐10, and IL‐8, the only investigated timepoint was immediately post‐exercise. Therefore, future meta‐analytic research should also focus on later myokine measurements to provide insights into how this relationship might change and for how long changes can be seen before concentrations return to pre‐exercise values.

It should also be noted that, although this dose–response relationship has been demonstrated, most available studies involve moderate‐to‐vigorous intensity and moderate duration. As a result, studies at the lower and upper extremes are lacking, meaning that a small number of such studies could disproportionately influence the overall findings.

One should acknowledge as well that several moderators varied across sensitivity analyses (Appendix S1—Time of the day: IL‐6; IL‐1ra; Risk of Bias: IL‐10, IL‐1ra; Sex: IL‐15; Experience: IL‐1ra, IL‐8; Exercise type: TNF‐α; Age: IL‐8). Therefore, their results should be considered with caution.

Additionally, the majority of the included studies examined male participants (80%) and only a few studies looked into female participants (20%). Therefore, sex‐related findings must be interpreted cautiously, and future research should specifically investigate myokine changes in women. It should also be noted that the difference in overall risk of bias across the studies is mainly driven by the confounding domain. This domain is the most heterogeneous one, and due to the structure of the ROBINS‐I tool (the overall judgment corresponds to the worst rating across the seven domains), this domain is the most influential one. Therefore, to enhance the overall quality and generalizability of the included studies, more comprehensive reporting and control of potential confounding variables is essential. Factors such as dietary intake, standardized warm‐up procedures, and pre‐intervention physical activity were often insufficiently controlled, thereby increasing the risk of bias. Future research should aim to rigorously document and regulate these variables, as they may substantially influence outcomes. Additionally, studies with larger sample sizes are required to reduce the likelihood of inflated effect size estimates and to improve the statistical robustness of the findings.

## 5 Conclusion

This systematic review and meta‐analysis provides the first comprehensive synthesis of the immediate effects of acute endurance exercise on immunoregulatory myokines in healthy adults. The findings demonstrate that acute endurance exercise increases circulating levels of IL‐6, IL‐10, IL‐1ra, IL‐8, IL‐15, and TNF‐α, with effect sizes ranging from small to moderate to very large. These responses are primarily driven by an exercise‐induced metabolic demand, leading to pronounced immunological alterations. Central to this process is IL‐6, acting both as an energy sensor and an anti‐inflammatory immune trigger. It was shown that myokine responses depend on duration, intensity, muscle mass, and training status, enabling a possible control of myokine release that can be used for therapeutic purposes in a variety of settings. However, although specific training characteristics may optimize myokine release, this systematic review and meta‐analysis demonstrates that meaningful increases in immunoregulatory myokines occur even when these conditions are not met. Collectively, these findings underscore endurance exercise as a robust and accessible stimulus for immunomodulation and support its broader integration into therapeutic strategies for neurological, psychological, and cardiovascular diseases.

## Author Contributions

**Miriam Ringleb:** conceptualization, formal analysis, methodology, writing – original draft. **Fabian Fabritius:** methodology, writing – review and editing. **Jakob Godde:** methodology, writing – review and editing. **Christian Puta:** supervision, writing – review and editing. **Wilhelm Bloch:** supervision, writing – review and editing. **Florian Javelle:** conceptualization, formal analysis, methodology, supervision, writing – review and editing.

## Funding

The authors have nothing to report.

## Conflicts of Interest

The authors declare no conflicts of interest.

## Supporting information


**Appendix S1:** fsb271536‐sup‐0001‐AppendixS1.docx.

## Data Availability

The data that support the findings of this study are openly available on Open Science Framework at https://doi.org/10.17605/OSF.IO/T6ZND.
