# Supplementary material for: Circulating Myokine Responses to Acute Endurance Exercise and Their Role in Immunoregulation: A Systematic Review and Meta‐Analysis
Source: FASEB J. 2026 Feb 9;40(4):e71536. doi: 10.1096/fj.202504780R (PMC12885107; doi:10.1096/fj.202504780R)
Supplement: Supplementary file 1 — Appendix S1: fsb271536‐sup‐0001‐AppendixS1.docx. [file FSB2-40-e71536-s001.docx]

**Supplementary Material**

**Circulating Myokine Responses to Acute Endurance Exercise** **and their Role in Immunoregulation: A Systematic Review and Meta-Analysis**

Miriam Ringleb^1,2,3^, Fabian Fabritius^1^, Jakob Godde^1^, Christian Puta^2,3,4^, Wilhelm Bloch^5^, Florian Javelle^1^

1 NeuroPyschoImmunology research unit, Department for Molecular and Cellular Sports Medicine, Institute of Cardiovascular Research and Sports Medicine, German Sport University Cologne, Cologne, Germany

2 Department of Sports Medicine and Health Promotion, Friedrich­Schiller­University Jena, Jena, Germany

3 Center for Interdisciplinary Prevention of Diseases related to Professional Activities, Friedrich­Schiller­University Jena, Jena, Germany

4 Center for Sepsis Control and Care (CSCC), Jena University Hospital, Friedrich-Schiller­University Jena, Jena, Germany

5 Department for Molecular and Cellular Sports Medicine, Institute of Cardiovascular Research and Sports Medicine, German Sport University Cologne, Cologne, Germany

**Table of Contents**

[Table S1 Exclusion reasons after full text search 4](#_Toc205899741)

[Data extraction and categorization of the moderators 6](#_Toc205899742)

[Table S2 Study Characteristics 7](#_Toc205899743)

[Table S3 Risk assessment 28](#_Toc205899744)

[Table S4 Certainty of evidence 35](#_Toc205899745)

[Table S5 Main results meta-analyses 36](#_Toc205899746)

[Plots IL-6 37](#_Toc205899747)

[Plots IL-1ra 38](#_Toc205899748)

[Plots IL-10 39](#_Toc205899749)

[Plots IL-8 40](#_Toc205899750)

[Plots IL-15 41](#_Toc205899751)

[Plots TNF-α 42](#_Toc205899752)

[Results moderator analysis 43](#_Toc205899753)

[Risk of bias 43](#_Toc205899754)

[Sex 44](#_Toc205899755)

[Experience 45](#_Toc205899756)

[Intensity 46](#_Toc205899757)

[Exercise type 47](#_Toc205899758)

[Load 48](#_Toc205899759)

[Sample 49](#_Toc205899760)

[Fasted 50](#_Toc205899761)

[Time of the day 51](#_Toc205899762)

[Age 52](#_Toc205899763)

[BMI 52](#_Toc205899764)

[VO_2_max 52](#_Toc205899765)

[Duration 52](#_Toc205899766)

[Results multi variate analysis 54](#_Toc205899767)

[IL-6 54](#_Toc205899768)

[IL-10 55](#_Toc205899769)

[TNF-α 56](#_Toc205899770)

[IL-1ra 57](#_Toc205899771)

[IL-8 58](#_Toc205899772)

[IL-15 59](#_Toc205899773)

[Results sensitivity analysis 60](#_Toc205899774)

[Prisma Checklist 2020 62](#_Toc205899775)

[References 65](#_Toc205899776)

# Table S1 Exclusion reasons after full text search

| Blood draw not immediately post-exercise | |
| --- | --- |
|  | Skinner et al. (1) |
|  | Tamura et al. (2) |
|  | Ueda et al. (3) |
|  | Faraldi et al. (4) |
|  | Li et al. (5) |
|  | Comassi et al. (6) |
|  | Weimann et al. (7) |
|  | Peeling et al. (8) |
|  | Larsen et al. (9) |
|  | Ostapiuk-Karolczuk et al. (10) |
|  | Badenhorst et al. (11) |
|  | Landers-Ramos et al. (12) |
|  | Sanderson et al. (13) |
|  | Stacey et al. (14) |
|  | Kimura et al. (15) |
|  | Kraemer et al. (16) |
| Resting blood sample not immediately pre-exercise but on another day | |
|  | Vassalle et al. (17) |
|  | Baygutalp et al. (18) |
|  | Santos et al. (19) |
|  | Jeukendrup et al. (20) |
|  | Suzuki et al. (21) |
|  | Brestoff et al. (22) |
|  | Drenth et al. (23) |
|  | Chiu et al. (24) |
|  | Schild et al. (25) |
|  | Santos et al. (26) |
|  | Nickel et al. (27) |
|  | Rowlands et al. (28) |
|  | Ostrowski et al. (29) |
|  | Suzuki et al. (30) |
|  | Stelzer et al. (31) |
|  | Ostrowski et al. (32) |
|  | Ostrowski et al. (33) |
|  | Donnikov et al. (34) |
|  | Deetjen et al. (35) |
|  | Bekos et al. (36) |
|  | Villar-Fincheira et al. (37) |
|  | Philippe et al. (38) |
| Participants >50y old | |
|  | Siegel et al. (39) |
|  | Cappuccilli et al. (40) |
|  | Benedetti et al. (41) |
|  | Mendham et al. (42) |
|  | Yargic et al. (43) |
|  | Hew-Butler et al. (44) |
|  | Panagoulias et al. (45) |
|  | Jouffroy et al. (46) |
|  | Bonsignore et al. (47) |
|  | Cairns et al. (48) |
|  | Jee et al. (49) |
|  | Arakawa et al. (50) |
|  | Catoire et al. (51) |
|  | Perry et al. (52) |
|  | Mendham et al. (53) |
|  | Steward et al. (54) |
|  | Vaisberg et al. (55) |
|  | Nieman et al. (56) |
|  | Sprenger et al. (57) |
| Participants <18y old | |
|  | Jürimäe et al. (58) |
|  | Hayashi et al. (59) |
|  | Nieman et al. (60) |
|  | Yamada et al. (61) |
| Review | |
|  | Siegel et al. (62) |
| Intervention <20 min | |
|  | Mucci et al. (63) |
|  | Stajer et al. (64) |
|  | Kon et al. (65) |
|  | Mohamed et al. (66) |
|  | Steinberg et al. (67) |
|  | Said et al. (68) |
|  | Kapilevich et al. (69) |
|  | Rhibi et al. (70) |
|  | Kröpfl et al. (71) |
|  | Silva‑Cavalcante et al. (72) |
|  | Rivier et al. (73) |
|  | Kapilevich et al. (74) |
|  | Mendez-Gutierrez et al. (75) |
|  | Cabral-Santos et al. (76) |
|  | Skarpanska-Stejnborn et al. (77) |
| Intervention too long (>30 days) | |
|  | Keohane et al. (78) |
|  | Zebrowska et al. (79) |
| Supplementation of carbohydrates | |
|  | Möbius-Winkler et al. (80) |
|  | Reichel et al. (81) |
|  | Peeling et al. (82) |
| Expression measured in PBMC | |
|  | Northoff et al. (83) |
| Data reported in another study that has been in included already | |
|  | Sugama et al. ((84) |
|  | Cox et al. (85) |
|  | Snipe et al. (86) |
| Not endurance exercise/max Test | |
|  | Crabb et al. (87) |
|  | Zebrowska et al. (88) |
|  | Blumkaitis et al. (89) |
|  | Huminska-Lisowska et al. (90) |
|  | Reihmane et al. (91) |
|  | Dogru et al. (92) |
|  | Keller et al. (93) |
|  | Limprasertkul et al. (94) |
|  | Zoladz et al. (95) |
|  | Alizadeh & Alizadeh (96) |
| No direct investigation of myokines | |
|  | Nielsen et al. (97) |
|  | Pussieldi et al. (98) |

# Data extraction and categorization of the moderators

Intensity was classified into either light, moderate, vigorous, or maximal intensity according to ACSM guidelines (99) based on either percentage of maximal heart rate, percentage of VO_2_max, or perceived exertion.

The exercise dose was computed in MET·min. The MET value per session was based on the ACSM guidelines (99) and multiplied with the exercise duration in minutes.

The training status was classified in trained, active, and untrained. Participants were categorized as trained if they trained for more than six weeks with at least three trainings per week. This approach allowed participants who completed a chronic training intervention to be classified as "trained" during the post-intervention acute exercise session, as they had undergone structured training beforehand. Participants that do no exercise on a regular basis but are physically active were classified as active and those, not training or being physically active at all, were deemed as untrained.

The time of day at which the exercise session was conducted was categorized in “morning” and “afternoon”. All sessions that were finished until 12am were classified as being done in the “morning” and every session taking place after 12am was classified as “afternoon” session. Those exercise interventions that took all day due to their ultra-endurance character were excluded from this classification.

To simplify the categorization regarding the fasting state of the participants, all intervention groups were classified as either “yes”, the participants were fasted for at least 8h overnight or “no”, they were not fasted for at least 8h or longer.

# Table S2 Study Characteristics

| Study | Risk | N | Sex | Age (y) | BMI (kg/m^2^) | VO_2_max (kg/min/ml) | Type of exercise | Load | Duration (in min) | Intensity | Dose (MET x  min) | Fasted | Time of day | Serum/Plasma |
| --- | --- | --- | --- | --- | --- | --- | --- | --- | --- | --- | --- | --- | --- | --- |
| Acevedo et al. (100) | Low | 10 | m | 40.7 | 22.83 | 45.31 | n/a | Continuous | n/a | Moderate | n/a | No | Morning | Serum |
| Akerstrom et al. (101) | Moderate | 6 | m | 26 | 22.4 | 50.4 | Cycling | Continuous | 180 | Vigorous | 970 | Yes | Morning | Plasma |
| Aktitiz et al. (102) | Moderate | 20 | m | 29.1 | 24 | 47.94 | Cycling | Intermittent | 22.5 | Maximal | 198 | Yes | Afternoon | Plasma |
| Alfaro-Magallanes et al.* (103) a | Low | 16 | f | 25.3 | 21.34 | 47.4 | Running | Intermittent | 36 | Vigorous | 294 | Yes | Morning | Serum |
| Alfaro-Magallanes et al.* (103) b | Low | 16 | f | 25.3 | 21.34 | 47.4 | Running | Intermittent | 36 | Vigorous | 294 | Yes | Morning | Serum |
| Almada et al. (104) | Low | 5 | n/a | 20.6 | 23.9 | 44.9 | Cycling | Continuous | 45 | Moderate | 243 | Yes | Morning | Plasma |
| Alves Monteiro et al. (105) a | Moderate | 10 | m | 26.9 | n/a | 53.6 | Running | Intermittent | n/a | Maximal | n/a | n/a | Morning | n/a |
| Alves Monteiro et al. (105) b | Moderate | 10 | m | 26.9 | n/a | 54.52 | Running | Intermittent | n/a | Maximal | n/a | n/a | Morning | n/a |
| Antunes et al. (106) a | Moderate | 19 | m | 30 | 24.2 | 52.6 | Cycling | Continuous | 60 | Maximal | 528 | No | Morning | Plasma |
| Antunes et al. (106) b | Moderate | 19 | m | 30 | 24.2 | 52.6 | Cycling | Continuous | 60 | Maximal | 528 | No | Morning | Plasma |
| Antunes et al. (106) c | Moderate | 19 | m | 30 | 24.2 | 52.6 | Cycling | Continuous | 60 | Maximal | 528 | No | Morning | Plasma |
| Araujo et al. (107) | Serious | 30 | m | 25 | n/a | n/a | Running | Continuous | 90 | n/a | n/a | n/a | Morning | n/a |
| Arroyo et al.* (108) | Moderate | 12 | m | 24.4 | 25.15 | 47.2 | Cycling | Continuous | 60 | Moderate | 323 | Yes | Morning | Plasma |
| Bacurau et al. (109) | Low | 12 | m | 28 | n/a | 61.85 | Cycling | Intermittent | 240 | Vigorous | 1764 | No | Morning | Plasma |
| Barba-Moreno et al.* (110) a | Moderate | 15 | f | 35.6 | 21.87 | 50.3 | Running | Continuous | 40 | Vigorous | 286 | Yes | Morning | Serum |
| Barba-Moreno et al.* (110) b | Moderate | 15 | f | 35.6 | 21.87 | 50.3 | Running | Continuous | 40 | Vigorous | 286 | Yes | Morning | Serum |
| Barba-Moreno et al.* (110) c | Moderate | 15 | f | 35.6 | 21.87 | 50.3 | Running | Continuous | 40 | Vigorous | 286 | Yes | Morning | Serum |
| Blegen et al. (111) a | Serious | 9 | m | 27 | 26.42 | 52.01 | Cycling | Continuous | 60 | Light | 144 | n/a | n/a | Plasma |
| Blegen et al. (111) b | Serious | 9 | m | 27 | 26.42 | 52.01 | Cycling | Continuous | 60 | Moderate | 323 | n/a | n/a | Plasma |
| Brenner et al.* (112) | Serious | 8 | m | 24.9 | 24.81 | 43 | Cycling | Continuous | 120 | Moderate | 647 | n/a | n/a | Plasma |
| Broadbent et al. (113) a | Critical | 14 | m | 33 | 24.26 | 57.4 | Running | Continuous | 40 | n/a | n/a | n/a | n/a | Plasma |
| Broadbent et al. (113) b | Critical | 15 | m | 33.1 | 23.36 | 57.1 | Running | Continuous | 40 | n/a | n/a | n/a | n/a | Plasma |
| Bruunsgaard et al. (114) a | Moderate | 9 | m | 26 | n/a | 51.1 | Running | Continuous | 30 | Vigorous | 193 | Yes | Morning | n/a |
| Bruunsgaard et al. (114) b | Moderate | 9 | m | 26 | n/a | 51.1 | Running | Continuous | 30 | Vigorous | 193 | Yes | Morning | n/a |
| Cabral-Santos et al.* (115) a | Moderate | 8 | m | 24.56 | 24.28 | 59.93 | Running | Intermittent | 30.78 | Maximal | 271 | n/a | n/a | Serum |
| Cabral-Santos et al.* (115) b | Moderate | 8 | m | 24.56 | 24.28 | 59.93 | Running | Continuous | 42.09 | Vigorous | 279 | n/a | n/a | Serum |
| Chaffin et al.* (116) a | Serious | 9 | f | 26.8 | 20.76 | 49.7 | Running | Intermittent | 75 | Vigorous | 551 | n/a | n/a | Plasma |
| Chaffin et al.* (116) b | Serious | 9 | f | 26.8 | 20.76 | 49.7 | Running | Intermittent | 75 | Vigorous | 551 | n/a | n/a | Plasma |
| Chan et al.* (117) | Serious | 8 | m | 24 | 23.5 | 49 | Cycling | Continuous | 60 | Vigorous | 397 | Yes | Morning | Plasma |
| Cho et al.* (118) a | Serious | 10 | m | 21.6 | 23.83 | 48.48 | Running | Continuous | n/a | Vigorous | n/a | n/a | n/a | Serum |
| Cho et al.* (118) b | Serious | 10 | m | 20.7 | 23.6 | 48.94 | Running | Continuous | n/a | Vigorous | n/a | n/a | n/a | Serum |
| Christiansen et al. (119) | Moderate | 15 | mf | 32.7 | 22.4 | 52.2 | Cycling | Continuous | 120 | Light | 240 | Yes | Morning | Serum |
| Cipryan et al.* (120) a | Moderate | 8 | m | 22.1 | 21.85 | 66.2 | Running | Intermittent | 24 | Maximal | 211 | Yes | Morning | Serum |
| Cipryan et al.* (120) b | Moderate | 8 | m | 22.9 | 23.55 | 56.8 | Running | Intermittent | 24 | Maximal | 211 | Yes | Morning | Serum |
| Cipryan et al.* (120) c | Moderate | 8 | m | 22.1 | 21.85 | 66.2 | Running | Intermittent | 21 | Maximal | 185 | Yes | Morning | Serum |
| Cipryan et al.* (120) d | Moderate | 8 | m | 22.9 | 23.55 | 56.8 | Running | Intermittent | 21 | Maximal | 185 | Yes | Morning | Serum |
| Cipryan et al.* (120) e | Moderate | 8 | m | 22.1 | 21.85 | 66.2 | Running | Continuous | 32 | Moderate | 118 | Yes | Morning | Serum |
| Cipryan et al.* (120) f | Moderate | 8 | m | 22.9 | 23.55 | 56.8 | Running | Continuous | 32 | Moderate | 118 | Yes | Morning | Serum |
| Cipryan (121) a | Moderate | 11 | n/a | 24.18 | 23.54 | 61.39 | Running | Intermittent | 24 | Maximal | 211 | Yes | Morning | Serum |
| Cipryan (121) b | Moderate | 10 | n/a | 22.6 | 24.73 | 53.46 | Running | Intermittent | 24 | Maximal | 211 | Yes | Morning | Serum |
| Cipryan (121) c | Moderate | 9 | n/a | 24.44 | 24.89 | 47.21 | Running | Intermittent | 24 | Maximal | 211 | Yes | Morning | Serum |
| Collins et al.* (122) | Serious | 13 | m | 37 | 31.6 | 34.3 | Cycling | Intermittent | 30 | Maximal | 264 | n/a | Morning | Serum |
| Cosio-Lima et al. (123) | Moderate | 6 | m | 27 | 23.9 | 66 | Cycling | Continuous | 150 | Vigorous | 1071 | No | Afternoon | Plasma |
| Costa et al. (124) a | Moderate | 11 | m | 34 | 24.09 | 59 | Running | Continuous | 120 | Vigorous | 794 | n/a | Morning | Plasma |
| Costa et al. (124) b | Moderate | 11 | m | 34 | 24.09 | 59 | Running | Continuous | 120 | Vigorous | 794 | n/a | Morning | Plasma |
| Cox et al. (125) a | Moderate | 10 | m | 33.7 | 22.13 | 64 | Running | Continuous | 30 | Vigorous | 183 | No | Morning | Plasma |
| Cox et al. (125) b | Moderate | 10 | m | 33.7 | 22.13 | 64 | Running | Continuous | 60 | Vigorous | 366 | No | Morning | Plasma |
| Cox et al. (125) c | Moderate | 10 | m | 33.7 | 22.13 | 64 | n/a | Intermittent | 27 | Maximal | 238 | No | Morning | Plasma |
| Cox et al. (125) d | Moderate | 8 | m | 28.1 | 22.28 | 64 | Running | Continuous | 30 | Vigorous | 183 | No | Morning | Plasma |
| Cox et al. (125) e | Moderate | 8 | m | 28.1 | 22.28 | 64 | Running | Continuous | 60 | Vigorous | 366 | No | Morning | Plasma |
| Cox et al. (125) f | Moderate | 8 | m | 28.1 | 22.28 | 64 | n/a | Intermittent | 27 | Maximal | 238 | No | Morning | Plasma |
| Croft et al.* (126) a | Moderate | 5 | m | 20 | 25.25 | n/a | Running | Intermittent | 30 | Maximal | 264 | n/a | n/a | Plasma |
| Croft et al.* (126) b | Moderate | 5 | m | 21 | 23.04 | n/a | Running | Intermittent | 30 | Maximal | 264 | n/a | n/a | Plasma |
| Croft et al.* (126) c | Moderate | 5 | m | 20 | 23.46 | n/a | Running | Intermittent | 30 | Maximal | 264 | n/a | n/a | Plasma |
| Croft et al.* (126) d | Moderate | 5 | m | 20 | 25.25 | n/a | Running | Intermittent | 30 | Maximal | 264 | n/a | n/a | Plasma |
| Croft et al.* (126) e | Moderate | 5 | m | 21 | 23.04 | n/a | Running | Intermittent | 30 | Maximal | 264 | n/a | n/a | Plasma |
| Croft et al.* (126) f | Moderate | 5 | m | 20 | 23.46 | n/a | Running | Intermittent | 30 | Maximal | 264 | n/a | n/a | Plasma |
| Cullen et al. (127) a | Moderate | 10 | mf | 24 | 23.18 | 49 | Cycling | Continuous | 35 | Moderate | 129 | No | n/a | Plasma |
| Cullen et al. (127) b | Moderate | 10 | mf | 24 | 23.18 | 49 | Cycling | Intermittent | 35 | Vigorous | 268 | No | n/a | Plasma |
| Cullen et al. (127) c | Moderate | 10 | mf | 24 | 23.18 | 49 | Cycling | Intermittent | 35 | Vigorous | 268 | No | n/a | Plasma |
| Degerstrøm & Østerud* (128) | Moderate | 8 | n/a | 23.4 | n/a | 66 | Running | Continuous | 30 | Vigorous | 230 | No | Morning | Plasma |
| Dorneles et al.* (129) a | Low | 10 | m | 26.5 | 22 | n/a | Running | Intermittent | 22.5 | Vigorous | 165 | No | Morning | Serum |
| Dorneles et al.* (129) b | Low | 10 | m | 26.5 | 22 | n/a | Running | Intermittent | 22.5 | Vigorous | 165 | No | Morning | Serum |
| dos Santos et al. (130) a | Moderate | 9 | m | 25.75 | 23.83 | 51.33 | Running | Intermittent | n/a | Maximal | n/a | Yes | Morning | Serum |
| dos Santos et al. (130) b | Moderate | 9 | m | 25.75 | 23.83 | 51.33 | Running | Intermittent | n/a | Maximal | n/a | Yes | Morning | Serum |
| Dufaux et al.* (131) | Critical | 8 | m | 24 | n/a | n/a | Running | Continuous | 150 | n/a | n/a | n/a | n/a | Plasma |
| Edwards et al.* (132) a | Moderate | 24 | mf | 24.2 | 24.1 | n/a | Cycling | Continuous | 45 | Maximal | 396 | No | Afternoon | Plasma |
| Edwards et al.* (132) b | Moderate | 24 | mf | 24.2 | 24.1 | n/a | Cycling | Continuous | 45 | Vigorous | 331 | No | Afternoon | Plasma |
| Ely et al.* (133) | Moderate | 12 | mf | 23 | 21.67 | 47.6 | Running | Continuous | 45 | Moderate | 200 | Yes | Morning | n/a |
| Febbraio et al. (134) | Low | 6 | m | 20.8 | 24.66 | n/a | Cycling | Continuous | 115 | Vigorous | 702 | Yes | Morning | Plasma |
| Ferguson et al. (135) a | Low | 9 | m | 21 | 24 | 54 | Running | Intermittent | 90 | Vigorous | 662 | Yes | Morning | Plasma |
| Ferguson et al. (135) b | Low | 9 | m | 21 | 24 | 54 | Running | Intermittent | 90 | Vigorous | 662 | Yes | Morning | Plasma |
| Ferguson et al. (135) c | Low | 9 | m | 21 | 24 | 54 | Running | Intermittent | 90 | Vigorous | 662 | Yes | Morning | Plasma |
| Fonseca et al. (136) a | Low | 12 | m | 22.5 | 23.21 | 45.5 | Cycling | Continuous | 45 | Vigorous | 321 | n/a | n/a | Plasma |
| Fonseca et al. (136) b | Low | 12 | m | 22.5 | 23.21 | 45.5 | Cycling | Continuous | 45 | Vigorous | 321 | n/a | n/a | Plasma |
| Fonseca et al. (136) c | Low | 12 | m | 22.5 | 23.21 | 45.5 | Cycling | Continuous | 60 | Vigorous | 428 | n/a | n/a | Plasma |
| Gagnon et al. (137) a | Moderate | 9 | m | 24 | 25.34 | 52.9 | Cycling | Continuous | 60 | Moderate | 221 | n/a | Morning | Serum |
| Gagnon et al. (137) b | Moderate | 9 | m | 24 | 25.34 | 52.9 | Cycling | Continuous | 60 | Vigorous | 397 | n/a | Morning | Serum |
| García et al. (138) | Critical | 15 | f | 22 | n/a | n/a | Cycling | Continuous | 60 | Vigorous | 397 | No | Morning | Serum |
| Garneau et al.* (139) | Moderate | 5 | f | 28.8 | 27.38 | n/a | Cycling | Continuous | 60 | Vigorous | 397 | Yes | Morning | Plasma |
| Ghafourin et al. (140) a | Critical | 20 | m | 21.3 | 22.87 | 53 | Running | Continuous | 30 | Vigorous | 183 | n/a | n/a | Serum |
| Ghafourin et al. (140) b | Critical | 20 | m | 21.3 | 22.87 | 53 | Running | Intermittent | 27 | Vigorous | 221 | n/a | n/a | Serum |
| Gill et al. (141) | Critical | 17 | mf | 40 | 24.93 | n/a | Running | Continuous | 1440 | Moderate | 4968 | No | n/a | Plasma |
| Giraldo et al. (142) a | Critical | 15 | f | 22 | n/a | n/a | n/a | Continuous | 60 | Vigorous | 397 | Yes | Morning | Serum |
| Giraldo et al. (142) b | Critical | 15 | f | 22 | n/a | n/a | n/a | Continuous | 45 | Moderate | 272 | Yes | Morning | Serum |
| Górecka et al.* (143) | Critical | 11 | m | 33.7 | 24 | 54.8 | Running | Continuous | 246 | Moderate | 1095 | n/a | n/a | Serum |
| Goto et al. (144) | Moderate | 9 | m | 19.8 | 19.99 | 63.4 | Running | Intermittent | 79 | Maximal | 695 | Yes | n/a | Plasma |
| Goto et al.* (145) | Moderate | 10 | m | 23 | 23.66 | n/a | Cycling | Continuous | 60 | Vigorous | 366 | Yes | Morning | Plasma |
| Gough et al.* (146) a | Serious | 11 | f | 33 | 22.5 | 49 | Running | Continuous | 30 | Moderate | 134 | Yes | Morning | Plasma |
| Gough et al.* (146) b | Serious | 11 | f | 33 | 22.5 | 49 | Running | Continuous | 30 | Vigorous | 221 | Yes | Morning | Plasma |
| Gough et al.* (146) c | Serious | 11 | f | 33 | 22.5 | 49 | Running | Continuous | 30 | Vigorous | 221 | Yes | Morning | Plasma |
| Gray et al. (147) | Moderate | 9 | m | 25.8 | 25.1 | 52 | Cycling | Continuous | 60 | Moderate | 267 | Yes | Morning | Plasma |
| Gusba et al. (148) a | Low | 10 | m | 22.6 | 24.01 | 44 | Cycling | Continuous | 117 | Vigorous | 714 | No | Morning | Plasma |
| Gusba et al. (148) b | low | 10 | m | 22.6 | 24.01 | 44 | Cycling | Continuous | 117 | Vigorous | 714 | No | Morning | Plasma |
| Hacker et al. (149) a | Moderate | 49 | mf | 25.61 | 23.7 | 45.9 | Running | Continuous | 40 | Vigorous | 294 | n/a | Morning | Plasma |
| Hacker et al. (149) b | Moderate | 49 | mf | 25.61 | 23.7 | 45.9 | Running | Continuous | 40 | Vigorous | 294 | n/a | Morning | Plasma |
| Hackney et al. (150) a | Critical | 8 | f | 25 | 21.12 | 57.5 | Running | Continuous | 90 | Vigorous | 596 | n/a | n/a | n/a |
| Hackney et al. (150) b | Critical | 8 | f | 25 | 21.12 | 57.5 | Running | Continuous | 90 | Vigorous | 596 | n/a | n/a | n/a |
| Harris et al. (151) | Critical | 12 | f | 30.1 | 26.1 | 33 | Cycling | Continuous | 55 | Moderate | 245 | n/a | n/a | Serum |
| He et al. (152) a | Moderate | 17 | m | 23 | 22 | 49 | Running | Intermittent | 28 | Vigorous | 200 | Yes | Morning | Serum |
| He et al. (153) b | Moderate | 17 | m | 23 | 22 | 49 | Running | Intermittent | 32 | Vigorous | 212 | Yes | Morning | Serum |
| He et al. (154) a | Moderate | 14 | m | 23 | 22 | 46 | Running | Continuous | 45 | n/a | n/a | Yes | Morning | Serum |
| He et al. (154) b | Moderate | 14 | m | 23 | 22 | 46 | Running | Continuous | 45 | Vigorous | 321 | Yes | Morning | Serum |
| Hojman et al.* (155) | Critical | 13 | m | 26 | 23.5 | n/a | Cycling | Intermittent | 120 | Vigorous | 882 | n/a | n/a | Plasma |
| Islam et al. (156) a | Low | 8 | m | 23.1 | 24.8 | 51.2 | Running | Continuous | 30 | Vigorous | 183 | Yes | Morning | Plasma |
| Islam et al. (156) b | Low | 8 | m | 23.1 | 24.8 | 51.2 | Running | Continuous | 30 | Vigorous | 245 | Yes | Morning | Plasma |
| Islam et al.* (157) a | Low | 16 | mf | 29.9 | 22.8 | 51 | Cycling | Continuous | 38 | Moderate | 169 | Yes | Morning | Plasma |
| Islam et al.* (157) b | Low | 16 | mf | 29.9 | 22.8 | 51 | Cycling | Continuous | 28 | Vigorous | 206 | Yes | Morning | Plasma |
| Islam et al.* (157) c | Low | 16 | mf | 29.9 | 22.8 | 51 | Cycling | Intermittent | 28 | Vigorous | 206 | Yes | Morning | Plasma |
| Jimenez et al. (158) | Moderate | 8 | n/a | 23 | 23.39 | 52 | Running | Continuous | 120 | Vigorous | 732 | No | Morning | n/a |
| Joisten et al.* (159) | Low | 24 | m | 24.6 | 25.4 | 48.3 | Cycling | Continuous | 45 | Moderate | 200 | No | n/a | Serum |
| Jürimäe et al. (160) | Moderate | 9 | m | 20.1 | 23.98 | n/a | n/a | Continuous | 120 | Vigorous | 882 | No | n/a | Plasma |
| Kakanis et al. (161) | Low | 10 | m | 24.2 | 22.28 | 65.9 | Cycling | Continuous | 120 | n/a | n/a | Yes | Morning | Plasma |
| Kastelein et al. (162) a | Low | 14 | m | 22 | 24.69 | 36.67 | Cycling | Continuous | 40 | Moderate | 147 | Yes | Morning | Serum |
| Kastelein et al. (162) b | Low | 14 | m | 22 | 26.23 | 39.27 | Cycling | Continuous | 40 | Moderate | 147 | Yes | Morning | Serum |
| Kastelein et al (162) c | Low | 13 | m | 33 | 25.92 | 33.93 | Cycling | Continuous | 40 | Moderate | 147 | Yes | Morning | Serum |
| Kastelein et al (162) d | Low | 13 | m | 36 | 28.67 | 31.62 | Cycling | Continuous | 40 | Moderate | 147 | Yes | Morning | Serum |
| Kastelein et al.* (163) a | Low | 11 | m | 24 | 27.91 | 36.4 | Cycling | Continuous | 40 | Moderate | 147 | No | Morning | Plasma |
| Kastelein et al.* (163) b | Low | 11 | m | 23.2 | 25.32 | 36.9 | Cycling | Continuous | 40 | Moderate | 147 | No | Morning | Plasma |
| Kastelein et al.* (163) c | Low | 11 | m | 23.2 | 25.32 | 36.9 | Cycling | Continuous | 40 | Moderate | 147 | No | Morning | Plasma |
| Kim et al. (164) a | Low | 14 | m | 24.3 | 23.4 | 48.7 | Running | Continuous | 60 | Moderate | 323 | No | Morning | Plasma |
| Kim et al. (164) b | Low | 14 | m | 24.3 | 23.4 | 48.7 | Running | Continuous | 60 | Moderate | 323 | No | Afternoon | Plasma |
| Kon & Tanimura (165) | Low | 8 | m | 20.4 | 22.7 | 51.3 | Cycling | Continuous | 30 | Vigorous | 214 | Yes | Morning | Serum |
| Krzemiński et al. (166) | Critical | 9 | m | 30 | 22.6 | 54.1 | Running | Continuous | 1020 | Moderate | 4539 | n/a | n/a | Plasma |
| Kuhne et al. (167) | Critical | 27 | mf | 26.9 | 23.88 | 33.6 | n/a | Continuous | 50 | n/a | n/a | n/a | n/a | Serum |
| Landers-Ramos et al.* (168) | Critical | 8 | mf | 40 | 24 | 51.4 | Running | Continuous | n/a | n/a | n/a | No | Morning | Plasma |
| Larsen et al. (169) a | Critical | 8 | f | 22 | 22.3 | 44.8 | Cycling | Continuous | 52.5 | Maximal | 462 | Yes | Morning | Serum |
| Larsen et al. (169) b | Critical | 8 | f | 22 | 22.2 | 44.5 | Cycling | Continuous | 52.5 | Maximal | 462 | Yes | Morning | Serum |
| LaVoy et al. (170) | Serious | 12 | m | 36.7 | 24.36 | 54.1 | Cycling | Continuous | 156 | Moderate | 694 | No | Afternoon | Plasma |
| Leggate et al.* (171) a | Moderate | 11 | m | 22.3 | 23 | 51 | Cycling | Intermittent | 40 | Vigorous | 327 | Yes | Morning | Plasma |
| Leggate et al.* (171) b | Moderate | 11 | m | 22.3 | 23 | 51 | Cycling | Continuous | 40 | Moderate | 216 | Yes | Morning | Plasma |
| Li & Gleeson* (172) a | Moderate | 8 | m | 28.9 | n/a | 56.1 | Cycling | Continuous | 120 | Moderate | 647 | Yes | Morning | Plasma |
| Li & Gleeson* (172) b | Moderate | 8 | m | 28.9 | n/a | 56.1 | Cycling | Continuous | 120 | Moderate | 647 | Yes | Afternoon | Plasma |
| Lira et al. (173) a | Serious | 10 | m | 26.36 | 23.74 | 52.82 | Running | Intermittent | n/a | n/a | n/a | Yes | Morning | n/a |
| Lira et al. (173) b | Serious | 10 | m | 26.36 | 23.74 | 52.82 | Running | Continuous | n/a | n/a | n/a | Yes | Morning | n/a |
| Lira et al. (173) c | Serious | 10 | m | 26.36 | 23.74 | 52.82 | Running | Intermittent | n/a | n/a | n/a | Yes | Morning | n/a |
| Lira et al. (173) d | Serious | 10 | m | 26.36 | 23.74 | 52.82 | Running | Continuous | n/a | n/a | n/a | Yes | Morning | n/a |
| Lobo et al. (174) a | Moderate | 15 | m | 30.5 | n/a | 54.2 | Running | Continuous | n/a | Vigorous | n/a | n/a | n/a | Plasma |
| Lobo et al. (174) b | Moderate | 18 | f | 34.2 | n/a | 44 | Running | Continuous | n/a | Vigorous | n/a | n/a | n/a | Plasma |
| MacNeil et al.* (175) a | Critical | 15 | mf | 29.6 | 24.56 | 33.8 | Cycling | Continuous | 30 | Moderate | 134 | Yes | n/a | Plasma |
| MacNeil et al.* (175) b | Critical | 16 | mf | 31.5 | 22.63 | 57.3 | Cycling | Continuous | 30 | Moderate | 134 | Yes | n/a | Plasma |
| Maharaj et al.* (176) | Low | 11 | mf | 23.727 | 22.08 | 46.66 | Running | Continuous | 30 | Vigorous | 214 | Yes | Morning | Plasma |
| Marcucci‑Barbosa et al.* (177) | Moderate | 9 | m | 21.21 | 25.9 | n/a | Running | Continuous | 49.8 | n/a | n/a | n/a | Morning | Plasma |
| Marklund et al. (178) | Moderate | 9 | m | 28.1 | 24.2 | 62.12 | Cycling | Continuous | 1440 | Moderate | 6408 | Yes | n/a | Plasma |
| Markus et al. (179) a | Serious | 14 | m | 26.1 | 24.1 | 50.4 | Running | Continuous | 60 | Moderate | 267 | No | n/a | Plasma |
| Markus et al. (179) b | Serious | 14 | m | 43.6 | 25.3 | 46.5 | Running | Continuous | 60 | Moderate | 267 | No | n/a | Plasma |
| McCormick et al. (180) a | Moderate | 16 | mf | 27.8 | 21.11 | 59.2 | Running | Continuous | 90 | Vigorous | 549 | Yes | Morning | Serum |
| McCormick et al. (180) b | Moderate | 16 | mf | 27.8 | 21.11 | 59.2 | Running | Continuous | 90 | Vigorous | 549 | Yes | Afternoon | Serum |
| McKay et al. (181) | Serious | 12 | m | 26.6 | 22.73 | 63 | Running | Continuous | 60 | Vigorous | 441 | Yes | Morning | Serum |
| McKenzie et al. (182) a | Moderate | 10 | f | 23.1 | 26.08 | 35.82 | Running | Continuous | 30 | Vigorous | 199 | Yes | Morning | n/a |
| McKenzie et al. (182) b | Moderate | 10 | f | 24.8 | 24.73 | 39.98 | Running | Continuous | 30 | Vigorous | 199 | Yes | Morning | n/a |
| Mendham et al.* (183) | Moderate | 10 | m | 38.5 | 31.9 | n/a | Cycling | Continuous | 40 | Vigorous | 256 | Yes | Morning | Plasma |
| Mills et al. (184) a | Low | 6 | m | 27 | 24.42 | 52 | Cycling | Continuous | 60 | n/a | n/a | n/a | n/a | Plasma |
| Mills et al. (184) b | Low | 6 | m | 27 | 24.42 | 52 | Cycling | Continuous | 60 | n/a | n/a | n/a | n/a | Plasma |
| Mills et al. (184) c | Low | 6 | m | 22 | 23.62 | 47 | Cycling | Continuous | 60 | n/a | n/a | n/a | n/a | Plasma |
| Mills et al. (184) d | Low | 6 | m | 22 | 23.62 | 47 | Cycling | Continuous | 60 | n/a | n/a | n/a | n/a | Plasma |
| Minuzzi et al.* (185) a | Low | 14 | f | 24 | 22.8 | 41.5 | Running | Intermittent | 20 | Vigorous | 147 | n/a | Morning | Serum |
| Minuzzi et al.* (185) b | Low | 14 | f | 24 | 22.8 | 41.5 | Running | Intermittent | 20 | Vigorous | 147 | n/a | Morning | Serum |
| Moldoveanu et al. (186) | Serious | 10 | m | 25 | n/a | 48.8 | Cycling | Intermittent | 60 | Moderate | 267 | Yes | n/a | Plasma |
| Neidhart et al. (187) | Critical | 8 | m | 25-34 | n/a | n/a | Running | Continuous | n/a | n/a | n/a | n/a | n/a | Serum |
| Nelson et al. (188) | Moderate | 14 | mf | 28.6 | 22.1 | 73.3 | Running | Continuous | 90 | Moderate | 485 | Yes | Morning | Plasma |
| Newlin et al. (189) a | Low | 12 | f | 23.8 | 21.61 | 52.1 | Running | Continuous | 60 | Vigorous | 366 | n/a | n/a | Serum |
| Newlin et al. (189) b | Low | 12 | f | 23.8 | 21.61 | 52.1 | Running | Continuous | 120 | Vigorous | 732 | n/a | n/a | Serum |
| Nieman et al. (190) | Serious | 31 | m | 38.8 | 24.66 | 55.8 | Cycling | Continuous | 105 | Vigorous | 772 | No | afternoon | Plasma |
| Nieman et al. (191) | Moderate | 19 | m | 38 | 23.44 | 51.7 | Cycling | Continuous | 162 | Vigorous | 1191 | Yes | Morning | Plasma |
| Nieman et al. (192) | Serious | 24 | m | 36.5 | 24.39 | 60 | Running | Continuous | 120 | Vigorous | 794 | No | Afternoon | Plasma |
| Nieman et al. (193) a | Serious | 10 | m | 36.3 | 23.51 | 57.2 | Running | Continuous | 120 | Vigorous | 794 | Yes | Morning | Plasma |
| Nieman et al. (193) b | Serious | 10 | f | 35.4 | 21 | 45.2 | Cycling | Continuous | 120 | Vigorous | 794 | Yes | Morning | Plasma |
| Nieman et al.* (194) | Critical | 20 | m | 38.4 | 25.15 | 47.9 | Cycling | Continuous | 168 | Vigorous | 1235 | n/a | n/a | Plasma |
| Niess et al. (195) | Critical | 12 | mf | 26.6 | 20.14 | 64.6 | Running | Intermittent | n/a | Vigorous | n/a | n/a | Morning | Plasma |
| Niess et al.* (196) | Serious | 7 | m | 29.7 | 22.87 | 66.3 | Running | Continuous | 60 | Vigorous | 441 | n/a | Morning | Plasma |
| Osborne et al. (197) | Low | 8 | m | 28 | 22.94 | 58 | Cycling | Intermittent | 60 | Moderate | 267 | n/a |  | Serum |
| Ostrowski et al.* (198) | Moderate | 10 | m | 38 | n/a | 59.7 | Running | Continuous | 150 | Vigorous | 1071 | Yes | Morning | Plasma |
| Pasqua et al. (199) | Critical | 10 | m | 25.9 | 24 | 42.2 | Cycling | Continuous | 90 | n/a | n/a | n/a | n/a | Serum |
| Pawłowska et al.* (200) a | Critical | 23 | m | 25 | 25.3 | 40.8 | Cycling | Continuous | 30 | Moderate | 134 | n/a | n/a | Serum |
| Pawłowska et al.* (200) b | Critical | 23 | m | 25 | 25.3 | 40.8 | Cycling | Continuous | 30 | Moderate | 134 | n/a | n/a | Serum |
| Pawłowska et al.* (200) c | Critical | 23 | m | 25 | 25.3 | 40.8 | Cycling | Continuous | 30 | Moderate | 134 | n/a | n/a | Serum |
| Pawłowska et al. (200) d | Critical | 23 | m | 25 | 25.3 | 40.8 | Cycling | Continuous | 30 | Moderate | 134 | n/a | n/a | Serum |
| Peake et al.* (201) | Serious | 10 | m | 28 | 23.67 | 61 | Running | Continuous | 45 | Moderate | 243 | n/a | Afternoon | Plasma |
| Peake et al. (202) a | Serious | 9 | m | 28 | 23.67 | 61 | Running | Continuous | 60 | Moderate | 323 | n/a | Afternoon | Plasma |
| Peake et al. (202) b | Serious | 9 | m | 28 | 23.67 | 61 | Running | Continuous | 60 | Vigorous | 491 | n/a | Afternoon | Plasma |
| Peake et al. (202) c | Serious | 9 | m | 28 | 23.67 | 61 | Running | Continuous | 45 | Moderate | 243 | n/a | Afternoon | Plasma |
| Peake et al. (203) | Critical | 10 | m | 27 | 23.78 | 61.61 | Cycling | Continuous | 90 | Moderate | 400 | n/a | Morning | Serum |
| Peake et al.* (204) a | Low | 10 | m | 33.2 | 23.4 | 61.07 | Cycling | Intermittent | 60 | Vigorous | 460 | n/a | Morning | Plasma |
| Peake et al.* (204) b | Low | 10 | m | 33.2 | 23.4 | 61.07 | Cycling | Continuous | 60 | Vigorous | 366 | n/a | Morning | Plasma |
| Peeling et al. (205) | Moderate | 11 | mf | 25.3 | 21.35 | 51.3 | Running | Continuous | 45 | Vigorous | 331 | Yes | Morning | Serum |
| Peeling et al.* (206) a | Critical | 10 | m | 28.3 | 22.85 | 61.5 | Running | Continuous | 46.5 | Vigorous | 308 | n/a | Afternoon | Serum |
| Peeling et al.* (206) b | Critical | 10 | m | 28.3 | 22.85 | 61.5 | Running | Intermittent | 38.7 | Vigorous | 337 | n/a | Morning | Serum |
| Peeling et al. (207) a | Moderate | 10 | m | 26.6 | 24.22 | 63.3 | Running | Intermittent | 36 | Vigorous | 294 | n/a | Morning | Serum |
| Peeling et al. (207) b | Moderate | 12 | mf | 28.8 | n/a | 58.2 | Running | Intermittent | 27.5 | Vigorous | 239 | n/a | Morning | Serum |
| Peeling et al. (207) c | Moderate | 12 | m | 23 | 22.23 | 60.12 | Running | Continuous | 90 | Vigorous | 643 | n/a | Morning | Serum |
| Peeling et al. (207) d | Moderate | 10 | f | 26 | 21.45 | 53 | Running | Continuous | 40 | Vigorous | 286 | n/a | Morning | Serum |
| Peeling et al. (207) e | Moderate | 10 | m | 23 | 23.25 | 66.35 | Running | Continuous | 40 | Vigorous | 244 | n/a | Morning | Serum |
| Peeling et al. (208) | Moderate | 24 | m | 27.2 | n/a | 64.9 | Running | Continuous | n/a | n/a | n/a | No | Afternoon | Serum |
| Pilat et al.* (209) | Serious | 40 | m | 25.5 | 24 | 46.9 | Cycling | Continuous | 60 | Vigorous | 460 | Yes | Morning | Serum |
| Panizo González et al. (210) | Critical | 88 | mf | 38.68 | 22.91 | n/a | Running | Continuous | n/a | n/a | n/a | n/a | n/a | n/a |
| Pistilli et al. (211) | Serious | 75 | m | 37.4 | 23.68 | 51.2 | Running | Continuous | 258.6 | Moderate | 1153 | Yes | Morning | Plasma |
| Pokora et al.* (212) a | Serious | 10 | m | 21.2 | 22.5 | n/a | Running | Continuous | 60 | Moderate | 267 | No | Morning | Plasma |
| Pokora et al.* (212) b | Serious | 10 | m | 21.4 | 21.9 | n/a | Running | Continuous | 60 | Moderate | 267 | No | Morning | Plasma |
| Pournot et al. (213) a | Critical | 11 | m | 31.8 | 22.03 | 62 | Running | Continuous | 48 | n/a | n/a | n/a | n/a | Plasma |
| Pournot et al. (213) b | Critical | 11 | m | 31.8 | 22.03 | 62 | Running | Continuous | 48 | n/a | n/a | n/a | n/a | Plasma |
| Pozzolo et al.* (214) a | Serious | 14 | n/a | 23.67 | 22.72 | n/a | Cycling | Continuous | 40 | Moderate | 178 | n/a | Morning | Plasma |
| Pozzolo et al.* (214) b | Serious | 14 | n/a | 23.67 | 22.72 | n/a | Cycling | Continuous | 40 | Moderate | 178 | n/a | Morning | Plasma |
| Proschinger et al.* (215) a | Low | 24 | mf | 29.7 | 22.2 | 56.6 | Running | Intermittent | 24.5 | Vigorous | 180 | Yes | n/a | Serum |
| Proschinger et al.* (215) b | Low | 24 | mf | 29.7 | 22.2 | 56.6 | Running | Continuous | 50 | Vigorous | 331 | Yes | n/a | Serum |
| Rämson et al. (216) a | Serious | 8 | m | 20.2 | 23.92 | n/a | n/a | Continuous | 120 | Vigorous | 882 | No | Afternoon | Plasma |
| Rämson et al. (216) b | Serious | 8 | m | 20.2 | 23.92 | n/a | n/a | Continuous | 120 | Vigorous | 882 | No | Afternoon | Plasma |
| Rämson et al. (216) c | Serious | 8 | m | 20.2 | 23.92 | n/a | n/a | Continuous | 120 | Vigorous | 882 | No | Afternoon | Plasma |
| Reichel et al.* (217) a | Moderate | 62 | mf | 25.3 | 23.4 | n/a | Running | Continuous | 40 | Maximal | 352 | No | Morning | Plasma |
| Reichel et al.* (217) b | Moderate | 62 | mf | 25.3 | 23.4 | n/a | Running | Continuous | 60 | Maximal | 352 | No | Morning | Plasma |
| Rinnov et al.* (218) | Serious | 8 | m | 27 | 23.3 | 47.4 | Cycling | Continuous | 180 | Moderate | 970 | Yes | Morning | Plasma |
| Robson-Ansley et al. (219) | Critical | 13 | m | 35 | n/a | n/a | Running | Continuous | 375 | Moderate | 1669 | Yes | n/a | Plasma |
| Rohde et al. (220) | Critical | 8 | m | 29.1 | n/a | n/a | n/a | Continuous | n/a | n/a | n/a | No | Morning | Plasma |
| Ronsen et al. (221) | Low | 9 | m | 24 | n/a | 69.1 | n/a | Continuous | 75 | Vigorous | 536 | Yes | Afternoon | Plasma |
| Said et al.* (222) a | Serious | 7 | m | 22.7 | 24.06 | n/a | Running | Continuous | n/a | Maximal | n/a | n/a | Morning | Serum |
| Said et al.* (222) b | Serious | 7 | m | 21 | 29.72 | n/a | Running | Continuous | n/a | Maximal | n/a | n/a | Morning | Serum |
| Said et al.* (222) c | Serious | 7 | m | 23.9 | 25.54 | n/a | Running | Continuous | n/a | Maximal | n/a | n/a | Morning | Serum |
| Sawai et al.* (223) a | Moderate | 11 | f | 24.8 | 20.89 | 45.3 | Cycling | Continuous | 30 | Moderate | 162 | Yes | Morning | Serum |
| Sawai et al.* (223) b | Moderate | 11 | f | 24.8 | 20.89 | 45.3 | Cycling | Continuous | 30 | Moderate | 162 | Yes | Morning | Serum |
| Sawai et al.* (223) c | Moderate | 11 | f | 24.8 | 20.89 | 45.3 | Cycling | Continuous | 30 | Moderate | 162 | Yes | Morning | Serum |
| Scharhag et al. (224) | Moderate | 12 | m | 26 | 22.16 | n/a | Cycling | Continuous | 240 | n/a | n/a | No | Morning | Plasma |
| Scott et al. (225) a | Low | 10 | m | 28 | 23.2 | 56.2 | Running | Continuous | 60 | Moderate | 267 | Yes | Morning | Plasma |
| Scott et al. (225) b | Low | 10 | m | 28 | 23.2 | 56.2 | Running | Continuous | 60 | Vigorous | 440 | Yes | Morning | Plasma |
| Scott et al. (225) c | Low | 10 | m | 28 | 23.2 | 56.2 | Running | Continuous | 60 | Vigorous | 440 | Yes | Morning | Plasma |
| Scott et al. (226) a | Low | 11 | m | 30 | 25 | 55 | Running | Continuous | 60 | Maximal | 528 | Yes | Morning | Plasma |
| Scott et al. (226) b | Low | 10 | m | 31 | 23.3 | 68 | Running | Continuous | 60 | Maximal | 528 | Yes | Morning | Plasma |
| Shojaei et al. (227) | Moderate | 10 | m | 21.01 | 22.56 | n/a | Cycling | Continuous | 45 | Moderate | 166 | n/a | Morning | n/a |
| Shojaei et al. (228) | Moderate | 10 | m | 21.03 | 20.8 | 59.6 | Cycling | Continuous | 45 | Moderate | 166 | n/a | Morning | n/a |
| Silva Acevedo et al. (229) | Moderate | 9 | mf | 28.67 | 25.1 | n/a | Running | Intermittent | 30 | Moderate | 134 | No | n/a | Plasma |
| Sim et al.* (230) a | Moderate | 10 | m | 23 | 23.25 | 66.35 | Running | Continuous | 40 | Vigorous | 244 | n/a | Morning | Serum |
| Sim et al.* (230) b | Moderate | 10 | m | 23 | 23.25 | 66.35 | Running | Continuous | 24 | Vigorous | 196 | n/a | Morning | Serum |
| Sim et al.* (230) c | Moderate | 10 | m | 23 | 23.25 | 66.35 | Cycling | Continuous | 40 | Vigorous | 244 | n/a | Morning | Serum |
| Sim et al.* (230) d | Moderate | 10 | m | 23 | 23.25 | 66.35 | Cycling | Intermittent | 24 | Vigorous | 196 | n/a | Morning | Serum |
| Sim et al.* (231) a | Moderate | 10 | f | 26 | 21.45 | 53 | Running | Continuous | 40 | Vigorous | 286 | n/a | Morning | Serum |
| Sim et al.* (231) b | Moderate | 10 | f | 26 | 21.45 | 53 | Running | Continuous | 40 | Vigorous | 286 | n/a | Morning | Serum |
| Simpson et al.* (232) | Critical | 7 | mf | 32 | 21.9 | 54 | Running | Continuous | 43 | Maximal | 378 | n/a | n/a | Plasma |
| Siqueira et al.* (233) | Critical | 20 | m | 38.61 | 23.95 | n/a | Running | Continuous | 42 | n/a | n/a | n/a | n/a | Plasma |
| Smith et al. (234) a | Serious | 6 | m | 19.9 | 24.08 | 47.1 | Running | Continuous | 60 | n/a | n/a | n/a | n/a | Serum |
| Smith et al. (234) b | Serious | 6 | m | 19.9 | 24.08 | 47.1 | Running | Continuous | 60 | n/a | n/a | n/a | n/a | Serum |
| Snipe et al. (235) | Critical | 10 | mf | 31 | n/a | 55.4 | Running | Continuous | 120 | Moderate | 647 | No | Morning | Plasma |
| Spanoudaki et al.* (236) a | Moderate | 10 | m | 24.7 | 23.9 | 47.9 | Cycling | Continuous | 60 | Moderate | 267 | Yes | Morning | n/a |
| Spanoudaki et al.* (236) b | Moderate | 10 | m | 24.7 | 23.9 | 47.9 | Cycling | Continuous | 60 | Moderate | 267 | Yes | Morning | n/a |
| Starkie et al.* (237) | Moderate | 6 | m | 25 | n/a | n/a | Cycling | Continuous | 120 | Vigorous | 794 | Yes | Morning | n/a |
| Starkie et al.* (238) | Critical | 5 | m | n/a | 23.22 | n/a | Running | Continuous | 169 | Moderate | 752 | n/a | n/a | n/a |
| Starkie et al.* (239) | Critical | 7 | m | 26 | n/a | 61.04 | Cycling | Continuous | 90 | Vigorous | 596 | n/a | n/a | Plasma |
| Starzak et al. (240) a | Moderate | 8 | m | 20 | 22.16 | n/a | Running | Continuous | 60 | Vigorous | 428 | Yes | Morning | Serum |
| Starzak et al. (240) b | Moderate | 7 | m | 22 | 23.39 | n/a | Running | Continuous | 60 | Vigorous | 428 | Yes | Morning | Serum |
| Steensberg et al.* (241) | Critical | 9 | m | n/a | n/a | 60.1 | Running | Continuous | 150 | Vigorous | 1071 | Yes | Morning | Plasma |
| Sugama et al.* (242) | Critical | 14 | m | 28.7 | n/a | n/a | n/a | Continuous | n/a | n/a | n/a | No | Afternoon | Serum |
| Sugama et al.* (243) | Critical | 14 | m | 28.7 | n/a | n/a | n/a | Continuous | n/a | n/a | n/a | No | Afternoon | Serum |
| Sumi et al.* (244) | Moderate | 9 | m | 19.7 | 19.99 | 63.9 | Running | Intermittent | 40 | Maximal | 352 | Yes | Morning | Plasma |
| Sureda et al. (245) | Moderate | 9 | m | 35 | 22.44 | 61.4 | Running | Continuous | 45 | Moderate | 266 | Yes | Morning | Plasma |
| Suzuki et al. (246) | Critical | 8 | m | 20 | 22.28 | 49.6 | Cycling | Continuous | 90 | Moderate | 400 | n/a | Morning | Plasma |
| Svendsen et al. (247) | low | 12 | m | 28 | n/a | 63.7 | Cycling | Continuous | 75 | Vigorous | 497 | Yes | Morning | Plasma |
| Tartibian et al.* (248) a | Moderate | 8 | m | 20.8 | 21.59 | 45.96 | Running | Continuous | 30 | Vigorous | 214 | Yes | n/a | Serum |
| Tartibian et al.* (248) b | Moderate | 10 | m | 21.5 | 21.34 | 45.35 | Running | Continuous | 30 | Moderate | 162 | Yes | n/a | Serum |
| Timmons et al. (249) a | Serious | 11 | m | 21 | 23.77 | 45 | Cycling | Continuous | 90 | Vigorous | 549 | Yes | Morning | Plasma |
| Timmons et al. (249) b | Serious | 6 | f | 21 | 22.84 | 37 | Cycling | Continuous | 90 | Vigorous | 549 | Yes | Morning | Plasma |
| Timmons et al. (249) c | Serious | 6 | f | 21 | 22.84 | 37 | Cycling | Continuous | 90 | Vigorous | 549 | Yes | Morning | Plasma |
| Timmons et al. (249) d | Serious | 6 | f | 22 | 22.66 | 40 | Cycling | Continuous | 90 | Vigorous | 549 | Yes | Morning | Plasma |
| Timmons et al. (249) e | Serious | 6 | f | 22 | 22.66 | 40 | Cycling | Continuous | 90 | Vigorous | 549 | Yes | Morning | Plasma |
| Timmons et al.* (250) a | Serious | 20 | f | 21 | n/a | n/a | Cycling | Continuous | 90 | Vigorous | 549 | n/a | n/a | Plasma |
| Timmons et al.* (250) b | Serious | 19 | f | 21 | n/a | n/a | Cycling | Continuous | 90 | Vigorous | 549 | n/a | n/a | Plasma |
| Tsuchiya & Goto (251) a | Moderate | 7 | m | 22.7 | 21.6 | 47 | Cycling | Continuous | 60 | Moderate | 323 | n/a | n/a | Plasma |
| Tsuchiya & Goto (251) b | Moderate | 7 | m | 22.7 | 21.6 | 49.7 | Cycling | Continuous | 60 | Moderate | 323 | n/a | n/a | Plasma |
| Ullum et al. (252) | Serious | 14 | m | 23-39 | n/a | 50.4 | Cycling | Continuous | 60 | Moderate | 428 | Yes | n/a | Plasma |
| Vaisberg et al. (253) | Critical | 14 | m | 38 | 23.7 | n/a | Running | Continuous | n/a | n/a | n/a | Yes | n/a | Serum |
| van de Vyver & Myburgh (254) | Critical | 6 | m | 22 | 23.2 | 52.4 | Running | Intermittent | 60 | Vigorous | 491 | n/a | n/a | Serum |
| van de Vyver & Myburgh* (255) a | Critical | 10 | m | 20.3 | 23.3 | 50.7 | Running | Intermittent | 60 | n/a | n/a | n/a | n/a | Serum |
| van de Vyver & Myburgh* (255) b | Critical | 5 | m | 20.90 | 23.8 | 50.7 | Running | Intermittent | 60 | n/a | n/a | n/a | n/a | Serum |
| van de Vyver & Myburgh* (255) c | Critical | 3 | m | 20.7 | 23.6 | 50.7 | Running | Intermittent | 60 | n/a | n/a | n/a | n/a | Serum |
| van de Vyver et al. (256) | Critical | 12 | m | 20.6 | 23.6 | 52 | Running | Intermittent | 60 | Moderate | 267 | n/a | n/a | Serum |
| Venhorst et al.* (257) | Critical | 22 | mf | 28.5 | 21.05 | 64 | Running | Continuous | 85 | n/a | n/a | n/a | n/a | Serum |
| Wadley et al.* (258) a | Low | 10 | m | 22 | 24 | 42.7 | Cycling | Continuous | 27 | Moderate | 146 | Yes | Morning | Plasma |
| Wadley et al.* (258) b | Low | 10 | m | 22 | 24 | 42.7 | Cycling | Continuous | 27 | Vigorous | 146 | Yes | Morning | Plasma |
| Wadley et al.* (259) a | Low | 9 | m | 29 | 24.2 | 44.5 | Cycling | Continuous | 58 | Moderate | 313 | Yes | Morning | Plasma |
| Wadley et al.* (259) b | Low | 9 | m | 29 | 24.2 | 44.5 | Cycling | Intermittent | 60 | Vigorous | 491 | Yes | Morning | Plasma |
| Wahl et al.* (260) | Moderate | 13 | n/a | 24.8 | 22.95 | 51.6 | Cycling | Continuous | 60 | Vigorous | 441 | No | n/a | Serum |
| Wallberg et al. (261) | Critical | 9 | m | 27 | 24.27 | 62.5 | Running | Continuous | 1440 | Moderate | 6408 | Yes | n/a | Plasma |
| Walshe et al. (262) a | Low | 9 | m | 27 | 22.1 | 59 | Running | Continuous | 120 | Vigorous | 882 | Yes | Morning | Plasma |
| Walshe et al. (262) b | Low | 9 | m | 27 | 22.1 | 59 | Running | Continuous | 120 | Vigorous | 882 | Yes | Morning | Plasma |
| Wang et al.* (263) | Critical | 5 | m | 24 | 23.51 | n/a | Running | Continuous | n/a | n/a | n/a | n/a | n/a | Serum |
| Woo et al.* (264) a | Critical | 6 | m | 23.67 | 24.88 | 47.17 | Running | Continuous | 60 | Vigorous | 360 | n/a | n/a | Serum |
| Woo et al.* (264) b | Critical | 6 | m | 21.67 | 24.12 | 49.15 | Running | Continuous | 60 | Vigorous | 360 | n/a | n/a | Serum |
| Zaldivar et al. (265) | Critical | 11 | m | 24.8 | 24.8 | 37.1 | Cycling | Continuous | 30 | Vigorous | 230 | n/a | n/a | Serum |
| Zheng et al.* (266) a | Low | 8 | f | 37 | 22.86 | 46 | Cycling | Continuous | 30 | Vigorous | 221 | n/a | Morning | Serum |
| Zheng et al.* (266) b | Low | 8 | f | 37 | 22.86 | 46 | Cycling | Continuous | 30 | Vigorous | 221 | n/a | Morning | Serum |

**Note** Black fond: studies included in meta-analysis, Light grey fond: studies included only in systematic review. *myokine concentration extracted via Web plot digitizer. *f* female, *m* male, *min* minute, *mf* mixed, *n/a* not applicable.

# Table S3 Risk assessment

| *Study* | *Risk of bias due to confounding* | *Risk of bias in selection of participants* | *Risk of bias due to devi-ations from intended interventions* | *Risk of bias due to missing data* | *Risk of bias arising from measure-ment of the outcome* | *Risk of bias in selection of the reported result* | *TOTAL* |
| --- | --- | --- | --- | --- | --- | --- | --- |
| Acevedo et al. (100) | low | low | low | low | low | low | **low** |
| Akerstrom et al. (101) | moderate | low | low | low | low | low | **moderate** |
| Aktitiz et al. (102) | moderate | low | low | low | low | low | **moderate** |
| Alfaro-Magallanes et al. (103) | low | low | low | low | low | low | **low** |
| Almada et al. (104) | low | low | low | low | low | low | **low** |
| Alves Monteiro et al. (105) | moderate | low | low | low | low | low | **moderate** |
| Antunes et al. (106) | moderate | low | low | low | low | low | **moderate** |
| Araujo et al. (107) | serious | low | low | low | low | low | **serious** |
| Arroyo et al. (108) | moderate | low | low | low | low | low | **moderate** |
| Bacurau et al. (109) | low | low | low | low | low | low | **low** |
| Barba-Moreno et al. (110) | low | low | low | low | moderate | low | **moderate** |
| Blegen et al. (111) | serious | low | low | low | low | low | **serious** |
| Brenner et al. (112) | serious | low | low | low | low | low | **serious** |
| Broadbent et al. (113) | critical | low | low | low | low | low | **critical** |
| Bruunsgaard et al. (114) | moderate | low | low | low | low | low | **moderate** |
| Cabral-Santos et al. (115) | moderate | low | low | low | low | low | **moderate** |
| Chaffin et al. (116) | serious | low | low | low | low | low | **serious** |
| Chan et al. (117) | serious | low | low | low | low | low | **serious** |
| Cho et al. (118) | serious | low | low | low | low | moderate | **serious** |
| Christiansen et al. (119) | moderate | low | low | low | low | low | **moderate** |
| Cipryan et al. (120) | moderate | low | low | moderate | low | low | **moderate** |
| Cipryan (121) | moderate | low | low | moderate | low | low | **moderate** |
| Collins et al. (122) | **serious** | low | low | low | low | low | **serious** |
| Cosio-Lima et al. (123) | moderate | low | low | low | low | low | **moderate** |
| Costa et al. (124) | moderate | low | low | low | low | low | **moderate** |
| Cox et al. (125) | moderate | low | low | low | low | low | **moderate** |
| Croft et al. (126) | moderate | low | low | low | low | low | **moderate** |
| Cullen et al. (127) | moderate | low | low | low | low | low | **moderate** |
| Degerstrøm & Østerud (128) | moderate | low | low | low | low | low | **moderate** |
| Dorneles et al. (129) | low | low | low | low | low | low | **low** |
| dos Santos et al. (130) | moderate | low | low | low | low | low | **moderate** |
| Dufaux et al. (131) | critical | low | low | critical | moderate | critical | **critical** |
| Edwards et al. (132) | moderate | low | low | low | low | low | **moderate** |
| Ely et al. (133) | moderate | low | low | low | low | low | **moderate** |
| Febbraio et al. (134) | low | low | low | low | low | low | **low** |
| Ferguson et al. (135) | low | low | low | low | low | low | **low** |
| Fonseca et al. (136) | low | low | low | low | low | low | **low** |
| Gagnon et al. (137) | moderate | low | low | low | low | low | **moderate** |
| García et al. (138) | moderate | low | low | critical | low | critical | **critical** |
| Garneau et al. (139) | moderate | low | low | low | low | low | **moderate** |
| Ghafourin et al. (140) | critical | low | low | low | low | low | **critical** |
| Gill et al. (141) | critical | low | low | low | low | low | **critical** |
| Giraldo et al. (142) | critical | low | low | low | low | low | **critical** |
| Górecka et al. (143) | critical | low | low | low | low | low | **critical** |
| Goto et al. (144) | moderate | low | moderate | moderate | low | low | **moderate** |
| Goto et al. (145) | moderate | low | low | low | low | low | **moderate** |
| Gough et al. (146) | serious | low | moderate | low | low | low | **serious** |
| Gray et al. (147) | moderate | low | low | low | low | low | **moderate** |
| Gusba et al. (148) | low | low | low | low | low | low | **low** |
| Hacker et al. (149) | moderate | low | low | low | low | low | **moderate** |
| Hackney et al. (150) | critical | low | low | low | serious | low | **critical** |
| Harris et al. (151) | critical | low | low | low | serious | low | **critical** |
| He et al. (152) | moderate | low | low | low | low | low | **moderate** |
| Hojman et al. (155) | critical | low | low | low | serious | low | **critical** |
| Islam et al. (156) | low | low | low | low | low | low | **low** |
| Islam et al. (157) | low | low | low | low | low | low | **low** |
| Jimenez et al. (158) | moderate | low | low | low | low | low | **moderate** |
| Joisten et al. (159) | low | low | low | low | low | low | **low** |
| Jürimäe et al. (160) | moderate | low | low | low | low | low | **moderate** |
| Kakanis et al. (161) | low | low | low | low | low | low | **low** |
| Kastelein et al. (162) | low | low | low | low | low | low | **low** |
| Kastelein et al. (163) | low | low | low | low | low | low | **low** |
| Kim et al. (164) | low | low | low | low | low | low | **low** |
| Kon & Tanimura (165) | low | low | low | low | low | low | **low** |
| Krzemiński et al. (166) | critical | low | low | low | low | low | **critical** |
| Kuhne et al. (167) | critical | low | low | low | low | low | **critical** |
| Landers-Ramos et al. (168) | critical | low | low | low | low | low | **critical** |
| Larsen et al. (169) | moderate | low | moderate | moderate | low | critical | **critical** |
| LaVoy et al. (170) | serious | low | low | low | low | low | **serious** |
| Leggate et al. (171) | moderate | low | low | low | low | low | **moderate** |
| Li & Gleeson (172) | moderate | low | low | low | low | low | **moderate** |
| Lira et al. (173) | serious | low | low | low | low | low | **serious** |
| Lobo et al. (174) | moderate | low | low | low | low | low | **moderate** |
| MacNeil et al. (175) | critical | moderate | low | serious | low | low | **critical** |
| Maharaj et al. (176) | low | low | low | low | low | low | **low** |
| Marcucci‑Barbosa et al. (177) | moderate | low | low | low | low | low | **moderate** |
| Marklund et al. (178) | moderate | low | low | low | low | low | **moderate** |
| Markus et al. (179) | low | low | low | low | low | serious | **serious** |
| McCormick et al. (180) | moderate | low | low | low | low | low | **moderate** |
| McKay et al. (181) | serious | low | low | low | low | low | **serious** |
| McKenzie et al. (182) | moderate | low | low | low | low | low | **moderate** |
| Mendham et al. (183) | moderate | low | low | low | low | low | **moderate** |
| Mills et al. (184) | low | low | low | low | low | low | **low** |
| Minuzzi et al. (185) | low | low | low | low | low | low | **low** |
| Moldoveanu et al. (186) | serious | low | low | low | low | low | **serious** |
| Neidhart et al. (187) | critical | low | low | low | low | low | **critical** |
| Nelson et al. (188) | moderate | low | low | low | low | low | **moderate** |
| Newlin et al. (189) | low | low | low | low | low | low | **low** |
| Nieman et al. (190) | serious | low | low | low | low | low | **serious** |
| Nieman et al. (191) | moderate | low | low | low | low | low | **moderate** |
| Nieman et al. (192) | serious | low | low | low | low | low | **serious** |
| Nieman et al. (193) | serious | low | low | low | low | low | **serious** |
| Nieman et al. (194) | critical | low | low | low | low | low | **critical** |
| Niess et al. (195) | critical | low | low | low | low | low | **critical** |
| Niess et al. (196) | critical | low | low | low | low | low | **critical** |
| Osborne et al. (197) | low | low | low | low | low | low | **low** |
| Ostrowski et al. (198) | moderate | low | low | low | low | low | **moderate** |
| Pasqua et al. (199) | critical | low | low | low | low | low | **critical** |
| Pawłowska et al. (200) | critical | low | low | low | low | low | **critical** |
| Peake et al. (201) | serious | low | low | low | low | low | **serious** |
| Peake et al. (202) | serious | low | low | low | low | low | **serious** |
| Peake et al. (203) | critical | low | low | low | low | low | **critical** |
| Peake et al. (204) | low | low | low | low | low | low | **low** |
| Peeling et al. (205) | moderate | low | low | low | low | low | **moderate** |
| Peeling et al. (206) | critical | low | low | low | low | low | **critical** |
| Peeling et al. (207) | low | low | low | low | low | low | **low** |
| Peeling et al. (208) | moderate | low | low | low | low | low | **moderate** |
| Pilat et al. (209) | serious | low | low | low | low | low | **serious** |
| Panizo González et al. (210) | critical | low | low | low | low | low | **critical** |
| Pistilli et al. (211) | serious | low | low | low | low | low | **serious** |
| Pokora et al. (212) | serious | low | low | low | low | low | **serious** |
| Pournot et al. (213) | low | low | low | critical | low | low | **critical** |
| Pozzolo et al. (214) | serious | low | low | low | low | low | **serious** |
| Proschinger et al. (215) | low | low | low | low | low | low | **low** |
| Rämson et al. (216) | serious | low | low | low | low | low | **serious** |
| Reichel et al. (217) | moderate | low | low | low | low | low | **moderate** |
| Rinnov et al. (218) | serious | low | low | low | low | low | **serious** |
| Robson-Ansley et al. (219) | critical | low | low | low | low | low | **critical** |
| Rohde et al. (220) | critical | low | low | low | low | critical | **critical** |
| Ronsen et al. (221) | low | low | low | low | low | low | **low** |
| Said et al. (222) | serious | low | low | low | low | low | **serious** |
| Sawai et al. (223) | moderate | low | low | low | low | low | **moderate** |
| Scharhag et al. (224) | Moderate | low | low | moderate | low | low | **moderate** |
| Scott et al. (225) | low | low | low | low | low | low | **low** |
| Scott et al. (226) | low | low | low | low | low | low | **low** |
| Shojaei et al. (227) | moderate | low | low | low | low | low | **moderate** |
| Shojaei et al. (228) | moderate | low | low | low | low | low | **moderate** |
| Silva Acevedo et al. (229) | moderate | low | low | low | low | low | **moderate** |
| Sim et al. (230) | moderate | low | low | low | low | low | **moderate** |
| Sim et al. (231) | moderate | low | low | low | low | low | **moderate** |
| Simpson et al. (232) | critical | low | low | low | low | low | **critical** |
| Siqueira et al. (233) | critical | low | low | low | low | low | **critical** |
| Smith et al. (234) | moderate | low | moderate | moderate | low | serious | **serious** |
| Snipe et al. (235) | serious | low | low | low | low | low | **serious** |
| Spanoudaki et al. (236) | moderate | low | low | low | low | low | **moderate** |
| Starkie et al. (237) | moderate | low | low | low | low | low | **moderate** |
| Starkie et al. (238) | critical | low | low | low | low | low | **critical** |
| Starkie et al. (239) | critical | low | low | low | low | low | **critical** |
| Starzak et al. (240) | moderate | low | low | low | low | low | **moderate** |
| Steensberg et al. (241) | critical | low | low | low | low | low | **critical** |
| Sugama et al. (242) | critical | low | low | low | low | low | **critical** |
| Sugama et al. (243) | critical | low | low | low | low | low | **critical** |
| Sumi et al. (244) | moderate | low | low | low | low | low | **moderate** |
| Sureda et al. (245) | moderate | low | low | low | low | low | **moderate** |
| Suzuki et al. (246) | critical | low | low | low | low | low | **critical** |
| Svendsen et al. (247) | low | low | low | low | low | low | **low** |
| Tartibian et al. (248) | low | low | low | low | low | low | **low** |
| Timmons et al. (249) | serious | low | low | low | low | low | **serious** |
| Timmons et al. (250) | serious | low | low | low | low | low | **serious** |
| Tsuchiya & Goto (251) | moderate | low | low | low | low | low | **moderate** |
| Ullum et al. (252) | serious | low | low | low | low | low | **Serious** |
| Vaisberg et al. (253) | critical | low | low | low | low | low | **critical** |
| van de Vyver & Myburgh (254) | critical | low | low | low | low | low | **critical** |
| van de Vyver & Myburgh (255) | critical | low | low | low | low | low | **critical** |
| van de Vyver et al. (256) | critical | low | low | low | low | low | **critical** |
| Venhorst et al. (257) | critical | low | low | low | low | low | **critical** |
| Wadley et al. (258) | low | low | low | low | low | low | **low** |
| Wadley et al. (259) | low | low | low | low | low | low | **low** |
| Wahl et al. (260) | moderate | low | low | low | low | low | **moderate** |
| Wallberg et al. (261) | critical | low | low | low | low | low | **critical** |
| Walshe et al. (262) | low | low | low | low | low | low | **low** |
| Wang et al. (263) | serious | low | low | critical | low | low | **critical** |
| Woo et al. (264) | critical | low | low | low | low | low | **critical** |
| Zaldivar et al. (265) | critical | low | low | low | low | low | **critical** |
| Zheng et al. (266) | low | low | low | low | low | low | **low** |

# Table S4 Certainty of evidence

| Myokine | Study design | Risk of bias | Inconsis-tency | Indirect-ness | Impreci-sion | Publica-tion bias | large magnitude of the effect | dose-response relation-ship | Likelihood of con-founding | overall |
| --- | --- | --- | --- | --- | --- | --- | --- | --- | --- | --- |
| IL-6 | Non-RCT  but evaluated with ROBINS-I tool | -1 | No | No | No | -1 | +1 | +1 | No | **HIGH** |
| IL-1ra | Non-RCT  but evaluated with ROBINS-I tool | -1 | No | No | No | No | No | No | No | **LOW** |
| IL-10 | Non-RCT  But evaluated with ROBINS-I tool | -1 | No | No | No | No | No | +1 | No | **HIGH** |
| IL-8 | Non-RCT  but evaluated with ROBINS-I tool | -1 | No | No | No | No | +1 | +1 | No | **HIGH** |
| IL-15 | Non-RCT  but evaluated with ROBINS-I tool | No | No | No | No | -1 | No | No | No | **LOW** |
| TNF-α | Non-RCT  but evaluated with ROBINS-I tool | -1 | No | No | No | -1 | No | No | No | **VERY LOW** |

# Table S5 Main results meta-analyses

| **Main Analysis Myokines** | | | | | | | | **Egger's test** | | | **Trim and Fill** | | | | | | |
| --- | --- | --- | --- | --- | --- | --- | --- | --- | --- | --- | --- | --- | --- | --- | --- | --- | --- |
| **Para-meter** | **effect size after sensitivity analysis + CI** | **prediction interval** | **I² [%]** | **tau^2^** | **Outliers/ Influential**  **cases** | **original k** | **%** | **t** | **df** | **P value** |  |  |  |  |  |  |  |
| IL-6 | 1.20  [1.02;1.39] | [−2.55; 4.96] | 88% | 0.116 | / | 206 | / | 8.23 | 204 | <0.001 | missing studies | estimate | SE | Z value | P value | ci.lb | ci.ub |
|  |  |  |  |  |  |  |  |  |  |  | 0 (SE = 2.1434) | n.a. | n.a. | n.a. | n.a. | n.a. | n.a. |
| IL-1ra | 0.40  [0.26; 0.53] | [−0.40; 1.19] | 64% | 0.145 | / | 31 | / | 0.84 | 29 | 0.408 | missing studies | estimate | SE | Z value | P value | ci.lb | ci.ub |
|  |  |  |  |  |  |  |  |  |  |  | n.a. | n.a. | n.a. | n.a. | n.a. | n.a. | n.a. |
| IL-10 | 0.45  [0.31; 0.59] | [−0.75; 1.65] | 88% | 0.356 | / | 67 | / | -0.99 | 65 | 0.339 | missing studies | estimate | SE | Z value | P value | ci.lb | ci.ub |
|  |  |  |  |  |  |  |  |  |  |  | n.a. | n.a. | n.a. | n.a. | n.a. | n.a. | n.a. |
| IL-8 | 0.71  [-1.00; 0.95] | [−1.00; 2.42] | 89% | 0.709 | / | 49 | / | 1.64 | 47 | 0.107 | missing studies | estimate | SE | Z value | P value | ci.lb | ci.ub |
|  |  |  |  |  |  |  |  |  |  |  | n.a. | n.a. | n.a. | n.a. | n.a. | n.a. | n.a. |
| IL-15 | 0.37  [0.13; 0.61] | [−0.42; 1.15] | 80% | 0.116 | / | 12 | / | -2.24 | 10 | 0.049 | missing studies | estimate | SE | Z value | P value | ci.lb | ci.ub |
|  |  |  |  |  |  |  |  |  |  |  | 0 (SE = 2.1434) | n.a. | n.a. | n.a. | n.a. | n.a. | n.a. |
| TNF-α | 0.47  [0.32; 0.61] | [−0.93; 1.87] | 85% | 0.492 | / | 87 | / | 5.61 | 84 | <0.001 | missing studies | estimate | SE | Z value | P value | ci.lb | ci.ub |
|  |  |  |  |  |  |  |  |  |  |  | 0 (SE = 4.8066) | n.a. | n.a. | n.a. | n.a. | n.a. | n.a. |

#
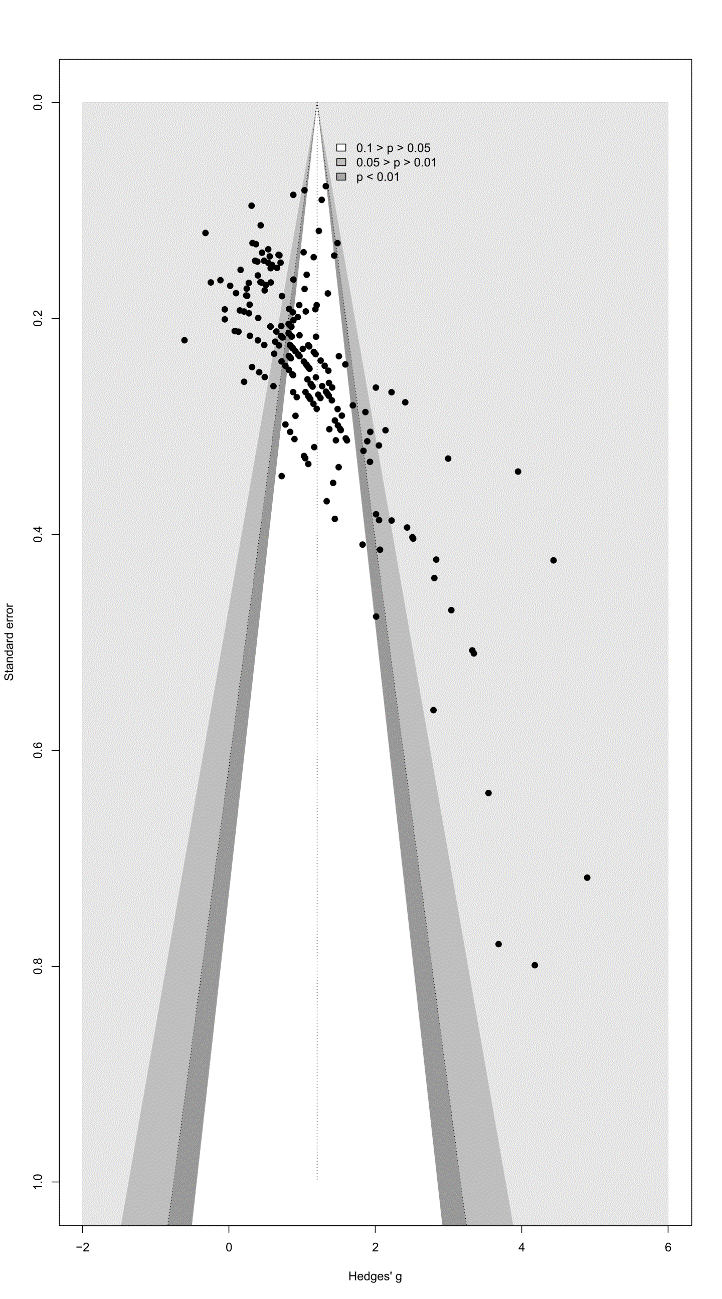

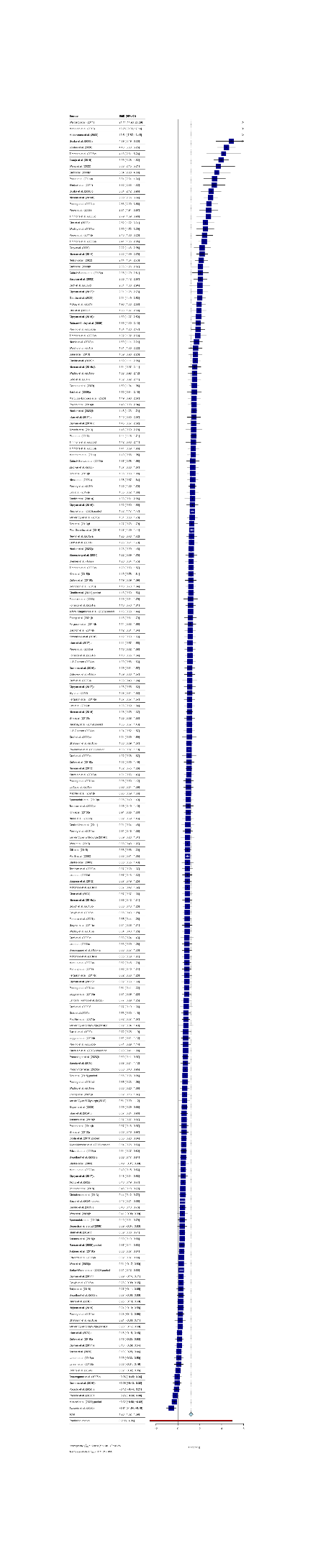
Plots IL-6

**Fig. S1** Funnel Plot (A) and Forrest plot (B) for IL-6

**B**

**A**

#
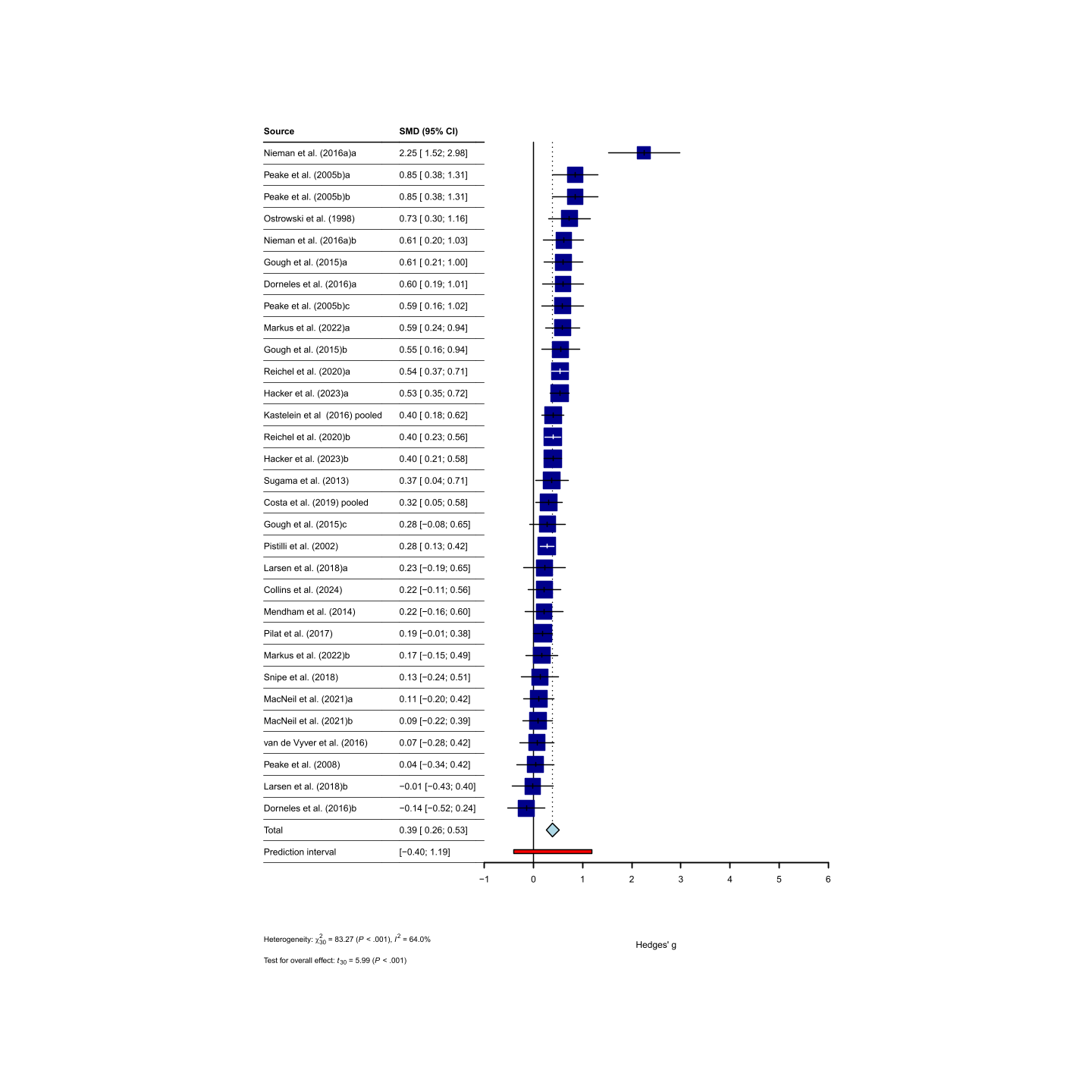

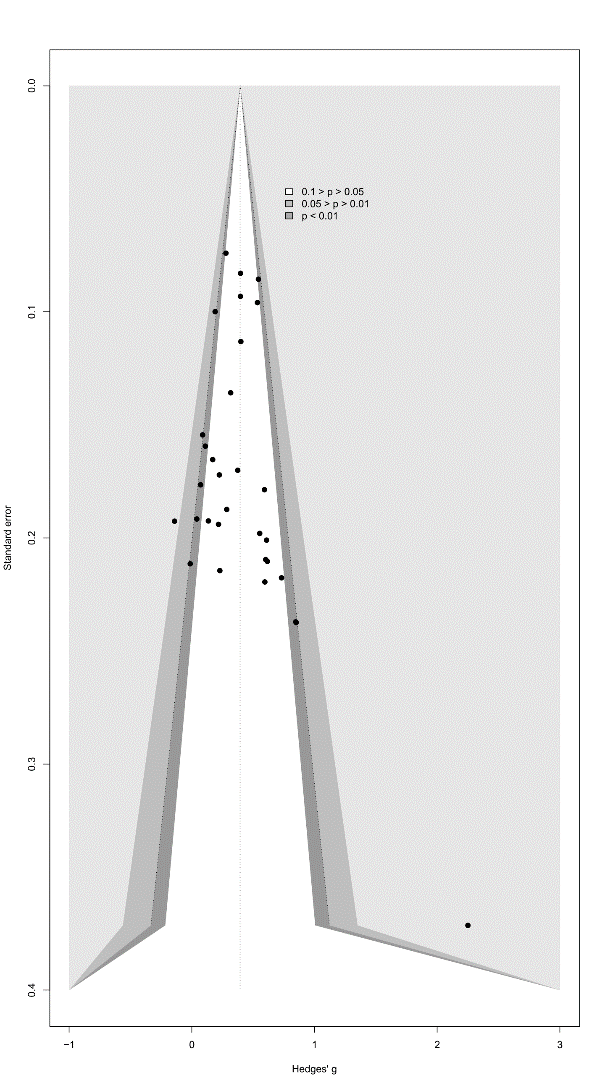
Plots IL-1ra

**B**

**A**

**Fig. S2** Funnel Plot (A) and Forrest plot (B) for IL-1ra

#
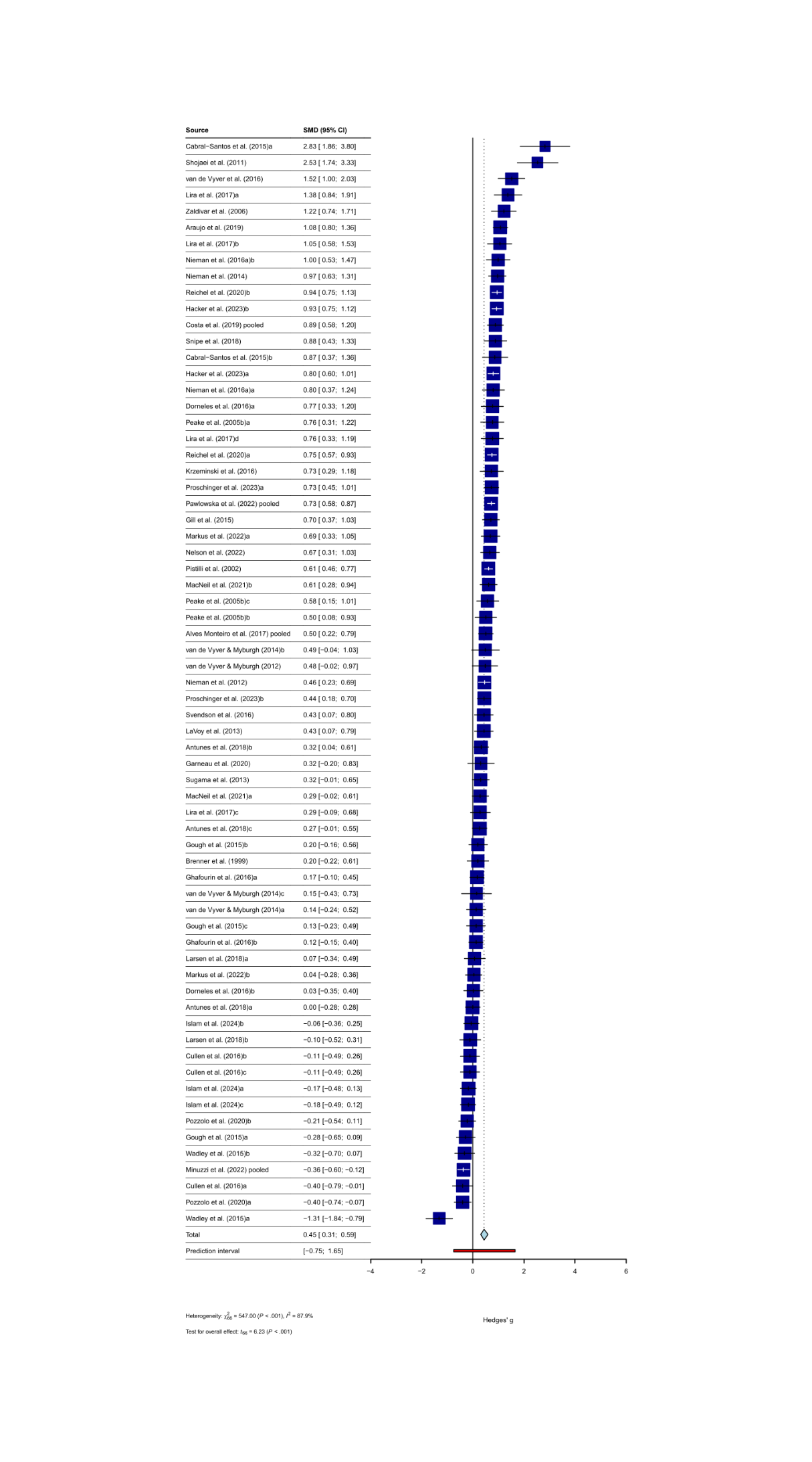

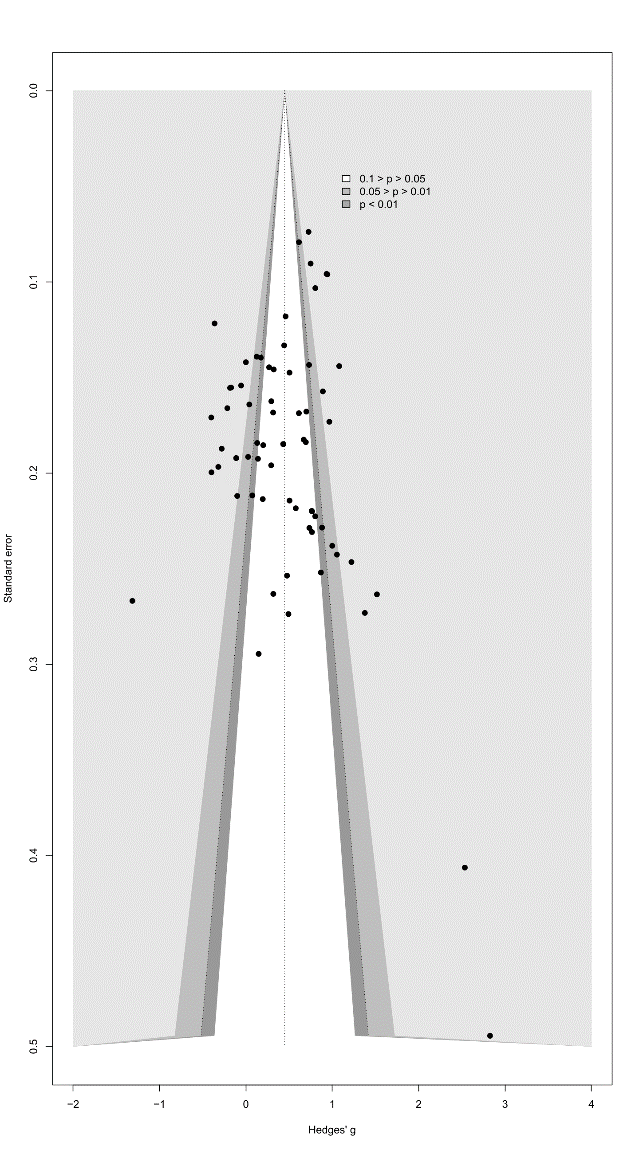
Plots IL-10

**Fig. S3** Funnel Plot (A) and Forrest plot (B) for IL-10

**B**

**A**

#
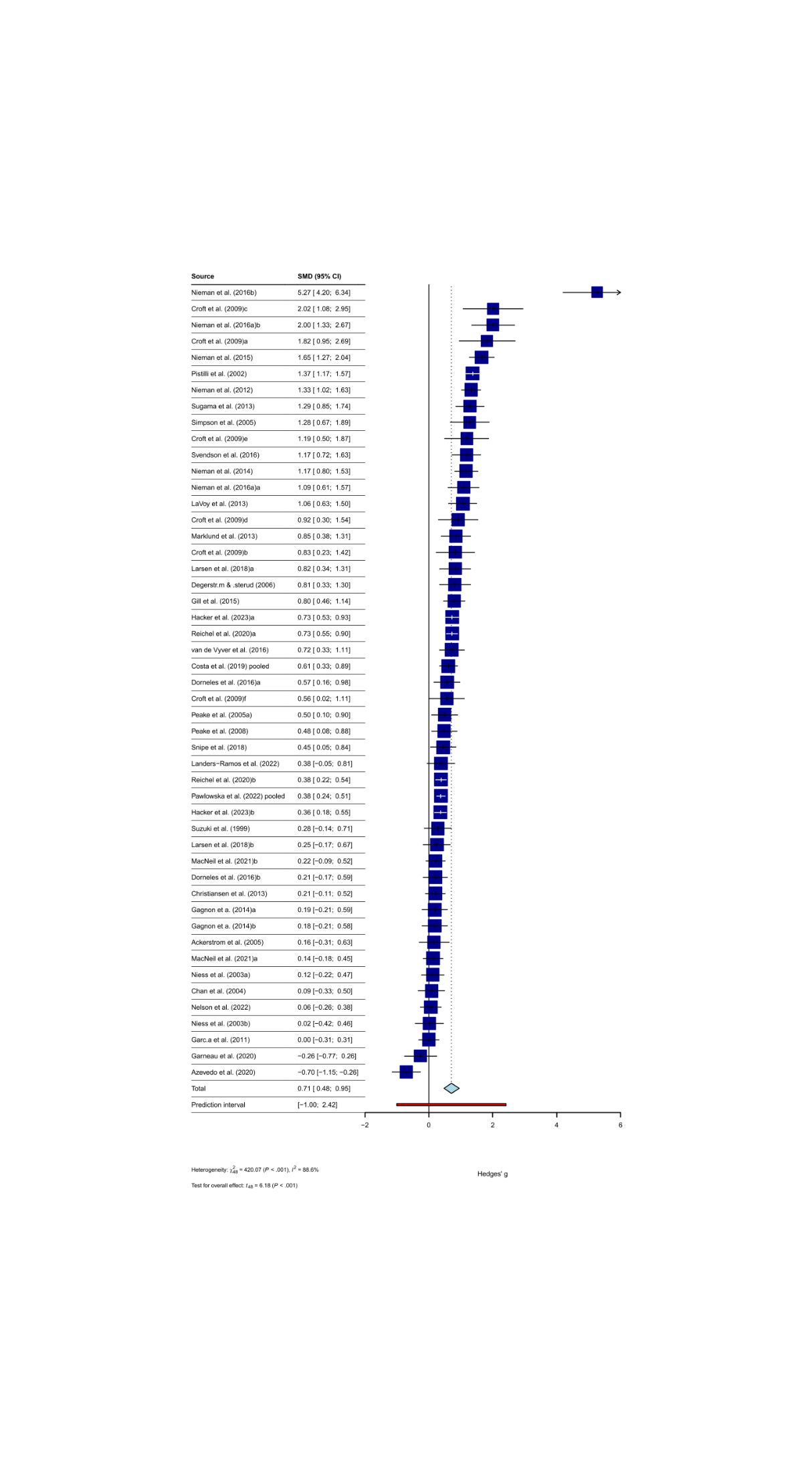

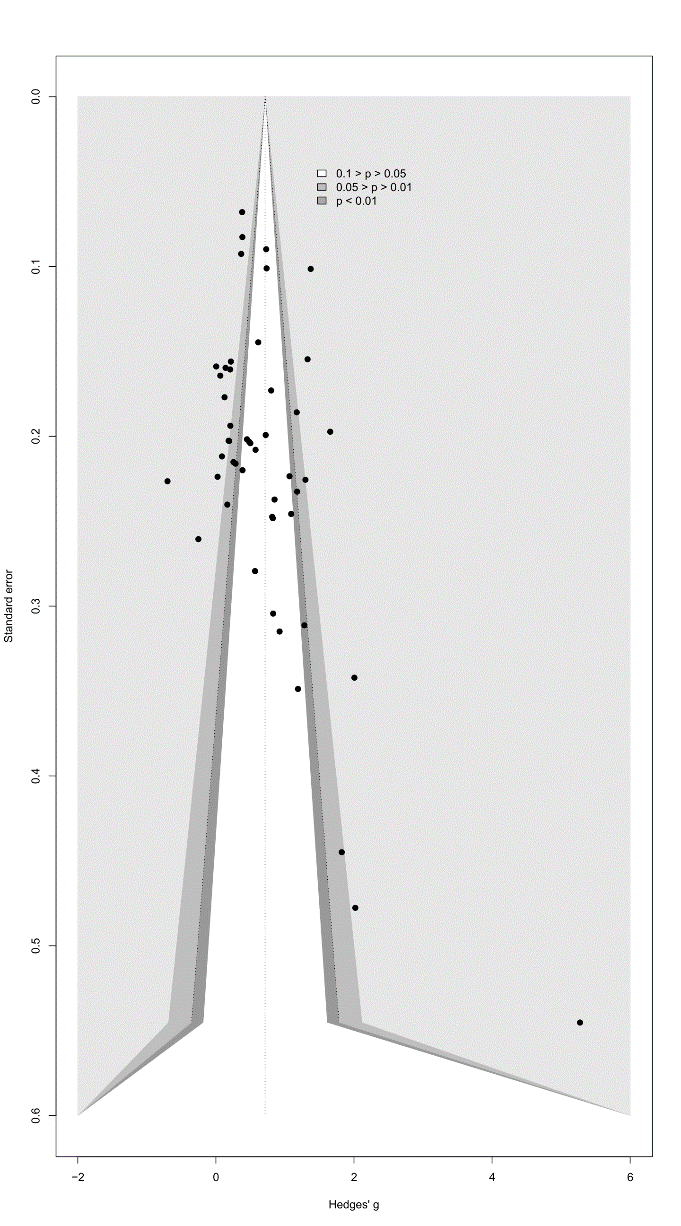
Plots IL-8

**Fig. S4** Funnel Plot (A) and Forrest plot (B) for IL-8

**A**

**B**

#
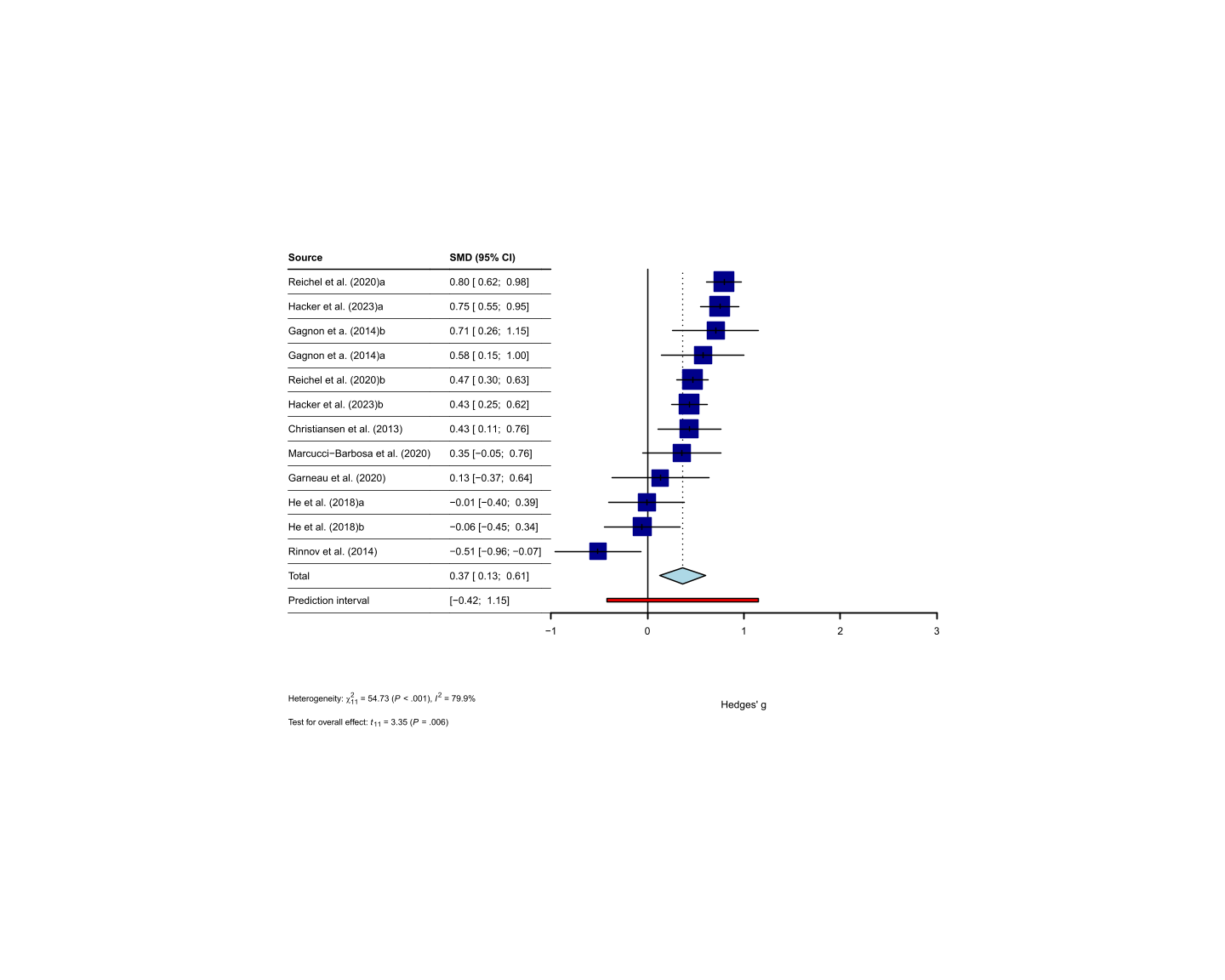

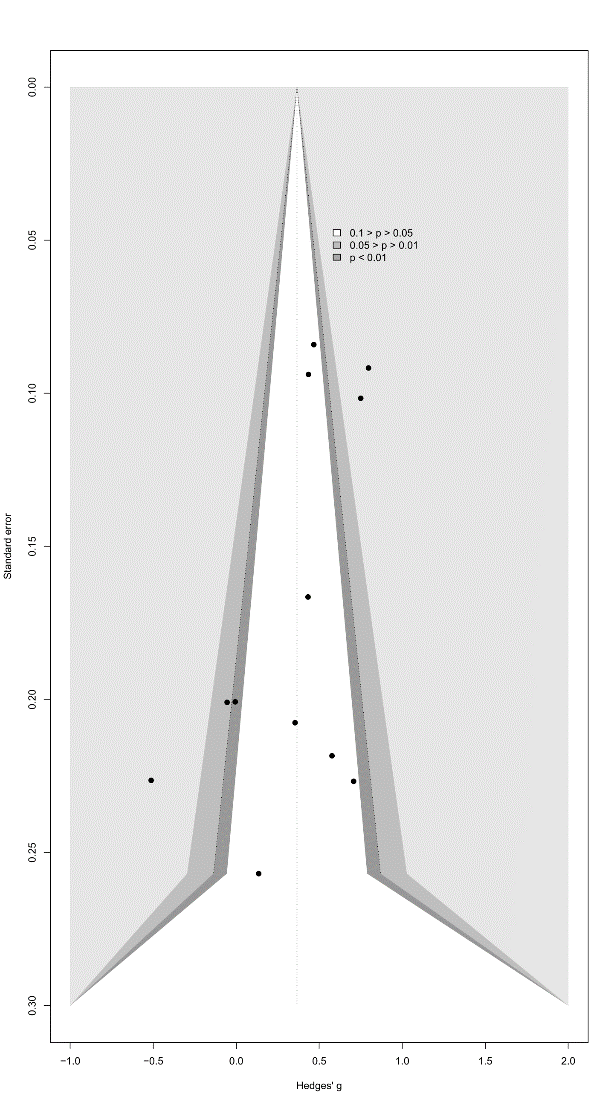
Plots IL-15

**Fig. S5** Funnel Plot (A) and Forrest plot (B) for IL-15

**B**

**A**

#
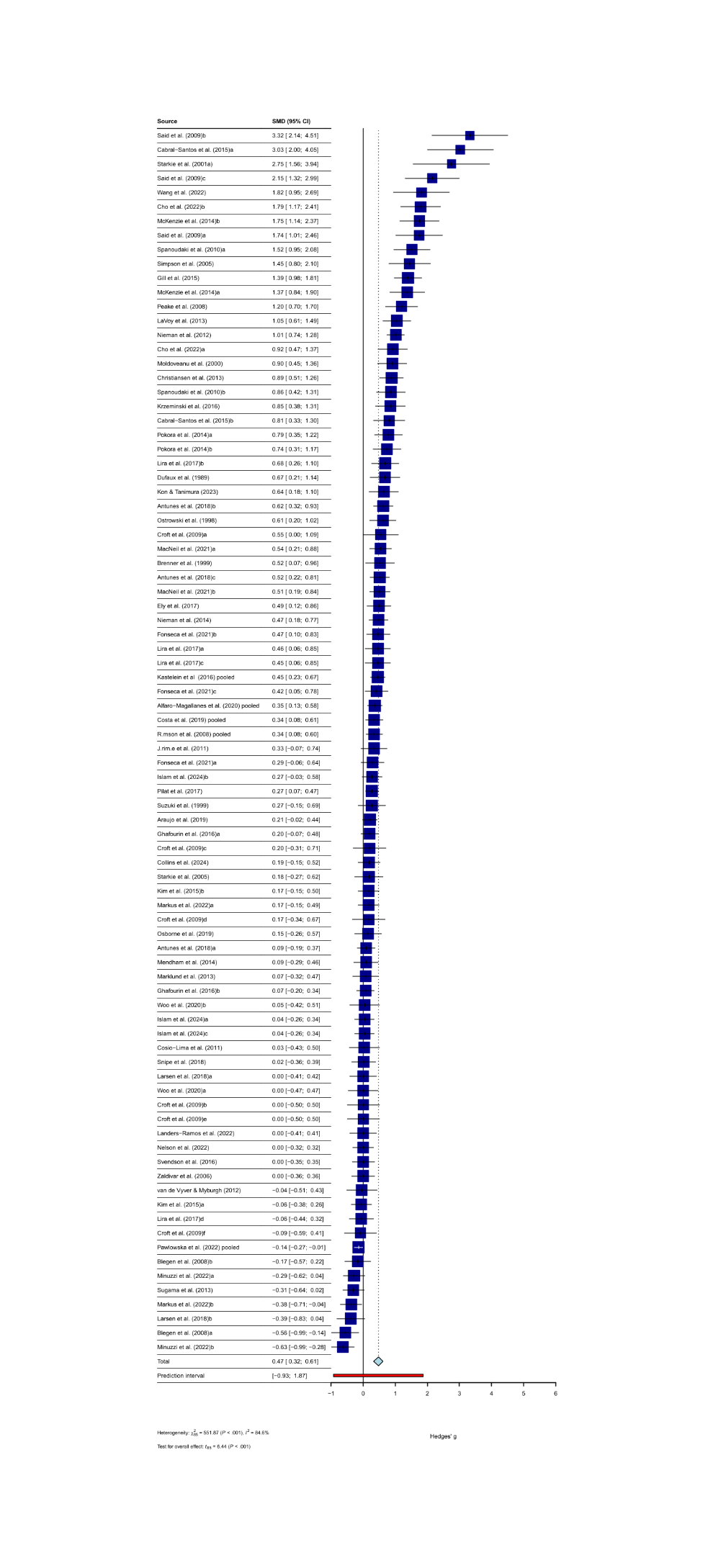

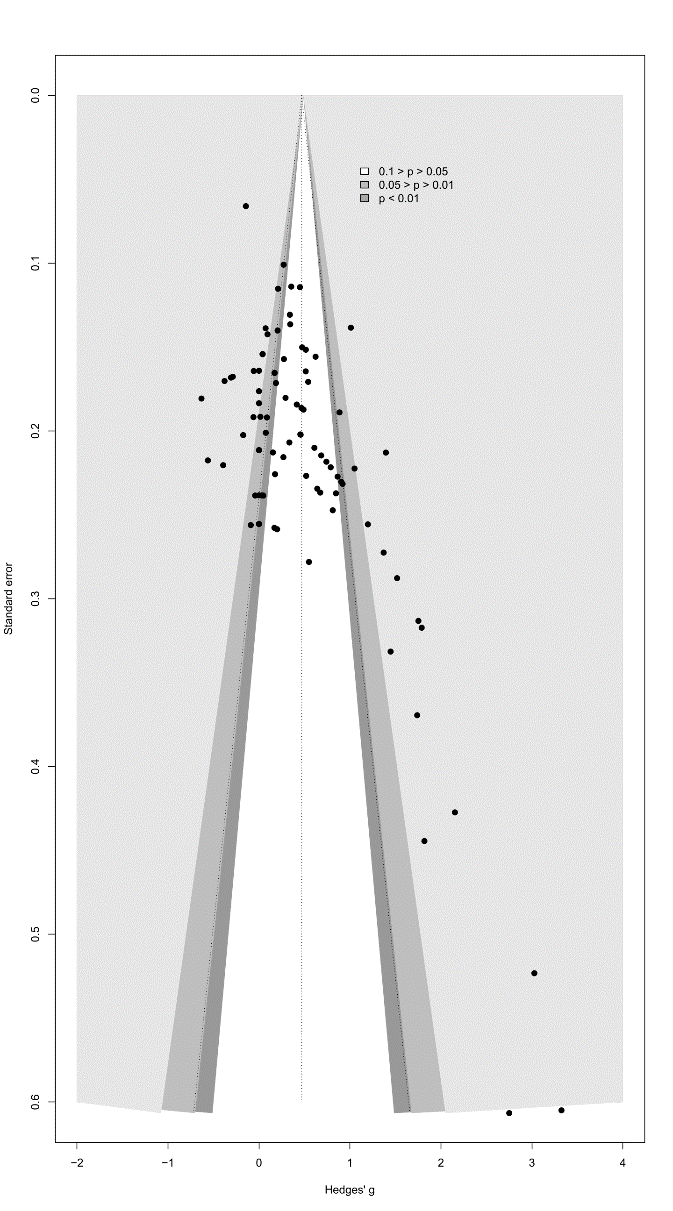
Plots TNF-α

**Fig. S6** Funnel Plot (A) and Forrest plot (B) for IL-1ra

**B**

**A**

# Results moderator analysis

## Risk of bias

| **RISK OF BIAS** | | | | | | | | | | |
| --- | --- | --- | --- | --- | --- | --- | --- | --- | --- | --- |
| **Parameter** |  | **Total** | **Post hoc** | **I²** | **k** | **tau** | **tau²** | **Q** | **df** | **P value** |
| **IL-6** | **low** | 1.31 [098; 1.64] |  | 99.5% | 40 | 1.056 | 1.115 | 5.06 | 3 | 0.168 |
|  | **moderate** | 1.01 [0.83; 1.18] |  | 99.6% | 81 | 1.436 | 2.061 |  |  |  |
|  | **serious** | 1.40 [0.99; 1.81] |  | 99.5% | 41 | 1.691 | 2.858 |  |  |  |
|  | **critical** | 1.05 [0.70; 1.39] |  | 99.5% | 44 | 2.744 | 7.527 |  |  |  |
|  |  |  |  |  |  |  |  |  |  |  |
| **Parameter** |  | **Total** | **Post hoc** | **I²** | **k** | **tau** | **tau²** | **Q** | **df** | **P value** |
| **IL-1ra** | **low** | 0.29 [-0.68; 1.24] | Moderate > critical***  Serious > critical** | 99.1% | 3 | 0.383 | 0.146 | 23.57 | 3 | <0.001 |
|  | **moderate** | 0.45 [0.29; 0.60] |  | 98.7% | 7 | 0.168 | 0.028 |  |  |  |
|  | **serious** | 0.61 [0.29; 0.93] |  | 98.5% | 13 | 0.541 | 0.292 |  |  |  |
|  | **critical** | 0.13 [0.03; 0.23] |  | 92.9% | 8 | 0.116 | 0.014 |  |  |  |
|  |  |  |  |  |  |  |  |  |  |  |
| **Parameter** |  | **Total** | **Post hoc** | **I²** | **k** | **tau** | **tau²** | **Q** | **df** | **P value** |
| **IL-10** | **low** | 0.00 [-0.40;0.40] | Moderate > low**  Serious > low*  Critical > low* | 99.7% | 11 | 0.593 | 0.352 | 8.44 | 3 | 0.038 |
|  | **moderate** | 0.71 [0.30; 1.11] |  | 99.7% | 18 | 0.825 | 0.681 |  |  |  |
|  | **serious** | 0.48 [0.26; 0.69] |  | 99.5% | 21 | 0.470 | 0.221 |  |  |  |
|  | **critical** | 0.50 [0.28; 0.72] |  | 99.4% | 17 | 0.429 | 0.184 |  |  |  |
|  |  |  |  |  |  |  |  |  |  |  |
| **Parameter** |  | **Total** | **Post hoc** | **I²** | **k** | **tau** | **tau²** | **Q** | **df** | **P value** |
| **IL-8** | **low** | 0.65 [-0.56; 1.86] |  | 99.1% | 3 | 0.487 | 0.237 | 2.55 | 3 | 0.466 |
|  | **moderate** | 0.59 [0.31; 0.88] |  | 99.5% | 21 | 0.626 | 0.391 |  |  |  |
|  | **serious** | 1.01 [0.49; 1.53] |  | 99.6% | 9 | 0.681 | 0.464 |  |  |  |
|  | **critical** | 0.79 [0.14; 1.44] |  | 99.0% | 16 | 1.249 | 1.559 |  |  |  |
|  |  |  |  |  |  |  |  |  |  |  |
| **Parameter** |  | **Total** | **Post hoc** | **I²** | **k** | **tau** | **tau²** | **Q** | **df** | **P value** |
| **IL-15** | **moderate** | 0.42 [0.22; 0.61] | Moderate > serious*** | 99.5% | 11 | 0.290 | 0.084 | 83.75 | 1 | <0.001 |
|  | **serious** | -0.51 [-0.61; -0.41] |  | - | 1 | - | - |  |  |  |
|  |  |  |  |  |  |  |  |  |  |  |
| **Parameter** |  | **Total** | **Post hoc** | **I²** | **k** | **tau** | **tau²** | **Q** | **df** | **P value** |
| **TNF-α** | **low** | 0.15 [-0.03;0.33] | Low < Moderate**  Low < Serious** | 99.2% | 15 | 0.323 | 0.104 | 12.51 | 3 | 0.006 |
|  | **moderate** | 0.58 [0.29; 0.86] |  | 99.7% | 25 | 0.704 | 0.496 |  |  |  |
|  | **serious** | 0.70 [0.33; 1.07] |  | 99.4% | 23 | 0.872 | 0.755 |  |  |  |
|  | **critical** | 0.46 [0.14; 0.78] |  | 99.4% | 23 | 0.767 | 0.588 |  |  |  |

## Sex

| **SEX** | | | | | | | | | | |
| --- | --- | --- | --- | --- | --- | --- | --- | --- | --- | --- |
| **Parameter** |  | **Total** | **Post hoc** | **I²** | **k** | **tau** | **tau²** | **Q** | **df** | **P value** |
| **IL-6** | **m** | 1.27 [1.07; 1.46] | m > mf** | 99.1% | 140 | 2.072 | 4.292 | 7.76 | 2 | 0.021 |
|  | **mf** | 0.83 [0.57; 1.08] |  | 99.8% | 28 | 0.661 | 0.437 |  |  |  |
|  | **f** | 1.05 [0.74; 1.36] |  | 99.6% | 30 | 0.900 | 0.900 |  |  |  |
|  |  |  |  |  |  |  |  |  |  |  |
| **Parameter** |  | **Total** | **Post hoc** | **I²** | **k** | **tau** | **tau²** | **Q** | **df** | **P value** |
| **IL-1ra** | **m** | 0.47 [0.22; 0.73] |  | 98.5% | 18 | 0.521 | 0.272 | 1.21 | 2 | 0.546 |
|  | **mf** | 0.32 [0.13; 0.50] |  | 99.3% | 7 | 0.200 | 0.040 |  |  |  |
|  | **f** | 0.38 [0.11; 0.65] |  | 97.1% | 6 | 0.251 | 0.063 |  |  |  |
|  |  |  |  |  |  |  |  |  |  |  |
| **Parameter** |  | **Total** | **Post hoc** | **I²** | **k** | **tau** | **tau²** | **Q** | **df** | **P value** |
| **IL-10** | **m** | 0.60 [0.39; 0.82] | m > f** | 99.4% | 40 | 0.684 | 0.468 | 6.99 | 2 | 0.030 |
|  | **mf** | 0.40 [0.16; 0.64] |  | 99.8% | 17 | 0.465 | 0.216 |  |  |  |
|  | **f** | 0.12 [-0.23; 0.47] |  | 99.2% | 8 | 0.422 | 0.179 |  |  |  |
|  |  |  |  |  |  |  |  |  |  |  |
| **Parameter** |  | **Total** | **Post hoc** | **I²** | **k** | **tau** | **tau²** | **Q** | **df** | **P value** |
| **IL-8** | **m** | 0.92 [0.58; 1.32] | m > mf* | 99.8% | 29 | 0.983 | 0.967 | 7.34 | 2 | 0.026 |
|  | **mf** | 0.37 [ 0.11; 0.63] |  | 99.6% | 14 | 0.453 | 0.205 |  |  |  |
|  | **f** | 0.56 [-0.55; 1.67] |  | 99.1% | 5 | 0.895 | 0.801 |  |  |  |
|  |  |  |  |  |  |  |  |  |  |  |
| **Parameter** |  | **Total** | **Post hoc** | **I²** | **k** | **tau** | **tau²** | **Q** | **df** | **P value** |
| **IL-15** | **m** | 0.18 [-0.30; 0.65] | mf > m* | 98.8% | 6 | 0.453 | 0.205 | 3.89 | 1 | 0.049 |
|  | **mf** | 0.58 [0.35; 0.80] |  | 99.7% | 5 | 0.181 | 0.033 |  |  |  |
|  |  |  |  |  |  |  |  |  |  |  |
| **Parameter** |  | **Total** | **Post hoc** | **I²** | **k** | **tau** | **tau²** | **Q** | **df** | **P value** |
| **TNF-α** | **m** | 0.53 [0.35; 0.71] |  | 99.5% | 67 | 0.755 | 0.570 | 0.46 | 2 | 0.794 |
|  | **mf** | 0.47 [0.14; 0.80] |  | 99.3% | 12 | 0.524 | 0.275 |  |  |  |
|  | **f** | 0.31 [-0.54; 1.15] |  | 99.7% | 7 | 0.915 | 0.837 |  |  |  |

## Experience

| **Experience** | | | | | | | | | | |
| --- | --- | --- | --- | --- | --- | --- | --- | --- | --- | --- |
| **Parameter** |  | **Total** | **Post hoc** | **I²** | **k** | **tau** | **tau²** | **Q** | **df** | **P value** |
| **IL-6** | **trained** | 1.09 [0.91; 1.27] |  | 99.5% | 91 | 1.424 | 2.029 | 3.66 | 2 | 0.160 |
|  | **active** | 0.96 [0.74; 1.19] |  | 98.7% | 47 | 0.818 | 0.699 |  |  |  |
|  | **untrained** | 1.52 [0.96; 2.08] |  | 99.2% | 40 | 3.121 | 9.738 |  |  |  |
|  |  |  |  |  |  |  |  |  |  |  |
| **Parameter** |  | **Total** | **Post hoc** | **I²** | **k** | **tau** | **tau²** | **Q** | **df** | **P value** |
| **IL-1ra** | **trained** | 0.57 [0.30; 0.84] | Trained > untrained*  Trained > active*** | 98.2% | 16 | 0.508 | 0.258 | 13.29 | 2 | 0.001 |
|  | **active** | 0.09 [-0.06; 0.25] |  | 79.6% | 4 | 0.093 | 0.009 |  |  |  |
|  | **untrained** | 0.23 [0.02; 0.44] |  | 98.4% | 7 | 0.229 | 0.053 |  |  |  |
|  |  |  |  |  |  |  |  |  |  |  |
| **Parameter** |  | **Total** | **Post hoc** | **I²** | **k** | **tau** | **tau²** | **Q** | **df** | **P value** |
| **IL-10** | **trained** | 0.47 [0.31; 0.64] |  | 99.5% | 26 | 0.404 | 0.163 | 0.22 | 2 | 0.895 |
|  | **active** | 0.56 [0.19; 0.93] |  | 99.3% | 18 | 0.755 | 0.570 |  |  |  |
|  | **untrained** | 0.47 [-0.16; 1.09] |  | 99.7% | 11 | 0.935 | 0.875 |  |  |  |
|  |  |  |  |  |  |  |  |  |  |  |
| **Parameter** |  | **Total** | **Post hoc** | **I²** | **k** | **tau** | **tau²** | **Q** | **df** | **P value** |
| **IL-8** | **trained** | 0.98 [0.53; 1.43] |  | 99.7% | 24 | 1.086 | 1.180 | 5.75 | 2 | 0.057 |
|  | **active** | 0.65 [0.20; 1.09] |  | 99.5% | 11 | 0.670 | 0.449 |  |  |  |
|  | **untrained** | 0.35 [-0.01; 0.71] |  | 99.1% | 8 | 0.432 | 0.186 |  |  |  |
|  |  |  |  |  |  |  |  |  |  |  |
| **Parameter** |  | **Total** | **Post hoc** | **I²** | **k** | **tau** | **tau²** | **Q** | **df** | **P value** |
| **IL-15** | **active** | 0.31 [-0.28; 0.91] |  | 98.9% | 5 | 0.478 | 0.229 | 1.80 | 1 | 0.180 |
|  | **untrained** | 0.02 [-0.22; 0.25] |  | 66.9% | 3 | 0.085 | 0.007 |  |  |  |
|  |  |  |  |  |  |  |  |  |  |  |
| **Parameter** |  | **Total** | **Post hoc** | **I²** | **k** | **tau** | **tau²** | **Q** | **df** | **P value** |
| **TNF-α** | **trained** | 0.58 [0.28; 0.88] |  | 99.2% | 33 | 0.873 | 0.762 | 1.49 | 2 | 0.474 |
|  | **active** | 0.45 [0.17; 0.73] |  | 99.0% | 25 | 0.703 | 0.495 |  |  |  |
|  | **untrained** | 0.69 [0.39; 1.00] |  | 99.2% | 17 | 0.591 | 0.349 |  |  |  |

## Intensity

| **Intensity** | | | | | | | | | | |
| --- | --- | --- | --- | --- | --- | --- | --- | --- | --- | --- |
| **Parameter** |  | **Total** | **Post hoc** | **I²** | **k** | **tau** | **tau²** | **Q** | **df** | **P value** |
| **IL-6** | **moderate** | 1.22 [0.71; 1.72] |  | 99.5% | 56 | 3.123 | 9.750 | 0.18 | 2 | 0.916 |
|  | **vigorous** | 1.17 [1.02; 1.32] |  | 99.6% | 105 | 0.817 | 0.668 |  |  |  |
|  | **maximal** | 1.11 [0.82; 1.41] |  | 99.7% | 28 | 0.779 | 0.606 |  |  |  |
|  |  |  |  |  |  |  |  |  |  |  |
| **Parameter** |  | **Total** | **Post hoc** | **I²** | **k** | **tau** | **tau²** | **Q** | **df** | **P value** |
| **IL-1ra** | **moderate** | 0.33 [0.15; 0.50] |  | 98.2% | 12 | 0.270 | 0.073 | 2.50 | 2 | 0.286 |
|  | **vigorous** | 0.56 [0.22; 0.90] |  | 99.1% | 13 | 0.566 | 0.321 |  |  |  |
|  | **maximal** | 0.28 [0.02; 0.54] |  | 99.0% | 5 | 0.206 | 0.043 |  |  |  |
|  |  |  |  |  |  |  |  |  |  |  |
| **Parameter** |  | **Total** | **Post hoc** | **I²** | **k** | **tau** | **tau²** | **Q** | **df** | **P value** |
| **IL-10** | **moderate** | 0.41 [0.08; 0.75] |  | 99.7% | 22 | 0.764 | 0.584 | 0.79 | 2 | 0.680 |
|  | **vigorous** | 0.39 [0.21; 0.57] |  | 99.7% | 26 | 0.444 | 0.197 |  |  |  |
|  | **maximal** | 0.64 [0.04; 1.23] |  | 99.8% | 10 | 0.846 | 0.716 |  |  |  |
|  |  |  |  |  |  |  |  |  |  |  |
| **Parameter** |  | **Total** | **Post hoc** | **I²** | **k** | **tau** | **tau²** | **Q** | **df** | **P value** |
| **IL-8** | **moderate** | 0.44 [0.19; 0.69] | Maximal > moderate**  Vigorous > moderate* | 99.8% | 16 | 0.473 | 0.223 | 7.35 | 2 | 0.025 |
|  | **vigorous** | 0.85 [0.30; 1.40] |  | 99.6% | 20 | 1.200 | 1.439 |  |  |  |
|  | **maximal** | 0.96 [0.59; 1.32] |  | 99.2% | 11 | 0.544 | 0.296 |  |  |  |
|  |  |  |  |  |  |  |  |  |  |  |
| **Parameter** |  | **Total** | **Post hoc** | **I²** | **k** | **tau** | **tau²** | **Q** | **df** | **P value** |
| **IL-15** | **moderate** | 0.09 [-0.45; 0.62] |  | 99.0% | 5 | 0.430 | 0.185 | 4.92 | 2 | 0.085 |
|  | **vigorous** | 0.51 [0.06; 0.96] |  | 99.5% | 4 | 0.281 | 0.079 |  |  |  |
|  | **maximal** | 0.63 [-1.46; 2.72] |  | 99.9% | 2 | 0.233 | 0.054 |  |  |  |
|  |  |  |  |  |  |  |  |  |  |  |
| **Parameter** |  | **Total** | **Post hoc** | **I²** | **k** | **tau** | **tau²** | **Q** | **df** | **P value** |
| **TNF-α** | **moderate** | 0.54 [0.30; 0.78] |  | 99.6% | 28 | 0.648 | 0.420 | 2.3 | 2 | 0.316 |
|  | **vigorous** | 0.39 [0.19; 0.58] |  | 99.3% | 31 | 0.533 | 0.284 |  |  |  |
|  | **maximal** | 0.77 [0.20; 1.34] |  | 98.7% | 17 | 1.121 | 1.258 |  |  |  |

## Exercise type

| **Exercise type** | | | | | | | | | | |
| --- | --- | --- | --- | --- | --- | --- | --- | --- | --- | --- |
| **Parameter** |  | **Total** | **Post hoc** | **I²** | **k** | **tau** | **tau²** | **Q** | **df** | **P value** |
| **IL-6** | **Cycling** | 1.28 [1.00; 1.57] |  | 99.5% | 84 | 1.866 | 3.481 | 1.59 | 1 | 0.207 |
|  | **Running** | 1.08 [0.94; 1.22] |  | 99.6% | 117 | 1.755 | 3.80 |  |  |  |
|  |  |  |  |  |  |  |  |  |  |  |
| **Parameter** |  | **Total** | **Post hoc** | **I²** | **k** | **tau** | **tau²** | **Q** | **df** | **P value** |
| **IL-1ra** | **Cycling** | 0.21 [0.08; 0.34] | Running > Cycling** | 97.6% | 10 | 0.181 | 0.033 | 6.89 | 1 | 0.009 |
|  | **Running** | 0.52 [0.30; 0.74] |  | 99.1% | 20 | 0.478 | 0.228 |  |  |  |
|  |  |  |  |  |  |  |  |  |  |  |
| **Parameter** |  | **Total** | **Post hoc** | **I²** | **k** | **tau** | **tau²** | **Q** | **df** | **P value** |
| **IL-10** | **Cycling** | 0.24 [-0.04; 0.51] | Running > Cycling** | 99.7% | 27 | 0.691 | 0.478 | 6.22 | 1 | 0.013 |
|  | **Running** | 0.63 [0.46; 0.79] |  | 99.7% | 39 | 0.540 | 0.292 |  |  |  |
|  |  |  |  |  |  |  |  |  |  |  |
| **Parameter** |  | **Total** | **Post hoc** | **I²** | **k** | **tau** | **tau²** | **Q** | **df** | **P value** |
| **IL-8** | **Cycling** | 0.75 [0.23; 1.27] |  | 99.5% | 21 | 1.169 | 1.366 | 0.02 | 1 | 0.889 |
|  | **Running** | 0.71 [0.48; 0.94] |  | 99.7% | 27 | 0.584 | 0.341 |  |  |  |
|  |  |  |  |  |  |  |  |  |  |  |
| **Parameter** |  | **Total** | **Post hoc** | **I²** | **k** | **tau** | **tau²** | **Q** | **df** | **P value** |
| **IL-15** | **Cycling** | 0.27 [-0.33; 0.87] |  | 98.9% | 5 | 0.483 | 0.233 | 0.25 | 1 | 0.617 |
|  | **Running** | 0.39 [0.09; 0.70] |  | 99.7% | 7 | 0.330 | 0.109 |  |  |  |
|  |  |  |  |  |  |  |  |  |  |  |
| **Parameter** |  | **Total** | **Post hoc** | **I²** | **k** | **tau** | **tau²** | **Q** | **df** | **P value** |
| **TNF-α** | **Cycling** | 0.36 [0.21; 0.51] | Running > Cycling** | 99.7% | 37 | 0.442 | 0.195 | 3.88 | 1 | 0.049 |
|  | **Running** | 0.65 [0.39; 0.91] |  | 99.1% | 46 | 0.898 | 0.806 |  |  |  |

## Load

| **Load** | | | | | | | | | | |
| --- | --- | --- | --- | --- | --- | --- | --- | --- | --- | --- |
| **Parameter** |  | **Total** | **Post hoc** | **I²** | **k** | **tau** | **tau²** | **Q** | **df** | **P value** |
| **IL-6** | **Continuous** | 1.19 [1.03; 1.35] |  | 99.6% | 155 | 1.878 | 3.528 | 0.72 | 1 | 0.396 |
|  | **Intermittent** | 1.05 [0.77; 1.33] |  | 99.4% | 51 | 1.453 | 2.111 |  |  |  |
|  |  |  |  |  |  |  |  |  |  |  |
| **Parameter** |  | **Total** | **Post hoc** | **I²** | **k** | **tau** | **tau²** | **Q** | **df** | **P value** |
| **IL-1ra** | **Continuous** | 0.45 [0.28; 0.62] |  | 99.0% | 27 | 0.429 | 0.184 | 2.22 | 1 | 0.137 |
|  | **Intermittent** | 0.19 [-0.31; 0.69] |  | 98.3% | 4 | 0.311 | 0.097 |  |  |  |
|  |  |  |  |  |  |  |  |  |  |  |
| **Parameter** |  | **Total** | **Post hoc** | **I²** | **k** | **tau** | **tau²** | **Q** | **df** | **P value** |
| **IL-10** | **Continuous** | 0.46 [0.29; 0.62] |  | 99.7% | 50 | 0.574 | 0.329 | 0.04 | 1 | 0.842 |
|  | **Intermittent** | 0.50 [0.10; 0.89] |  | 99.6% | 17 | 0.785 | 0.617 |  |  |  |
|  |  |  |  |  |  |  |  |  |  |  |
| **Parameter** |  | **Total** | **Post hoc** | **I²** | **k** | **tau** | **tau²** | **Q** | **df** | **P value** |
| **IL-8** | **Continuous** | 0.74 [0.45; 1.03] |  | 99.8% | 38 | 0.912 | 0.832 | 0.00 | 1 | 0.986 |
|  | **Intermittent** | 0.73 [0.23; 1.24] |  | 98.8% | 11 | 0.756 | 0.572 |  |  |  |
|  |  |  |  |  |  |  |  |  |  |  |
| **Parameter** |  | **Total** | **Post hoc** | **I²** | **k** | **tau** | **tau²** | **Q** | **df** | **P value** |
| **IL-15** | **Continuous** | 0.42 [0.14; 0.69] | Con > Int*** | 99.6% | 10 | 0.380 | 0.145 | 13.16 | 1 | 0.0003 |
|  | **Intermittent** | -0.03 [-0.34; 0.28] |  | - | 2 | - | - |  |  |  |
|  |  |  |  |  |  |  |  |  |  |  |
| **Parameter** |  | **Total** | **Post hoc** | **I²** | **k** | **tau** | **tau²** | **Q** | **df** | **P value** |
| **TNF-α** | **Continuous** | 0.56 [0.39; 0.73] |  | 99.6% | 68 | 0.727 | 0.528 | 1.95 | 1 | 0.162 |
|  | **Intermittent** | 0.29 [-0.07; 0.65] |  | 98.9% | 18 | 0.753 | 0.568 |  |  |  |

## Sample

| **Sample** | | | | | | | | | | |
| --- | --- | --- | --- | --- | --- | --- | --- | --- | --- | --- |
| **Parameter** |  | **Total** | **Post hoc** | **I²** | **k** | **tau** | **tau²** | **Q** | **df** | **P value** |
| **IL-6** | **Plasma** | 1.21 [0.98; 1.44] |  | 99.5% | 114 | 2.249 | 5.056 | 0.61 | 1 | 0.433 |
|  | **Serum** | 1.09 [0.89; 1.29] |  | 99.6% | 73 | 0.878 | 0.772 |  |  |  |
|  |  |  |  |  |  |  |  |  |  |  |
| **Parameter** |  | **Total** | **Post hoc** | **I²** | **k** | **tau** | **tau²** | **Q** | **df** | **P value** |
| **IL-1ra** | **Plasma** | 0.52 [0.32; 0.71] | Plasma > Serum** | 99.0% | 22 | 0.45 | 0.198 | 8.32 | 1 | 0.004 |
|  | **Serum** | 0.17 [0.01; 0.34] |  | 96.9% | 9 | 0.22 | 0.048 |  |  |  |
|  |  |  |  |  |  |  |  |  |  |  |
| **Parameter** |  | **Total** | **Post hoc** | **I²** | **k** | **tau** | **tau²** | **Q** | **df** | **P value** |
| **IL-10** | **Plasma** | 0.33 [0.17; 0.48] |  | 99.7% | 41 | 0.500 | 0.250 | 1.5 | 1 | 0.220 |
|  | **Serum** | 0.55 [0.21; 0.88] |  | 99.7% | 19 | 0.714 | 0.509 |  |  |  |
|  |  |  |  |  |  |  |  |  |  |  |
| **Parameter** |  | **Total** | **Post hoc** | **I²** | **k** | **tau** | **tau²** | **Q** | **df** | **P value** |
| **IL-8** | **Plasma** | 0.84 [0.52; 1.15] | Plasma > Serum* | 99.7% | 37 | 0.965 | 0.932 | 4.48 | 1 | 0.034 |
|  | **Serum** | 0.44 [0.21; 0.67] |  | 98.6% | 12 | 0.360 | 0.129 |  |  |  |
|  |  |  |  |  |  |  |  |  |  |  |
| **Parameter** |  | **Total** | **Post hoc** | **I²** | **k** | **tau** | **tau²** | **Q** | **df** | **P value** |
| **IL-15** | **Plasma** | 0.35 [-0.06; 0.76] |  | 99.7% | 7 | 0.440 | 0.194 | 0.01 | 1 | 0.935 |
|  | **Serum** | 0.33 [-0.10; 0.76] |  | 98.4% | 5 | 0.342 | 0.117 |  |  |  |
|  |  |  |  |  |  |  |  |  |  |  |
| **Parameter** |  | **Total** | **Post hoc** | **I²** | **k** | **tau** | **tau²** | **Q** | **df** | **P value** |
| **TNF-α** | **Plasma** | 0.34 [0.22; 0.46] |  | 99.1% | 48 | 0.41 | 0.167 | 2.13 | 1 | 0.145 |
|  | **Serum** | 0.64 [0.24; 1.04] |  | 99.6% | 27 | 1.03 | 1.058 |  |  |  |

## Fasted

| **Fasted** | | | | | | | | | | |
| --- | --- | --- | --- | --- | --- | --- | --- | --- | --- | --- |
| **Parameter** |  | **Total** | **Post hoc** | **I²** | **k** | **tau** | **tau²** | **Q** | **df** | **P value** |
| **IL-6** | **Yes** | 1.21 [ 0.92; 1.49] |  | 99.2% | 88 | 2.489 | 6.195 | 0.68 | 1 | 0.410 |
|  | **No** | 1.03 [0.68; 1.37] |  | 99.7% | 37 | 1.059 | 1.121 |  |  |  |
|  |  |  |  |  |  |  |  |  |  |  |
| **Parameter** |  | **Total** | **Post hoc** | **I²** | **k** | **tau** | **tau²** | **Q** | **df** | **P value** |
| **IL-1ra** | **Yes** | 0.47 [0.12; 0.81] |  | 98.3% | 13 | 0.244 | 0.336 | 0.48 | 1 | 0.489 |
|  | **No** | 0.34 [0.15; 0.53] |  | 98.9% | 9 | 0.580 | 0.060 |  |  |  |
|  |  |  |  |  |  |  |  |  |  |  |
| **Parameter** |  | **Total** | **Post hoc** | **I²** | **k** | **tau** | **tau²** | **Q** | **df** | **P value** |
| **IL-10** | **Yes** | 0.33 [0.10; 0.57] |  | 99.5% | 25 | 0.570 | 0.324 | 0.11 | 1 | 0.738 |
|  | **No** | 0.38 [0.18; 0.59] |  | 99.7% | 18 | 0.410 | 0.168 |  |  |  |
|  |  |  |  |  |  |  |  |  |  |  |
| **Parameter** |  | **Total** | **Post hoc** | **I²** | **k** | **tau** | **tau²** | **Q** | **df** | **P value** |
| **IL-8** | **Yes** | 0.62 [0.27; 0.97] |  | 99.8% | 15 | 0.635 | 0.403 | 0.01 | 1 | 0.929 |
|  | **No** | 0.64 [0.29; 0.99] |  | 99.7% | 14 | 0.603 | 0.363 |  |  |  |
|  |  |  |  |  |  |  |  |  |  |  |
| **Parameter** |  | **Total** | **Post hoc** | **I²** | **k** | **tau** | **tau²** | **Q** | **df** | **P value** |
| **IL-15** | **Yes** | -0.00 [-0.43; 0.43] | No > Yes** | 98.7% | 5 | 0.340 | 0.116 | 7.88 | 1 | 0.005 |
|  | **No** | 0.63 [-1.46; 2.72] |  | 99.9% | 2 | 0.233 | 0.054 |  |  |  |
|  |  |  |  |  |  |  |  |  |  |  |
| **Parameter** |  | **Total** | **Post hoc** | **I²** | **k** | **tau** | **tau²** | **Q** | **df** | **P value** |
| **TNF-α** | **Yes** | 0.57 [0.27; 0.67] |  | 98.9% | 27 | 0.503 | 0.253 | 0.52 | 1 | 0.469 |
|  | **No** | 0.37 [0.14; 0.60] |  | 99.6% | 19 | 0.474 | 0.224 |  |  |  |

## Time of the day

| **Fasted** | | | | | | | | | | |
| --- | --- | --- | --- | --- | --- | --- | --- | --- | --- | --- |
| **Parameter** |  | **Total** | **Post hoc** | **I²** | **k** | **tau** | **tau²** | **Q** | **df** | **P value** |
| **IL-6** | **Morning** | 1.13 [ 0.98; 1.28] |  | 99.5% | 126 | 0.894 | 0.799 | 2.28 | 1 | 0.131 |
|  | **Afternoon** | 0.93 [0.69; 1.17] |  | 99.4% | 14 | 0.408 | 0.166 |  |  |  |
|  |  |  |  |  |  |  |  |  |  |  |
| **Parameter** |  | **Total** | **Post hoc** | **I²** | **k** | **tau** | **tau²** | **Q** | **df** | **P value** |
| **IL-1ra** | **Morning** | 0.42 [0.22; 0.62] |  | 99.1% | 22 | 0.463 | 0.215 | 2.61 | 1 | 0.106 |
|  | **Afternoon** | 0.66 [0.30; 1.03] |  | 96.8% | 4 | 0.223 | 0.050 |  |  |  |
|  |  |  |  |  |  |  |  |  |  |  |
| **Parameter** |  | **Total** | **Post hoc** | **I²** | **k** | **tau** | **tau²** | **Q** | **df** | **P value** |
| **IL-10** | **Morning** | 0.42 [0.20; 0.63] |  | 99.8% | 38 | 0.659 | 0.435 | 0.50 | 1 | 0.480 |
|  | **Afternoon** | 0.50 [0.35; 0.66] |  | 92.5% | 6 | 0.147 | 0.021 |  |  |  |
|  |  |  |  |  |  |  |  |  |  |  |
| **Parameter** |  | **Total** | **Post hoc** | **I²** | **k** | **tau** | **tau²** | **Q** | **df** | **P value** |
| **IL-8** | **Morning** | 0.50 [0.32; 0.69] | Afternoon > Morning** | 99.7% | 29 | 0.490 | 0.240 | 9.77 | 1 | 0.002 |
|  | **Afternoon** | 1.17 [0.63; 1.70] |  | 99.1% | 5 | 0.427 | 0.183 |  |  |  |
|  |  |  |  |  |  |  |  |  |  |  |
| **Parameter** |  | **Total** | **Post hoc** | **I²** | **k** | **tau** | **tau²** | **Q** | **df** | **P value** |
| **TNF-α** | **Morning** | 0.52 [0.30; 0.74] |  | 99.2% | 42 | 0.733 | 0.537 | 0.30 | 1 | 0.586 |
|  | **Afternoon** | 0.38 [-0.19; 0.95] |  | 99.7% | 6 | 0.544 | 0.296 |  |  |  |

## Age

| **AGE** | | | | | | | | | |
| --- | --- | --- | --- | --- | --- | --- | --- | --- | --- |
| **Parameter** | **Range** | **Estimate** | **se** | **tval** | **P value** | **ci.lb** | **ci.ub** | **k** | **R²** |
| **IL-6** | 19.7 - 43.6 | -0.0128 | 0.0186 | -0.6896 | 0.491 | -0.0494 | 0.0238 | 204 | 0.00% |
| **IL-1ra** | 20.6 – 43.6 | 0.0104 | 0.0118 | 0.8838 | 0.384 | -0.0137 | 0.0346 | 31 | 1.57% |
| **IL-10** | 20.3 – 43.6 | 0.0071 | 0.0140 | 0.5072 | 0.614 | -0.0208 | 0.0350 | 67 | 0.00% |
| **IL-8** | 20 - 40 | 0.0397 | 0.0186 | 2.1276 | **0.039** | 0.0022 | 0.0772 | 49 | 7.46% |
| **IL-15** | 21.21 - 32.7 | -0.0020 | 0.0403 | -0.0505 | 0.961 | -0.0919 | 0.0879 | 12 | 0.00% |
| **TNF-α** | 20 – 43.6 | -0.0041 | 0.0127 | -0.3250 | 0.746 | -0.0295 | 0.0212 | 83 | 0.00% |

## BMI

| **BMI** | | | | | | | | | |
| --- | --- | --- | --- | --- | --- | --- | --- | --- | --- |
| **Parameter** | **Range** | **Estimate** | **se** | **tval** | **P value** | **ci.lb** | **ci.ub** | **k** | **R²** |
| **IL-6** | 19.99 - 31.9 | 0.0433 | 0.0597 | 0.7262 | 0.469 | -0.0744 | 0.1610 | 183 | 0.00% |
| **IL-1ra** | 21 – 31.9 | -0.0186 | 0.0310 | -0.5995 | 0.554 | -0.0824 | 0.0452 | 28 | 0.00% |
| **IL-10** | 20.8 - 27.38 | 0.0124 | 0.0746 | 0.1660 | 0.869 | -0.1369 | 0.1617 | 62 | 0.00% |
| **IL-8** | 20.14 - 27.38 | 0.0431 | 0.0965 | 0.4465 | 0.658 | -0.1517 | 0.2379 | 44 | 0.00% |
| **IL-15** | 22 – 27.38 | 0.0511 | 0.0741 | 0.6895 | 0.506 | -0.1140 | 0.2162 | 12 | 0.00% |
| **TNF-α** | 21.34 - 31.9 | 0.0383 | 0.0452 | 0.8480 | 0.400 | -0.0517 | 0.1283 | 78 | 0.71% |

## VO_2_max

| **VO_2_MAX** | | | | | | | | | |
| --- | --- | --- | --- | --- | --- | --- | --- | --- | --- |
| **Parameter** | **Range** | **Estimate** | **se** | **tval** | **P value** | **ci.lb** | **ci.ub** | **k** | **R²** |
| **IL-6** | 33 - 73.3 | 0.0107 | 0.0141 | 0.7600 | 0.448 | -0.0171 | 0.0385 | 172 | 0.46% |
| **IL-1ra** | 33.8 - 61.61 | 0.0144 | 0.0095 | 1.5111 | 0.144 | -0.0053 | 0.0342 | 25 | 7.99% |
| **IL-10** | 33.8 - 73.3 | 0.0328 | 0.0114 | 2.8844 | **0.006** | 0.0100 | 0.0556 | 54 | 14.66% |
| **IL-8** | 33.8 - 73.3 | -0.0078 | 0.0172 | -0.4553 | 0.652 | -0.0426 | 0.0269 | 40 | 0.00% |
| **IL-15** | 45.9 - 52.9 | 0.0328 | 0.0577 | 0.5691 | 0.590 | -0.1083 | 0.1740 | 8 | 0.00% |
| **TNF-α** | 33.8 - 73.3 | -0.0001 | 0.0087 | -0.0168 | 0.987 | -0.0175 | 0.0172 | 72 | 0.00% |

## Duration

| **DURATION** | | | | | | | | | |
| --- | --- | --- | --- | --- | --- | --- | --- | --- | --- |
| **Parameter** | **Range** | **Estimate** | **se** | **tval** | **P value** | **ci.lb** | **ci.ub** | **k** | **R²** |
| **IL-6** | 20 - 1440 | 0.0031 | 0.0006 | 5.3669 | **<0.001** | 0.0019 | 0.0042 | 190 | 39.08% |
| **IL-1ra** | 22 - 258 | 0.0010 | 0.0014 | 0.7035 | 0.4876 | -0.0018 | 0.0038 | 30 | 0.45% |
| **IL-10** | 20 - 1440 | 0.0004 | 0.0004 | 1.0014 | 0.321 | -0.0004 | 0.0011 | 61 | 0.00% |
| **IL-8** | 23 - 1440 | 0.0002 | 0.0004 | 0.5617 | 0.577 | -0.0006 | 0.0011 | 46 | 0.00% |
| **IL-15** | 28 - 180 | -0.0045 | 0.0024 | -1.8333 | 0.097 | -0.0099 | 0.0010 | 12 | 23.80% |
| **TNF-α** | 20 - 1440 | 0.0004 | 0.0003 | 1.3727 | 0.174 | -0.0002 | 0.0009 | 74 | 0.66% |

## Dose

| **DOSE** | | | | | | | | | |
| --- | --- | --- | --- | --- | --- | --- | --- | --- | --- |
| **Parameter** | **Range** | **Estimate** | **se** | **tval** | **P value** | **ci.lb** | **ci.ub** | **k** | **R²** |
| **IL-6** | 118 - 6408 | 0.0008 | 0.0001 | 5.9347 | **<.0001** | 0.0005 | 0.0011 | 181 | 43.60% |
| **IL-1ra** | 134 - 1153 | 0.0003 | 0.0002 | 1.2451 | 0.2234 | -00002 | 0.0008 | 30 | 6.36% |
| **IL-10** | 129 - 4968 | 0.0001 | 0.0001 | 1.1927 | 0.2381 | -0.0001 | 0.0003 | 57 | 0.09% |
| **IL-8** | 134 - 6408 | 0.0001 | 0.0001 | 1.0379 | 0.3051 | -0.0001 | 0.0003 | 45 | 0.04% |
| **IL-15** | 183 - 970 | -0.0011 | 0.0005 | -2.1251 | 0.0625 | -0.0023 | 0.0001 | 11 | 37.94% |
| **TNF-α** | 134 - 6408 | 0.0001 | 0.0001 | 1.0307 | 0.3064 | -0.0001 | 0.0002 | 69 | 0.00% |

# Results multi variate analysis

## IL-6

| **Model** | **IL-6** | | |
| --- | --- | --- | --- |
| **INTENSITY x DURATION** | ***R^2^*** | ***b*** | ***p*** |
| **Intercept** | 38.23%*** | 1.3408 | .030 |
| **IntensityModerate** |  | -0.5167 | .421 |
| **IntensityVigorous** |  | -0.5876 | .369 |
| **Duration** |  | -0.0091 | .547 |
| **IntensityModerate:Duration** |  | 0.0122 | .423 |
| **IntensityVigorous:Duration** |  | 0.0153 | .322 |
| **ALL EXERCISE PARAMETERS** | ***R^2^*** | ***b*** | ***p*** |
| **Intercept** | 39.18%*** | 1.5160 | .043 |
| **IntensityModerate** |  | -0.5782 | .440 |
| **IntensityVigorous** |  | -0.5693 | .431 |
| **Duration** |  | -0.0103 | .521 |
| **LoadIntermittent** |  | 0.1633 | .532 |
| **ExerciseRunning** |  | -0.3574 | .066 |
| **IntensityModerate:Duration** |  | 0.0136 | .400 |
| **IntensityVigorous:Duration** |  | 0.0162 | .318 |
| **ALL SIG. MODERATORS** | ***R^2^*** | ***b*** | ***p*** |
| **Intercept** | 40.15%*** | 0.8809 | <.001 |
| **Duration** |  | 0.0031 | <.001 |
| **Sexm** |  | 0.1201 | 0.623 |
| **Sexmf** |  | -0.4101 | .203 |

## IL-10

| **Model** | **IL-10** | | |
| --- | --- | --- | --- |
| **INTENSITY x DURATION** | ***R^2^*** | ***b*** | ***p*** |
| **Intercept** | 22.08%* | 3.4659 | .001 |
| **IntensityModerate** |  | -3.1352 | .002 |
| **IntensityVigorous** |  | -3.4159 | .001 |
| **Duration** |  | -0.0588 | .003 |
| **IntensityModerate:Duration** |  | 0.0592 | .003 |
| **IntensityVigorous:Duration** |  | 0.0652 | .001 |
| **ALL EXERCISE PARAMETERS** | ***R^2^*** | ***b*** | ***p*** |
| **Intercept** | 23.54%* | 2.5817 | .016 |
| **IntensityModerate** |  | -2.3767 | .023 |
| **IntensityVigorous** |  | -2.8220 | .007 |
| **Duration** |  | -0.0435 | .035 |
| **LoadIntermittent** |  | 0.1220 | .585 |
| **ExerciseRunning** |  | 0.2896 | .083 |
| **IntensityModerate:Duration** |  | 0.0437 | .034 |
| **IntensityVigorous:Duration** |  | 0.0514 | .013 |
| **ALL SIG. MODERATORS** | ***R^2^*** | ***b*** | ***p*** |
| **Intercept** | 22.76%* | -0.9846 | .105 |
| **ExerciseRunning** |  | 0.1134 | .514 |
| **Sexm** |  | 0.2805 | .250 |
| **Sexmf** |  | 0.1420 | .625 |
| **RoBLow** |  | -0.5464 | .030 |
| **RoBModerate** |  | -0.0206 | .927 |
| **RoBSerious** |  | -0.0886 | .663 |
| **Vo2max** |  | 0.0246 | .070 |

## TNF-α

| **Model** | **TNF-α** | | |
| --- | --- | --- | --- |
| **INTENSITY x DURATION** | ***R^2^*** | ***b*** | ***p*** |
| **Intercept** | 0.00% | -0.6289 | .300 |
| **IntensityMaximal** |  | 1.1779 | .146 |
| **IntensityModerate** |  | 1.0818 | .083 |
| **IntensityVigorous** |  | 0.8727 | .146 |
| **Duration** |  | 0.0011 | .656 |
| **IntensityModerate:Duration** |  | -0.0052 | .673 |
| **IntensityVigorous:Duration** |  | -0.0008 | .738 |
| **ALL EXERCISE PARAMETERS** | ***R^2^*** | ***b*** | ***p*** |
| **Intercept** | 0.49% | -0.5559 | .369 |
| **IntensityMaximal** |  | 1.7551 | .067 |
| **IntensityModerate** |  | 1.0116 | .111 |
| **IntensityVigorous** |  | 0.9270 | .131 |
| **Duration** |  | -0.0001 | .974 |
| **LoadIntermittent** |  | -0.3858 | .101 |
| **ExerciseRunning** |  | 0.0786 | .614 |
| **IntensityModerate:Duration** |  | -0.0156 | .306 |
| **IntensityVigorous:Duration** |  | 0.0003 | .910 |
| **ALL SIG. MODERATORS** | ***R^2^*** | ***b*** | ***p*** |
| **Intercept** | 5.14% | 0.3549 | .037 |
| **ExerciseRunning** |  | 0.1728 | .254 |
| **RoBLow** |  | -0.2589 | .252 |
| **RoBModerate** |  | 0.1074 | .590 |
| **RoBSerious** |  | 0.1952 | .339 |

## IL-1ra

| **Model** | **IL-1ra** | | |
| --- | --- | --- | --- |
| **INTENSITY x DURATION** | ***R^2^*** | ***b*** | ***p*** |
| **Intercept** | 17.35% | 0.7219 | .384 |
| **IntensityModerate** |  | -0.3629 | .666 |
| **IntensityVigorous** |  | -0.5368 | .527 |
| **Duration** |  | -0.0101 | .598 |
| **IntensityModerate:Duration** |  | 0.0095 | .619 |
| **IntensityVigorous:Duration** |  | 0.0153 | .429 |
| **ALL EXERCISE PARAMETERS** | ***R^2^*** | ***b*** | ***p*** |
| **Intercept** | 28.58% | 0.8240 | .337 |
| **IntensityModerate** |  | -0.5693 | .503 |
| **IntensityVigorous** |  | -0.7574 | .365 |
| **Duration** |  | -0.0143 | .458 |
| **LoadIntermittent** |  | -0.2481 | .235 |
| **ExerciseRunning** |  | 0.2767 | .052 |
| **IntensityModerate:Duration** |  | 0.0130 | .500 |
| **IntensityVigorous:Duration** |  | 0.0186 | .335 |
| **ALL SIG. MODERATORS** | ***R^2^*** | ***b*** | ***p*** |
| **Intercept** | 9.39% | 0.2019 | .439 |
| **ExerciseRunning** |  | 0.0153 | .947 |
| **RoBLow** |  | 0.3768 | .355 |
| **RoBModerate** |  | 0.2573 | .451 |
| **RoBSerious** |  | 0.3958 | .146 |
| **SampleSerum** |  | -0.1550 | .481 |
| **Experiencetrained** |  | 0.0135 | .969 |
| **Experienceuntrained** |  | -0.2023 | .567 |

## IL-8

| **Model** | **IL-8** | | |
| --- | --- | --- | --- |
| **INTENSITY x DURATION** | ***R^2^*** | ***b*** | ***p*** |
| **Intercept** | 23.49%* | 1.9910 | .054 |
| **IntensityModerate** |  | -1.6558 | .114 |
| **IntensityVigorous** |  | -2.0488 | .060 |
| **Duration** |  | -0.0281 | .287 |
| **IntensityModerate:Duration** |  | 0.0285 | .281 |
| **IntensityVigorous:Duration** |  | 0.0388 | .147 |
| **ALL EXERCISE PARAMETERS** | ***R^2^*** | ***b*** | ***p*** |
| **Intercept** | 19.18% | 1.9701 | .181 |
| **IntensityModerate** |  | -1.6517 | .246 |
| **IntensityVigorous** |  | -2.0631 | .140 |
| **Duration** |  | -0.0281 | .410 |
| **LoadIntermittent** |  | -0.0403 | .920 |
| **ExerciseRunning** |  | 0.0550 | .836 |
| **IntensityModerate:Duration** |  | 0.0285 | .403 |
| **IntensityVigorous:Duration** |  | 0.0391 | .246 |
| **ALL SIG. MODERATORS** | ***R^2^*** | ***b*** | ***p*** |
| **Intercept** | 0.00% | 1.2344 | .013 |
| **ExerciseRunning** |  | -0.2144 | .495 |
| **RoBLow** |  | 0.3396 | .591 |
| **RoBModerate** |  | -0.3670 | .324 |
| **RoBSerious** |  | -0.0873 | .838 |
| **SampleSerum** |  | -0.5880 | .178 |
| **Experiencetrained** |  | 0.0619 | .882 |
| **Experienceuntrained** |  | -0.6094 | .212 |

## IL-15

| **Model** | **IL-15** | | |
| --- | --- | --- | --- |
| **INTENSITY x DURATION** | ***R^2^*** | ***b*** | ***p*** |
| **Intercept** | 11.21% | 0.9481 | .281 |
| **IntensityModerate** |  | -0.6535 | .475 |
| **IntensityVigorous** |  | -0.0396 | .913 |
| **Duration** |  | -0.0079 | .692 |
| **IntensityModerate:Duration** |  | 0.0056 | .783 |
| **ALL EXERCISE PARAMETERS** | ***R^2^*** | ***b*** | ***p*** |
| **Intercept** | 66.19% | 1.0227 | .045 |
| **IntensityModerate** |  | 0.2509 | .575 |
| **IntensityVigorous** |  | -0.0397 | .869 |
| **Duration** |  | -0.0090 | .053 |
| **LoadIntermittent** |  | -1.0017 | .079 |
| **ExerciseRunning** |  | -0.0317 | .916 |

# Results sensitivity analysis

For IL-6 and IL-1ra, time of day emerged as a significant moderator only in the sensitivity analysis. A significant difference between training status was observed for IL-8; however, this effect was not sustained for IL-1ra, where no significant differences between trained and untrained individuals were detected in the sensitivity analysis. Furthermore, risk of bias was no longer a significant moderator for IL-10, and comparisons between low and critical risk of bias groups for IL-1ra also lost significance. Exercise modality ceased to be a significant moderator for TNF-α. Additionally, age did not significantly moderate IL-8 responses, and sex no longer significantly influenced IL-15 responses following acute endurance exercise.

| **RISK OF BIAS** | | | | | | | | | | |
| --- | --- | --- | --- | --- | --- | --- | --- | --- | --- | --- |
| **Parameter** |  | **Total** | **Post hoc** | **I²** | **k** | **tau** | **tau²** | **Q** | **df** | **P value** |
| **IL-10**  **(Coeff.: 0.90)** | **low** | -0.01 [-0.36; 0.35] |  | 99.9% | 11 | 0.530 | 0.281 | 7.74 | 3 | 0.052 |
|  | **moderate** | 0.60 [0.23; 0.98] |  | 99.9% | 18 | 0.758 | 0.575 |  |  |  |
|  | **serious** | 0.42 [0.23; 0.62] |  | 99.9% | 21 | 0.433 | 0.187 |  |  |  |
|  | **critical** | 0.40 [0.22; 0.59] |  | 99.8% | 17 | 0.355 | 0.126 |  |  |  |
|  |  |  |  |  |  |  |  |  |  |  |
| **Parameter** |  | **Total** | **Post hoc** | **I²** | **k** | **tau** | **tau²** | **Q** | **df** | **P value** |
| **IL-1ra**  **(Coeff.: 070)** | **low** | 0.31 [-0.71; 1.33] | Moderate > critical*** | 98.4% | 3 | 0.408 | 0.166 | 19.90 | 3 | 0.0002 |
|  | **moderate** | 0.47 [0.30; 0.65] |  | 97.9% | 7 | 0.195 | 0.038 |  |  |  |
|  | **serious** | 0.64 [0.31; 0.97] |  | 97.0% | 13 | 0.558 | 0.311 |  |  |  |
|  | **critical** | 0.14 [0.02; 0.26] |  | 89.1% | 8 | 0.136 | 0.018 |  |  |  |

| **SEX** | | | | | | | | | | |
| --- | --- | --- | --- | --- | --- | --- | --- | --- | --- | --- |
| **Parameter** |  | **Total** | **Post hoc** | **I²** | **k** | **tau** | **tau²** | **Q** | **df** | **P value** |
| **IL-15**  **(Coeff.: 0.90)** | **m** | 0.18 [-0.28; 0.63] |  | 99.7% | 6 | 0.433 | 0.187 | 3.49 | 1 | 0.062 |
|  | **mf** | 0.54 [0.32; 0.76] |  | 99.9% | 5 | 0.176 | 0.031 |  |  |  |

| **EXPERIENCE** | | | | | | | | | | |
| --- | --- | --- | --- | --- | --- | --- | --- | --- | --- | --- |
| **Parameter** |  | **Total** | **Post hoc** | **I²** | **k** | **tau** | **tau²** | **Q** | **df** | **P value** |
| **IL-1ra**  **(coeff.: 0.90)** | **trained** | 0.50 [0.25; 0.75] | Trained > active** | 99.6% | 16 | 0.465 | 0.216 | 11.40 | 2 | 0.003 |
|  | **active** | 0.09 [-0.07; 0.25] |  | 95.2% | 4 | 0.100 | 0.010 |  |  |  |
|  | **untrained** | 0.24 [0.02; 0.45] |  | 99.7% | 7 | 0.234 | 0.055 |  |  |  |
|  |  |  |  |  |  |  |  |  |  |  |
| **Parameter** |  | **Total** | **Post hoc** | **I²** | **k** | **tau** | **tau²** | **Q** | **df** | **P value** |
| **IL-8**  **(coeff.: 0.90)** | **trained** | 0.79 [0.45; 1.13] | Trained > untrained* | 99.9% | 24 | 0.806 | 0.650 | 7.01 | 2 | 0.030 |
|  | **active** | 0.57 [0.17; 0.98] |  | 99.2% | 11 | 0.604 | 0.365 |  |  |  |
|  | **untrained** | 0.27 [-0.01; 0.55] |  | 99.7% | 8 | 0.332 | 0.111 |  |  |  |

| **EXERCISE TYPE** | | | | | | | | | | |
| --- | --- | --- | --- | --- | --- | --- | --- | --- | --- | --- |
| **Parameter** |  | **Total** | **Post hoc** | **I²** | **k** | **tau** | **tau²** | **Q** | **df** | **P value** |
| **TNF-α**  **(coeff.: 0.70)** | **Cycling** | 0.37 [0.22; 0.53] |  | 99.3% | 37 | 0.468 | 0.219 | 3.67 | 1 | 0.556 |
|  | **Running** | 0.68 [0.40; 0.95] |  | 98.1% | 46 | 0.981 | 0.963 |  |  |  |

| **TIME OF DAY** | | | | | | | | | | |
| --- | --- | --- | --- | --- | --- | --- | --- | --- | --- | --- |
| **Parameter** |  | **Total** | **Post hoc** | **I²** | **k** | **tau** | **tau²** | **Q** | **df** | **P value** |
| **IL-6**  **(coeff.: 0.90)** | **Morning** | 0.96 [0.83; 1.08] | Morning > Afternoon** | 99.9% | 126 | 0.735 | 0.540 | 6.59 | 1 | 0.010 |
|  | **Afternoon** | 0.69 [0.52; 0.86] |  | 99.8% | 14 | 0.296 | 0.088 |  |  |  |
|  |  |  |  |  |  |  |  |  |  |  |
| **Parameter** |  | **Total** | **Post hoc** | **I²** | **k** | **tau** | **tau²** | **Q** | **df** | **P value** |
| **IL-1ra**  **(coeff.: 0.70)** | **Morning** | 0.44 [0.23; 0.64] | Afternoon > Morning* | 98.0% | 22 | 0.481 | 0.232 | 3.99 | 1 | 0.046 |
|  | **Afternoon** | 0.73 [0.38; 1.09] |  | 91.6% | 4 | 0.206 | 0.042 |  |  |  |

| **AGE** | | | | | | | | | |
| --- | --- | --- | --- | --- | --- | --- | --- | --- | --- |
| **Parameter** | **Range** | **Estimate** | **se** | **tval** | **P value** | **ci.lb** | **ci.ub** | **k** | **R²** |
| **IL-8**  **(coeff.: 0.90)** |  | 0.0282 | 0.0150 | 1.8762 | 0.067 | -0.0020 | 0.0584 | 49 | 4.88% |

# Prisma Checklist 2020

| **Section and Topic** | **Item #** | **Checklist item** | **Location where item is reported** |
| --- | --- | --- | --- |
| **TITLE** | | |  |
| Title | 1 | Identify the report as a systematic review. | Page 1 |
| **ABSTRACT** | | |  |
| Abstract | 2 | See the PRISMA 2020 for Abstracts checklist. | Page 2 |
| **INTRODUCTION** | | |  |
| Rationale | 3 | Describe the rationale for the review in the context of existing knowledge. | 1 |
| Objectives | 4 | Provide an explicit statement of the objective(s) or question(s) the review addresses. | 1 |
| **METHODS** | | |  |
| Eligibility criteria | 5 | Specify the inclusion and exclusion criteria for the review and how studies were grouped for the syntheses. | 2.2 |
| Information sources | 6 | Specify all databases. registers. websites. organisations. reference lists and other sources searched or consulted to identify studies. Specify the date when each source was last searched or consulted. | 2.1 |
| Search strategy | 7 | Present the full search strategies for all databases. registers and websites. including any filters and limits used. | 2.1/Prospero |
| Selection process | 8 | Specify the methods used to decide whether a study met the inclusion criteria of the review. including how many reviewers screened each record and each report retrieved. whether they worked independently. and if applicable. details of automation tools used in the process. | 2.3 |
| Data collection process | 9 | Specify the methods used to collect data from reports. including how many reviewers collected data from each report. whether they worked independently. any processes for obtaining or confirming data from study investigators. and if applicable. details of automation tools used in the process. | 2.4 |
| Data items | 10a | List and define all outcomes for which data were sought. Specify whether all results that were compatible with each outcome domain in each study were sought (e.g. for all measures. time points. analyses). and if not. the methods used to decide which results to collect. | 2.4 |
|  | 10b | List and define all other variables for which data were sought (e.g. participant and intervention characteristics. funding sources). Describe any assumptions made about any missing or unclear information. | 2.4 |
| Study risk of bias assessment | 11 | Specify the methods used to assess risk of bias in the included studies. including details of the tool(s) used. how many reviewers assessed each study and whether they worked independently. and if applicable. details of automation tools used in the process. | 2.5 |
| Effect measures | 12 | Specify for each outcome the effect measure(s) (e.g. risk ratio. mean difference) used in the synthesis or presentation of results. | 2.6 |
| Synthesis methods | 13a | Describe the processes used to decide which studies were eligible for each synthesis (e.g. tabulating the study intervention characteristics and comparing against the planned groups for each synthesis (item #5)). | 2.6 |
|  | 13b | Describe any methods required to prepare the data for presentation or synthesis. such as handling of missing summary statistics. or data conversions. | 2.4. 2.6 |
|  | 13c | Describe any methods used to tabulate or visually display results of individual studies and syntheses. | 2.6. 2.7 |
|  | 13d | Describe any methods used to synthesize results and provide a rationale for the choice(s). If meta-analysis was performed. describe the model(s). method(s) to identify the presence and extent of statistical heterogeneity. and software package(s) used. | 2.6. 2.7 |
|  | 13e | Describe any methods used to explore possible causes of heterogeneity among study results (e.g. subgroup analysis. meta-regression). | 2.8 |
|  | 13f | Describe any sensitivity analyses conducted to assess robustness of the synthesized results. | 2.6 |
| Reporting bias assessment | 14 | Describe any methods used to assess risk of bias due to missing results in a synthesis (arising from reporting biases). | 2.9 |
| Certainty assessment | 15 | Describe any methods used to assess certainty (or confidence) in the body of evidence for an outcome. | 2.9 |
| **RESULTS** | | |  |
| Study selection | 16a | Describe the results of the search and selection process. from the number of records identified in the search to the number of studies included in the review. ideally using a flow diagram. | 3.1 |
|  | 16b | Cite studies that might appear to meet the inclusion criteria. but which were excluded. and explain why they were excluded. | 3.1 |
| Study characteristics | 17 | Cite each included study and present its characteristics. | 3.2 |
| Risk of bias in studies | 18 | Present assessments of risk of bias for each included study. | 3.3 |
| Results of individual studies | 19 | For all outcomes. present. for each study: (a) summary statistics for each group (where appropriate) and (b) an effect estimate and its precision (e.g. confidence/credible interval). ideally using structured tables or plots. | 3.4 |
| Results of syntheses | 20a | For each synthesis. briefly summarise the characteristics and risk of bias among contributing studies. |  |
|  | 20b | Present results of all statistical syntheses conducted. If meta-analysis was done. present for each the summary estimate and its precision (e.g. confidence/credible interval) and measures of statistical heterogeneity. If comparing groups. describe the direction of the effect. | 3.4. Fig. 4. Table S4 |
|  | 20c | Present results of all investigations of possible causes of heterogeneity among study results. | 3.4. Fig. 4. Table S4 |
|  | 20d | Present results of all sensitivity analyses conducted to assess the robustness of the synthesized results. | 2.6 |
| Reporting biases | 21 | Present assessments of risk of bias due to missing results (arising from reporting biases) for each synthesis assessed. | Table S4 |
| Certainty of evidence | 22 | Present assessments of certainty (or confidence) in the body of evidence for each outcome assessed. | 2.10. Table S5 |
| **DISCUSSION** | | |  |
| Discussion | 23a | Provide a general interpretation of the results in the context of other evidence. | 4.1. 4.2 |
|  | 23b | Discuss any limitations of the evidence included in the review. | 4.3 |
|  | 23c | Discuss any limitations of the review processes used. | 4.3 |
|  | 23d | Discuss implications of the results for practice. policy. and future research. | 4.3 |
| **OTHER INFORMATION** | | |  |
| Registration and protocol | 24a | Provide registration information for the review. including register name and registration number. or state that the review was not registered. | 2 |
|  | 24b | Indicate where the review protocol can be accessed. or state that a protocol was not prepared. | 2 |
|  | 24c | Describe and explain any amendments to information provided at registration or in the protocol. | 2 |
| Support | 25 | Describe sources of financial or non-financial support for the review. and the role of the funders or sponsors in the review. | 6 |
| Competing interests | 26 | Declare any competing interests of review authors. | 6 |
| Availability of data. code and other materials | 27 | Report which of the following are publicly available and where they can be found: template data collection forms; data extracted from included studies; data used for all analyses; analytic code; any other materials used in the review. | 2.7 |

# References

1. Skinner S, Nader E, Stauffer E, Robert M, Boisson C, Cibiel A, et al. Differential impacts of trail and ultra-trail running on cytokine profiles: An observational study. Clin Hemorheol Microcirc. 2021;78(3):301–10.

2. Tamura Y, Watanabe K, Kantani T, Hayashi J, Ishida N, Kaneki M. Upregulation of circulating IL-15 by treadmill running in healthy individuals: Is IL-15 an endocrine mediator of the beneficial effects of endurance exercise? Endocr J. 2011;58(3):211–5.

3. Ueda N, Musashi M, Shimoda T, Kawaguchi Y, Ohkubo I, Nakagawa Y. Involvement of G‐CSF, IL‐6, and cortisol in transient neutrophilia after marathon races. Eur J Haematol. 2021;107(5):583–91.

4. Faraldi M, Sansoni V, Perego S, Gomarasca M, Gerosa L, Ponzetti M, et al. Acute changes in free and extracellular vesicle-associated circulating miRNAs and myokine profile in professional sky-runners during the Gran Sasso d’Italia vertical run. Front Mol Biosci. 2022;9:915080.

5. Li D, Wang P, Wei W, Wang C, Zhong Y, Lv L, et al. Serum MicroRNA Expression Patterns in Subjects After the 5-km Exercise Are Strongly Associated With Cardiovascular Adaptation. Front Physiol. 2021;12:755656.

6. Comassi M, Vitolo E, Pratali L, Del Turco S, Dellanoce C, Rossi C, et al. Acute effects of different degrees of ultra‐endurance exercise on systemic inflammatory responses. Intern Med J. 2015;45(1):74–9.

7. Weimann A, Lun A, Lun S, Zimmermann M, Borges AC, Ziebig R, et al. Leukocyte, neutrophil, immature granulocyte counts and interleukin-6 are superior to procalcitonin, C-reactive protein and delta-He for detection of mild inflammation: data from marathon runners producing mild systemic inflammation visible immediately after the run. LaboratoriumsMedizin. 2010;34(1):53–9.

8. Peeling P, Dawson B, Goodman C, Landers G, Wiegerinck ET, Swinkels DW, et al. Training Surface and Intensity. Med Sci Sports Exerc. 2009;41(5):1138–45.

9. Larsen EL, Poulsen HE, Michaelsen C, Kjær LK, Lyngbæk M, Andersen ES, et al. Differential time responses in inflammatory and oxidative stress markers after a marathon: An observational study. J Sports Sci. 2020;38(18):2080–91.

10. Ostapiuk-Karolczuk J, Zembron-Lacny A, Naczk M, Gajewski M, Kasperska A, Dziewiecka H, et al. Cytokines and cellular inflammatory sequence in non-athletes after prolonged exercise. J Sports Med Phys Fitness. 2012;52(5):563—568.

11. Badenhorst CE, Dawson B, Goodman C, Sim M, Cox GR, Gore CJ, et al. Influence of post-exercise hypoxic exposure on hepcidin response in athletes. Eur J Appl Physiol. 2014;114(5):951–9.

12. Landers-Ramos RQ, Jenkins NT, Spangenburg EE, Hagberg JM, Prior SJ. Circulating angiogenic and inflammatory cytokine responses to acute aerobic exercise in trained and sedentary young men. Eur J Appl Physiol. 2014;114(7):1377–84.

13. Sanderson M, McKinlay BJ, Theocharidis A, Kouvelioti R, Falk B, Klentrou P. Changes in Inflammatory Cytokines and Irisin in Response to High Intensity Swimming in Adolescent versus Adult Male Swimmers. Sports. 2020;8(12):157.

14. Stacey DL, Gibala MJ, Martin Ginis KA, Timmons BW. Effects of Recovery Method After Exercise on Performance, Immune Changes, and Psychological Outcomes. Journal of Orthopaedic & Sports Physical Therapy. 2010;40(10):656–65.

15. Kimura H, Suzui M, Nagao F, Matsumoto K. Highly Sensitive Determination of Plasma Cytokines by Time-Resolved Fluoroimmunoassay; Effect of Bicycle Exercise on Plasma Level of Interleukin-1 (IL-1), Tumor Necrosis Factor a (TNF a), and Interferon γ (IFN γ). Analytical Sciences. 2001;17(5):593–7.

16. Kraemer WJ, Fragala MS, Watson G, Volek JS, Rubin MR, French DN, et al. Hormonal responses to a 160-km race across frozen Alaska. Br J Sports Med. 2008;42(2):116–20.

17. Vassalle C, Piaggi P, Weltman N, Prontera C, Garbella E, Menicucci D, et al. Innovative Approach to Interpret the Variability of Biomarkers After Ultra-Endurance Exercise: The Multifactorial Analysis. Biomark Med. 2014;8(6):881–91.

18. Baygutalp F, Buzdağlı Y, Ozan M, Koz M, Kılıç Baygutalp N, Atasever G. Impacts of different intensities of exercise on inflammation and hypoxia markers in low altitude. BMC Sports Sci Med Rehabil. 2021;13(1):145.

19. Santos VC, Levada‐Pires AC, Alves SR, Pithon‐Curi TC, Curi R, Cury‐Boaventura MF. Changes in lymphocyte and neutrophil function induced by a marathon race. Cell Biochem Funct. 2013 Apr 13;31(3):237–43.

20. Jeukendrup AE, Vet-Joop K, Sturk A, Stegen JHJC, Senden J, Saris WHM, et al. Relationship between gastro-intestinal complaints and endotoxaemia, cytokine release and the acute-phase reaction during and after a long-distance triathlon in highly trained men. Clin Sci. 2000;98(1):47–55.

21. Suzuki K, Nakaji S, Yamada M, Liu Q, Kurakake S, Okamura N, et al. Impact of a Competitive Marathon Race on Systemic Cytokine and Neutrophil Responses. Med Sci Sports Exerc. 2003;35(2):348–55.

22. Brestoff JR, Clippinger B, Spinella T, von Duvillard SP, Nindl B, Arciero PJ. An acute bout of endurance exercise but not sprint interval exercise enhances insulin sensitivity. Applied Physiology, Nutrition, and Metabolism. 2009;34(1):25–32.

23. Drenth JP, Van Uum SH, Van Deuren M, Pesman GJ, Van der Ven-Jongekrijg J, Van der Meer JW. Endurance run increases circulating IL-6 and IL-1ra but downregulates ex vivo TNF-alpha and IL-1 beta production. J Appl Physiol. 1995;79(5):1497–503.

24. Chiu YH, Lai JI, Tseng CY, Wang SH, Li LH, Kao WF, et al. Impact of angiotension I converting enzyme gene I/D polymorphism on running performance, lipid, and biochemical parameters in ultra-marathoners. Medicine. 2019;98(29):e16476.

25. Schild M, Eichner G, Beiter T, Zügel M, Krumholz-Wagner I, Hudemann J, et al. Effects of Acute Endurance Exercise on Plasma Protein Profiles of Endurance-Trained and Untrained Individuals over Time. Mediators Inflamm. 2016;2016:1–11.

26. Santos VC, Sierra APR, Oliveira R, Caçula KG, Momesso CM, Sato FT, et al. Marathon Race Affects Neutrophil Surface Molecules: Role of Inflammatory Mediators. PLoS One. 2016;11(12):e0166687.

27. Nickel T, Emslander I, Sisic Z, David R, Schmaderer C, Marx N, et al. Modulation of dendritic cells and toll-like receptors by marathon running. Eur J Appl Physiol. 2012;112(5):1699–708.

28. Rowlands DS, Pearce E, Aboud A, Gillen JB, Gibala MJ, Donato S, et al. Oxidative stress, inflammation, and muscle soreness in an 894-km relay trail run. Eur J Appl Physiol. 2012;112(5):1839–48.

29. Ostrowski K, Schjerling P, Pedersen BK. Physical activity and plasma interleukin-6 in humans - effect of intensity of exercise. Eur J Appl Physiol. 2000;83(6):512–5.

30. Suzuki K, Yamada M, Kurakake S, Okamura N, Yamaya K, Liu Q, et al. Circulating cytokines and hormones with immunosuppressive but neutrophil-priming potentials rise after endurance exercise in humans. Eur J Appl Physiol. 2000;81(4):281–7.

31. Stelzer I, Kröpfl JM, Fuchs R, Pekovits K, Mangge H, Raggam RB, et al. Ultra‐endurance exercise induces stress and inflammation and affects circulating hematopoietic progenitor cell function. Scand J Med Sci Sports. 2015;25(5).

32. Ostrowski K, Rohde T, Zacho M, Asp S, Pedersen BK. Evidence that interleukin‐6 is produced in human skeletal muscle during prolonged running. J Physiol. 1998;508(3):949–53.

33. Ostrowski K, Rohde T, Asp S, Schjerling P, Pedersen BK. Pro‐ and anti‐inflammatory cytokine balance in strenuous exercise in humans. J Physiol. 1999;515(1):287–91.

34. Donnikov AE, Shkurnikov MYu, Akimov EB, Grebenyuk ES, Khaustova SA, Shahmatova EM, et al. Effect of a Six-Hour Marathon Ultra-Race on the Levels of IL-6, LIF, and SCF. Bull Exp Biol Med. 2009;148(5):819–21.

35. Deetjen C, Mair J, Herold M, Wiedermann F, Hobisch-Hagen P, Jelkmann W, et al. Time Course of Neopterin and Cytokines After an Extreme Bicycle Race at Moderate Altitude. Pteridines. 1997;8(3):195–200.

36. Bekos C, Zimmermann M, Unger L, Janik S, Hacker P, Mitterbauer A, et al. Non-professional marathon running: RAGE axis and ST2 family changes in relation to open-window effect, inflammation and renal function. Sci Rep. 2016;6(1):32315.

37. Villar-Fincheira P, Paredes AJ, Hernández-Díaz T, Norambuena-Soto I, Cancino-Arenas N, Sanhueza-Olivares F, et al. Soluble Interleukin-6 Receptor Regulates Interleukin-6-Dependent Vascular Remodeling in Long-Distance Runners. Front Physiol. 2021;12:722528.

38. Philippe M, Krüsmann P, Mersa L, Eder E, Gatterer H, Melmer A, et al. Acute effects of concentric and eccentric exercise on glucose metabolism and interleukin-6 concentration in healthy males. Biol Sport. 2016;33(2):153–8.

39. Siegel AJ, Verbalis JG, Clement S, Mendelson JH, Mello NK, Adner M, et al. Hyponatremia in Marathon Runners due to Inappropriate Arginine Vasopressin Secretion. Am J Med. 2007;120(5):461.e11-461.e17.

40. Cappuccilli M, Mosconi G, Roi GS, De Fabritiis M, Totti V, Merni F, et al. Inflammatory and Adipose Response in Solid Organ Transplant Recipients After a Marathon Cycling Race. Transplant Proc. 2016;48(2):408–14.

41. Benedetti S, Gemma Nasoni M, Palma F, Citarella R, Luchetti F. Serum changes in sTWEAK and its scavenger receptor sCD163 in ultramarathon athletes running the 24-h race. Cytokine. 2021;137:155315.

42. Mendham AE, Duffield R, Marino F, Coutts AJ. Differences in the acute inflammatory and glucose regulatory responses between small-sided games and cycling in sedentary, middle-aged men. J Sci Med Sport. 2015;18(6):714–9.

43. Yargic MP, Torgutalp S, Akin S, Babayeva N, Torgutalp M, Demirel HA. Acute long-distance trail running increases serum IL-6, IL-15, and Hsp72 levels. Applied Physiology, Nutrition, and Metabolism. 2019;44(6):627–31.

44. Hew-Butler T, Noakes TD, Soldin SJ, Verbalis JG. Acute changes in endocrine and fluid balance markers during high-intensity, steady-state, and prolonged endurance running: unexpected increases in oxytocin and brain natriuretic peptide during exercise. Eur J Endocrinol. 2008;159(6):729–37.

45. Panagoulias I, Charokopos N, Thomas I, Spantidea PI, de Lastic AL, Rodi M, et al. Shifting gears: Study of immune system parameters of male habitual marathon runners. Front Immunol. 2023;13.

46. Jouffroy R, Anglicheau D, Mansencal N, Toussaint JF, Antero J. Relationship between Exercise Intensity and IL-6 Increase during an 80 km Long-Distance Running Race. Int J Environ Res Public Health. 2022;19(11):6368.

47. Bonsignore MR, Morici G, Riccioni R, Huertas A, Petrucci E, Veca M, et al. Hemopoietic and angiogenetic progenitors in healthy athletes: different responses to endurance and maximal exercise. J Appl Physiol. 2010;109(1):60–7.

48. Cairns RS, Hew-Butler T. Incidence of Exercise-Associated Hyponatremia and Its Association With Nonosmotic Stimuli of Arginine Vasopressin in the GNW100s Ultra-endurance Marathon. Clinical Journal of Sport Medicine. 2015;25(4):347–54.

49. Jee H, Jin Y. Effects of Prolonged Endurance Exercise on Vascular Endothelial and Inflammation Markers. J Sports Sci Med. 2012;11:719–26.

50. Arakawa K, Hosono A, Shibata K, Ghadimi R, Fuku M, Goto C, et al. Changes in blood biochemical markers before, during, and after a 2-day ultramarathon. Open Access J Sports Med. 2016;43–50.

51. Catoire M, Mensink M, Kalkhoven E, Schrauwen P, Kersten S. Identification of human exercise-induced myokines using secretome analysis. Physiol Genomics. 2014 Apr 1;46(7):256–67.

52. Perry C, Pick M, Bdolach N, Hazan-Halevi I, Kay S, Berr I, et al. Endurance Exercise Diverts the Balance between Th17 Cells and Regulatory T Cells. PLoS One. 2013;8(10):e74722.

53. Mendham AE, Coutts AJ, Duffield R. The acute effects of aerobic exercise and modified rugby on inflammation and glucose homeostasis within Indigenous Australians. Eur J Appl Physiol. 2012;112(11):3787–95.

54. Steward CJ, Hill M, Menzies C, Bailey SJ, Rahman M, Thake CD, et al. Post exercise hot water immersion and hot water immersion in isolation enhance vascular, blood marker, and perceptual responses when compared to exercise alone. Scand J Med Sci Sports. 2024;34(3):e14600.

55. Vaisberg M, Suguri VM, Gregorio LC, Lopes JD, Bachi ALL. Cytokine kinetics in nasal mucosa and sera: new insights in understanding upper-airway disease of marathon runners. Exerc Immunol Rev. 2013;19:49–59.

56. Nieman DC, Henson DA, Dumke CL, Oley K, McAnulty SR, Davis JM, et al. Ibuprofen use, endotoxemia, inflammation, and plasma cytokines during ultramarathon competition. Brain Behav Immun. 2006;20(6):578–84.

57. Sprenger H, Jacobs C, Nain M, Gressner AM, Prinz H, Wesemann W, et al. Enhanced release of cytokines, interleukin-2 receptors, and neopterin after long-distance running. Clin Immunol Immunopathol. 1992;63(2):188–95.

58. Jürimäe J, Purge P, Tillmann V. Serum sclerostin and cytokine responses to prolonged sculling exercise in highly-trained male rowers. J Sports Sci. 2021;39(5):591–7.

59. Hayashi N, Ishibashi A, Goto K. Effects of diet before endurance exercise on hepcidin response in young untrained females. J Exerc Nutrition Biochem. 2018;22(4):55–61.

60. Nieman D, Henson D, Gojanovich G, Davis J, Dumke C, Utter A, et al. Immune Changes: 2 h of Continuous vs. Intermittent Cycling. Int J Sports Med. 2007;28(7):625–30.

61. Yamada M, Suzuki K, Kudo S, Totsuka M, Nakaji S, Sugawara K. Raised plasma G-CSF and IL-6 after exercise may play a role in neutrophil mobilization into the circulation. J Appl Physiol. 2002;92(5):1789–94.

62. Siegel AJ, Verbalis JG, Clement S, Mendelson JH, Mello NK, Adner M, et al. Hyponatremia in Marathon Runners due to Inappropriate Arginine Vasopressin Secretion. Am J Med. 2007;120(5):461.e11-461.e17.

63. Mucci P, Durand F, Lebel B, Bousquet J, Préfaut C. Interleukins 1-beta, -8, and histamine increases in highly trained, exercising athletes. Med Sci Sports Exerc. 2000;32(6):1094–100.

64. Stajer V, Vranes M, Ostojic SM. Correlation between biomarkers of creatine metabolism and serum indicators of peripheral muscle fatigue during exhaustive exercise in active men. Research in Sports Medicine. 2020;28(1):147–54.

65. Kon M, Ebi Y, Nakagaki K. Effects of a single bout of high-intensity interval exercise on C1q/TNF-related proteins. Applied Physiology, Nutrition, and Metabolism. 2019;44(1):47–51.

66. Mohamed S, Lamya N, Hamda M. Effect of Maximal Versus Supra-Maximal Exhausting Race on Lipid Peroxidation, Antioxidant Activity and Muscle-Damage Biomarkers in Long-Distance and Middle-Distance Runners. Asian J Sports Med. 2016;7(1):e27902.

67. Steinberg JG, Ba A, Brégeon F, Delliaux St, Jammes Y. Cytokine and Oxidative Responses to Maximal Cycling Exercise in Sedentary Subjects. Med Sci Sports Exerc. 2007;39(6):964–8.

68. Said M, Feki Y, Hamza M, Machghoul S, Amri M. Effects of two kinds of exhaustive maximal exercise on pro-inflammatory cytokines concentrations in trained and untrained humans. Biol Sport. 2005;22(4).

69. Kapilevich L V., Zakharova AN, Kabachkova A V., Kironenko TA, Orlov SN. Dynamic and Static Exercises Differentially Affect Plasma Cytokine Content in Elite Endurance- and Strength-Trained Athletes and Untrained Volunteers. Front Physiol. 2017;8:35.

70. Rhibi F, Zouhal H, Santos Lira F, Ouerghi N, Prioux J, Besbes S, et al. Inflammatory cytokines and metabolic responses to high-intensity intermittent training: effect of the exercise intensity. Biol Sport. 2022;39(2):263–72.

71. Kröpfl JM, Stelzer I, Mangge H, Pekovits K, Fuchs R, Allard N, et al. Exercise-Induced Norepinephrine Decreases Circulating Hematopoietic Stem and Progenitor Cell Colony-Forming Capacity. PLoS One. 2014;9(9):e106120.

72. Silva-Cavalcante MD, Couto PG, Azevedo R de A, Gáspari AF, Coelho DB, Lima-Silva AE, et al. Stretch–shortening cycle exercise produces acute and prolonged impairments on endurance performance: is the peripheral fatigue a single answer? Eur J Appl Physiol. 2019;119(7):1479–89.

73. Rivier A, Péne J, Chanez P, Anselme F, Caillaud C, Préfaut C, et al. Release of Cytokines by Blood Monocytes During Strenuous Exercise. Int J Sports Med. 1994;15(4):192–8.

74. Kapilevich L V., Zakharova AN, Kabachkova A V., Kironenko TA, Dyakova EYu, Orlov SN. Changes in the plasma levels of myokines after different physical exercises in athletes and untrained individuals. Hum Physiol. 2017;43(3):312–9.

75. Mendez‐Gutierrez A, Aguilera CM, Osuna‐Prieto FJ, Martinez‐Tellez B, Rico Prados MC, Acosta FM, et al. Exercise‐induced changes on exerkines that might influence brown adipose tissue metabolism in young sedentary adults. Eur J Sport Sci. 2023;23(4):625–36.

76. Cabral-Santos C, Castrillón CIM, Miranda RAT, Monteiro PA, Inoue DS, Campos EZ, et al. Inflammatory Cytokines and BDNF Response to High-Intensity Intermittent Exercise: Effect the Exercise Volume. Front Physiol. 2016;7:509.

77. Skarpańska-Stejnborn A, Basta P, Trzeciak J, Szcześniak-Pilaczyńska Ł. Effect of intense physical exercise on hepcidin levels and selected parameters of iron metabolism in rowing athletes. Eur J Appl Physiol. 2015;115(2):345–51.

78. Keohane D, Woods T, McCarthy Y, O’Connor P, Underwood S, Molloy M. A Repeated-measures Case Series of Physiological Responses to a Transoceanic Rowing Race. Int J Sports Med. 2019;40(3):152–7.

79. Żebrowska A, Jastrzębski D, Sadowska-Krępa E, Sikora M, Di Giulio C. Comparison of the Effectiveness of High-Intensity Interval Training in Hypoxia and Normoxia in Healthy Male Volunteers: A Pilot Study. Biomed Res Int. 2019;2019:1–10.

80. Möbius-Winkler S, Hilberg T, Menzel K, Golla E, Burman A, Schuler G, et al. Time-dependent mobilization of circulating progenitor cells during strenuous exercise in healthy individuals. J Appl Physiol. 2009;107(6):1943–50.

81. Reichel T, Held S, Schwarz A, Hacker S, Wesemann F, Donath L, et al. Acute response of biomarkers in plasma from capillary blood after a strenuous endurance exercise bout. Eur J Appl Physiol. 2023;123(1):179–89.

82. Peeling P, Fulton S, Sim M, White J. Recovery Effects of Hyperoxic Gas Inhalation Or Contrast Water Immersion on the Postexercise Cytokine Response, Perceptual Recovery, and Next Day Exercise Performance. J Strength Cond Res. 2012;26(4):968–75.

83. Northoff H, Symons S, Zieker D, Schaible E V, Schäfer K, Thoma S, et al. Gender- and menstrual phase dependent regulation of inflammatory gene expression in response to aerobic exercise. Exerc Immunol Rev. 2008;14:86—103.

84. Sugama K, Suzuki K, Yoshitani K, Shiraishi K, Miura S, Yoshioka H, et al. Changes of thioredoxin, oxidative stress markers, inflammation and muscle/renal damage following intensive endurance exercise. Exerc Immunol Rev. 2015;21:130–42.

85. Cox AJ, Pyne DB, Gleeson M, Callister R. Relationship between C-reactive protein concentration and cytokine responses to exercise in healthy and illness-prone runners. Eur J Appl Physiol. 2009;107(5):611–4.

86. Snipe RMJ, Khoo A, Kitic CM, Gibson PR, Costa RJS. The impact of exertional-heat stress on gastrointestinal integrity, gastrointestinal symptoms, systemic endotoxin and cytokine profile. Eur J Appl Physiol. 2018;118(2):389–400.

87. Crabb EB, Franco RL, Caslin HL, Blanks AM, Bowen MK, Acevedo EO. The effect of acute physical and mental stress on soluble cellular adhesion molecule concentration. Life Sci. 2016;157:91–6.

88. Żebrowska A, Głuchowska B, Jastrzębski D, Kochańska-Dziurowicz A, Stanjek-Cichoracka A, Pokora I. Endurance Training and the Risk of Bronchial Asthma in Female Cross-Country Skiers. In: Body Metabolism and Exercise. Springer International Publishing; 2014. p. 29–34.

89. Blumkaitis JC, Nunes N, Strepp T, Tomaskovic A, Wenger M, Widauer H, et al. Exploring sex differences in blood-based biomarkers following exhaustive exercise using bioinformatics analysis. Biol Sport. 2024;41(3):105–18.

90. Humińska-Lisowska K, Mieszkowski J, Kochanowicz A, Bojarczuk A, Niespodziński B, Brzezińska P, et al. Implications of Adipose Tissue Content for Changes in Serum Levels of Exercise-Induced Adipokines: A Quasi-Experimental Study. Int J Environ Res Public Health. 2022;19(14):8782.

91. Reihmane D, Jurka A, Tretjakovs P. The Relationship Between Maximal Exercise‐Induced Increases in Serum IL‐6, MPO and MMP‐9 Concentrations. Scand J Immunol. 2012;76(2):188–92.

92. Dogru Y, Varol SR, Rudarli Nalcakan G, Akyuz M, Tas M, Ulman C. Effects of eccentric exercise-induced delayed onset muscle soreness on endoplasmic reticulum stress-related markers. Turkish Journal of Biochemistry. 2021;46(4):407–14.

93. Keller C, Steensberg A, Pilegaard H, Osada T, Saltin B, Pedersen BK, et al. Transcriptional activation of the IL‐6 gene in human contracting skeletal muscle: influence of muscle glycogen content. The FASEB Journal. 2001;15(14):1–15.

94. Limprasertkul A, You T, Fisher NM, Awad AB, Pendergast DR. Exercise-induced plasma cytokines between older and younger adults : original research article. International SportMed Journal. 2013;14(1):16–28.

95. Zoladz J, Majerczak J, Duda K, Chłopicki S. Exercise-induced prostacyclin release positively correlates with VO2max in young healthy men. Physiol Res. 2009;229–38.

96. Alizadeh A, Alizadeh H. Downhill running exercise increases circulating level of myokine meteorin-like hormone in humans. J Sports Med Phys Fitness. 2022;62(5).

97. Nielsen S, Scheele C, Yfanti C, Åkerström T, Nielsen AR, Pedersen BK, et al. Muscle specific microRNAs are regulated by endurance exercise in human skeletal muscle. J Physiol. 2010;588(20):4029–37.

98. Pussieldi GA, Gomes EC, Veneroso CE, De Paz JA, Fonseca TR, Mendes TT, et al. Soluble tumour necrosis factor receptor-1 (sTNFR1) levels are positively associated with exercise intensity in athletes after strenuous off-road cycling. J Sports Med Phys Fitness. 2014;54(2):225–31.

99. Riebe D, Ehrman JK, Liguori G, Magal M. ACSM’s guidelines for exercise testing and prescription. Tenth edition. American College of Sports Medicine; 2018.

100. Acevedo OG, Aragón-Vela J, De la Cruz Márquez JC, Marín MM, Casuso RA, Huertas JR. Seawater Hydration Modulates IL-6 and Apelin Production during Triathlon Events: A Crossover Randomized Study. Int J Environ Res Public Health. 2022;19(15):9581.

101. Akerstrom T, Steensberg A, Keller P, Keller C, Penkowa M, Pedersen BK. Exercise induces interleukin-8 expression in human skeletal muscle. Journal of Physiology. 2005;563(2):507–16.

102. Aktitiz S, Atakan MM, Turnagöl HH, Koşar ŞN. Interleukin-6, undercarboxylated osteocalcin, and brain-derived neurotrophic factor responses to single and repeated sessions of high-intensity interval exercise. Peptides (NY). 2022;157:170864.

103. Alfaro-Magallanes VM, Barba-Moreno L, Rael B, Romero-Parra N, Rojo-Tirado MA, Benito PJ, et al. Hepcidin response to interval running exercise is not affected by oral contraceptive phase in endurance-trained women. Scand J Med Sci Sports. 2021;31(3):643–52.

104. Almada C, Cataldo LR, Smalley S V., Diaz E, Serrano A, Hodgson MI, et al. Plasma levels of interleukin-6 and interleukin-18 after an acute physical exercise: Relation with post-exercise energy intake in twins. J Physiol Biochem. 2013;69(1):85–95.

105. Monteiro PA, Campos EZ, de Oliveira FP, Peres FP, Rosa-Neto JC, Pimentel GD, et al. Modulation of inflammatory response arising from high-intensity intermittent and concurrent strength training in physically active males. Cytokine. 2017;91:104–9.

106. Antunes BM, Campos EZ, dos Santos RVT, Rosa-Neto JC, Franchini E, Bishop NC, et al. Anti-inflammatory response to acute exercise is related with intensity and physical fitness. J Cell Biochem. 2019;120(4):5333–42.

107. Araujo NC, Neto AMM, Fujimori M, Bortolini MS, Justino AB, Honorio-França AC, et al. Immune and Hormonal Response to High-intensity Exercise during Orienteering. Int J Sports Med. 2019;40(12):768–73.

108. Arroyo E, Laudato JA, Gibson BM, Dulaney CS, Vaughan JA, Followay BN, et al. Tumor necrosis factor-α, TNF receptor, and soluble TNF receptor responses to aerobic exercise in the heat. Cytokine X. 2020;2(3).

109. Bacurau R, Bassit R, Sawada L, Navarro F, Martinsjr E, Costarosa L. Carbohydrate supplementation during intense exercise and the immune response of cyclists. Clinical Nutrition. 2002;21(5):423–9.

110. Barba-Moreno L, Alfaro-Magallanes VM, de Jonge XAKJ, Díaz AE, Cupeiro R, Peinado AB. Hepcidin and interleukin-6 responses to endurance exercise over the menstrual cycle. Eur J Sport Sci. 2022;22(2):218–26.

111. Blegen M, Cheatham C, Caine-Bish N, Woolverton C, Marcinkiewicz J, Glickman E. The immunological and metabolic responses to exercise of varying intensities in normoxic and hypoxic environments. The Journal of Strength & Conditioning Research. 2008;22(5):1638–44.

112. Brenner IKM, Natale VM, Vasiliou P, Moldoveanu AI, Shek PN, Shephard RJ. Impact of three different types of exercise on components of the inflammatory response. Eur J Appl Physiol Occup Physiol. 1999;80:452–60.

113. Broadbent S, Rousseau JJ, Thorp RM, Choate SL, Jackson FS, Rowlands DS. Vibration therapy reduces plasma IL6 and muscle soreness after downhill running. Br J Sports Med. 2010;44(12):888–94.

114. Bruunsgaard H, Galbo H, Halkjaer-Kristensen J, Johansen TL, MacLean DA, Pedersen BK. Exercise-induced increase in serum inferleukin-6 in humans is related to muscle damage. Journal of Physiology. 1997;499(3):833–41.

115. Cabral-Santos C, Gerosa-Neto J, Sayuri Inoue D, Leme Gonçalves Panissa V, Alberto Gobbo L, Moura Zagatto A, et al. Similar Anti-Inflammatory Acute Responses from Moderate-Intensity Continu-ous and High-Intensity Intermittent Exercise. J Sports Sci Med. 2015;14:849–56.

116. Chaffin ME, Davis JE, Berg KE, French JA, Meendering JR, Llewellyn TL. Interleukin-6 and delayed onset muscle soreness do not vary during the menstrual cycle. Res Q Exerc Sport. 2011;82(4):693–701.

117. Stanley Chan MH, Carey AL, Watt MJ, Febbraio MA, Stanley MH, Febbraio Cytokine gene MA, et al. Cytokine gene expression in human skeletal muscle during concentric contraction: evidence that IL-8, like IL-6, is influenced by glycogen availability. Am J Physiol Regul Integr Comp Physiol. 2004;287:322–7.

118. Cho SY, Chung YS, Yoon HK, Roh HT. Impact of Exercise Intensity on Systemic Oxidative Stress, Inflammatory Responses, and Sirtuin Levels in Healthy Male Volunteers. Int J Environ Res Public Health. 2022;19(18):11292.

119. Christiansen T, Bruun JM, Paulsen SK, Ølholm J, Overgaard K, Pedersen SB, et al. Acute exercise increases circulating inflammatory markers in overweight and obese compared with lean subjects. Eur J Appl Physiol. 2013;113(6):1635–42.

120. Cipryan L, Tschakert G, Hofmann P. Acute and Post-Exercise Physiological Responses to High-Intensity Interval Training in Endurance and Sprint Athletes. J Sports Sci Med. 2017;16:219–29.

121. Cipryan L. The effect of fitness level on cardiac autonomic regulation, IL-6, total antioxidant capacity, and muscle damage responses to a single bout of high-intensity interval training. J Sport Health Sci. 2018;7(3):363–71.

122. Collins BEG, Hartmann TE, Marino FE, Skein M. A Comparison of Acute High- and Moderate-Intensity Exercise on Cardio- Metabolic Function and Sleep Among Shift Workers. Journal of Science in Sport and Exercise. 2024;6(1):35–43.

123. Cosio-Lima L, Desai B V, Keck L, Scheeler L, Schuler PB. A comparison of cytokine responses during prolonged cycling in normal and hot environmental conditions. Journal of Sports Medicine. 2011;7–11.

124. Costa RJS, Camões-Costa V, Snipe RMJ, Dixon D, Russo I, Huschtscha Z. The impact of exercise-induced hypohydration on gastrointestinal integrity, function, symptoms, and systemic endotoxin and inflammatory profile. Journal of applied Physiolog [Internet]. 2019;126(5):1281–91. Available from: www.physiology.org/journal/jappl

125. Cox AJ, Pyne DB, Saunders PU, Callister R, Gleeson M. Cytokine responses to treadmill running in healthy and illness-prone athletes. Med Sci Sports Exerc. 2007;39(11):1918–26.

126. Croft L, Bartlett JD, MacLaren DPM, Reilly T, Evans L, Mattey DL, et al. High-intensity interval training attenuates the exercise-induced increase in plasma IL-6 in response to acute exercise. Applied Physiology, Nutrition and Metabolism. 2009;34(6):1098–107.

127. Cullen T, Thomas AW, Webb R, Hughes MG. Interleukin-6 and associated cytokine responses to an acute bout of high intensity interval exercise: the effect of exercise intensity and volume. Appl Physiol Nutr Metab. 2016;(8):803–8.

128. Degerstrøm J, Østerud B. Increased inflammatory response of blood cells to repeated bout of endurance exercise. Med Sci Sports Exerc. 2006;38(7):1297–303.

129. Dorneles GP, Haddad DO, Fagundes VO, Vargas BK, Kloecker A, Romão PRT, et al. High intensity interval exercise decreases IL-8 and enhances the immunomodulatory cytokine interleukin-10 in lean and overweight-obese individuals. Cytokine. 2016;77:1–9.

130. dos Santos T, Lira FS, Antunes BM. Interleukin-15 and creatine kinase response to high-intensity intermittent exercise training. Sport Sci Health. 2020;16(3):479–84.

131. Dufaux B, Order U. Plasma Elastase-al-Antitrypsin, Neopterin, Tumor Necrosis Factor, and Soluble Interleukin-2 Receptor After Prolonged Exercise. Int J Sports Med. 1989;10(6):434–8.

132. Edwards KM, Burns VE, Ring C, Carroll D. Individual differences in the interleukin-6 response to maximal and submaximal exercise tasks. J Sports Sci. 2006;24(8):855–62.

133. Ely MR, Romero SA, Sieck DC, Mangum JE, Luttrell MJ, Halliwill JR. A single dose of histamine-receptor antagonists before downhill running alters markers of muscle damage and delayed-onset muscle soreness. J Appl Physiol. 2017;122(3):631–41.

134. Febbraio MA, Ott P, Nielsen HB, Steensberg A, Keller C, Krustrup P, et al. Hepatosplanchnic clearance of interleukin-6 in humans during exercise. Am J Physiol Endocrinol Metab. 2003;285(2):397–402.

135. Ferguson RA, Dodd MJ, Paley VR. Neuromuscular electrical stimulation via the peroneal nerve is superior to graduated compression socks in reducing perceived muscle soreness following intense intermittent endurance exercise. Eur J Appl Physiol. 2014;114(10):2223–32.

136. Fonseca TR, Mendes TT, Ramos GP, Cabido CET, Morandi RF, Ferraz FO, et al. Aerobic Training Modulates the Increase in Plasma Concentrations of Cytokines in response to a Session of Exercise. J Environ Public Health. 2021;2021:1304139.

137. Gagnon DD, Gagnon SS, Rintamäki H, Törmäkangas T, Puukka K, Herzig KH, et al. The effects of cold exposure on leukocytes, hormones and cytokines during acute exercise in humans. PLoS One. 2014 Oct 22;9(10):e110774.

138. García JJ, Bote E, Hinchado MD, Ortega E. A single session of intense exercise improves the inflammatory response in healthy sedentary women. J Physiol Biochem. 2011 Mar;67(1):87–94.

139. Garneau L, Parsons SA, Smith SR, Mulvihill EE, Sparks LM, Aguer C. Plasma Myokine Concentrations After Acute Exercise in Non-obese and Obese Sedentary Women. Front Physiol. 2020;11:18.

140. Ghafourian M, Ashtary-Larky D, Chinipardaz R, Eskandary N, Mehavaran M. Inflammatory biomarkers’ response to two different intensities of a single bout exercise among soccer players. Iran Red Crescent Med J. 2016;18(2).

141. Gill SK, Hankey J, Wright A, Marczak S, Hemming K, Allerton DM, et al. The Impact of a 24-h Ultra-Marathon on Circulatory Endotoxin and Cytokine Profile. Int J Sports Med. 2015;36(8):688–95.

142. Giraldo E, Garcia JJ, Hinchado MD, Ortega E. Exercise entensity-dependent changes in the nnflammatory response in sedentary women: Role of neuroendocrine parameters in the neutrophil phagocytic process and the pro-/anti-inflammatory cytokine balance. Neuroimmunomodulation. 2009;16(4):237–44.

143. Górecka M, Krzemiński K, Mikulski T, Ziemba AW. ANGPTL4, IL-6 and TNF-α as regulators of lipid metabolism during a marathon run. Sci Rep. 2022;12(1):19940.

144. Goto K, Sumi D, Kojima C, Ishibashi A. Post-exercise serum hepcidin levels were unaffected by hypoxic exposure during prolonged exercise sessions. PLoS One. 2017;12(8):e0183629.

145. Goto K, Kojima C, Kasai N, Sumi D, Hayashi N, Hwang H. Resistance exercise causes greater serum hepcidin elevation than endurance (cycling) exercise. PLoS One. 2020;15(2).

146. Gough L, Penfold RS, Godfrey RJ, Castell L. The immune response to short-duration exercise in trained, eumenorrhoeic women. J Sports Sci. 2015;33(13):1396–402.

147. Gray SR, Clifford M, Lancaster R, Leggate M, Davies M, Nimmo MA. The response of circulating levels of the interleukin-6/interleukin-6 receptor complex to exercise in young men. Cytokine. 2009;47(2):98–102.

148. Gusba JE, Wilson RJ, Robinson DL, Graham TE. Interleukin-6 and its mRNA responses in exercise and recovery: Relationship to muscle glycogen. Scand J Med Sci Sports. 2008;18(1):77–85.

149. Hacker S, Keck J, Reichel T, Eder K, Ringseis R, Krüger K, et al. Biomarkers in Endurance Exercise: Individualized Regulation and Predictive Value. Transl Sports Med. 2023;2023.

150. Hackney AC, Kallman AL, Aǧgön E. Female sex hormones and the recovery from exercise: Menstrual cycle phase affects responses. Biomed Hum Kinet. 2019;11(1):87–9.

151. Harris N, Kilding A, Sethi S, Merien F, Gottschall J. A comparison of the acute physiological responses to BODYPUMP^TM^ versus iso-caloric and iso-time steady state cycling. J Sci Med Sport. 2018;21(10):1085–9.

152. He Z, Tian Y, Valenzuela PL, Huang C, Zhao J, Hong P, et al. Myokine Response to High-Intensity Interval vs. Resistance Exercise: An Individual Approach. Front Physiol. 2018;9:1735.

153. He Z, Tian Y, Valenzuela PL, Huang C, Zhao J, Hong P, et al. Myokine Response to High-Intensity Interval vs. Resistance Exercise: An Individual Approach. Front Physiol. 2018;9.

154. He Z, Tian Y, Valenzuela PL, Huang C, Zhao J, Hong P, et al. Myokine/adipokine response to “aerobic” exercise: Is it just a matter of exercise load? Front Physiol. 2019;10:691.

155. Hojman P, Brolin C, Nørgaard-Christensen N, Dethlefsen C, Lauenborg B, Olsen CK, et al. IL‐6 release from muscles during exercise is stimulated by lactate‐dependent protease activity. American Journal of Physiology-Endocrinology and Metabolism. 2019;316(5):E940–7.

156. Islam H, Townsend LK, McKie GL, Medeiros PJ, Gurd BJ, Hazell TJ. Potential involvement of lactate and interleukin-6 in the appetite-regulatory hormonal response to an acute exercise bout. J Appl Physiol. 2017;123(3):614–23.

157. Islam H, Tsai SH, Figueiredo C, Jackson GS, Marcotte-Chénard A, Bosak J, et al. Direct assessment of leukocyte signalling and cytokine secretion reveals exercise intensity-dependent reductions in anti-inflammatory cytokine action Key points. J Physiol. 2024;602:2717–36.

158. Jimenez C, Melin B, Savourey G, Launay JC, Alonso A, Mathieu J. Effects of passive hyperthermia versus exercise-induced hyperthermia on immune responses: Hormonal implications. Eur Cytokine Netw. 2007 Sep;18(3):154–61.

159. Joisten N, Kummerhoff F, Koliamitra C, Schenk A, Walzik D, Hardt L, et al. Current state of knowledge and results from a randomized cross-over study comparing acute effects of endurance and resistance training. Exerc Immunol Rev. 2020;26:24–42.

160. Jürimäe J, Rämson R, Mäestu J, Jürimäe T, Arciero PJ, Braun WA, et al. Interactions between adipose, bone, and muscle tissue markers during acute negative energy balance in male rowers. J Sports Med Phys Fitness. 2011;51(2):347–54.

161. Kakanis MW, Peake J, Brenu EW, Simmonds M, Gray B, Marshall-Gradisnik SM. T helper cell cytokine profiles after endurance exercise. Journal of Interferon and Cytokine Research. 2014;34(9):699–706.

162. Kastelein TE, Duffield R, Marino FE. Acute immune-inflammatory responses to a single bout of aerobic exercise in smokers; The effect of smoking history and status. Front Immunol. 2015;6(DEC).

163. Kastelein TE, Donges CE, Mendham AE, Duffield R. The Acute Exercise-Induced Inflammatory Response: A Comparison of Young-Adult Smokers and Nonsmokers. Res Q Exerc Sport. 2017 Jan 2;88(1):15–25.

164. Kim HK, Konishi M, Takahashi M, Tabata H, Endo N, Numao S, et al. Effects of acute endurance exercise performed in the morning and evening on inflammatory cytokine and metabolic hormone responses. PLoS One. 2015;10(9):e0137567.

165. Kon M, Tanimura Y. Responses of complement C1q/tumor necrosis factor-related proteins to acute aerobic exercise. Cytokine. 2023;161:156083.

166. Krzemiński K, Buraczewska M, Miśkiewicz Z, Dąbrowski J, Steczkowska M, Kozacz A, et al. Effect of ultra-endurance exercise on left ventricular performance and plasma cytokines in healthy trained men. Biol Sport. 2016;33(1):63–9.

167. Kuhne LA, Ksiezarczyk AM, Braumann KM, Reer R, Jacobs T, Röder B, et al. Cardiovascular exercise, learning, memory, and cytokines: Results of a ten-week randomized controlled training study in young adults. Biol Psychol. 2023;176:108466.

168. Landers-Ramos RQ, Dondero K, Nelson C, Ranadive SM, Prior SJ, Addison O. Muscle thickness and inflammation during a 50km ultramarathon in recreational runners. PLoS One. 2022;17(9):e0273510.

169. Larsen B, Cox AJ, Quinn K, Fisher R, Minahan C. in Women during Exercise in the Heat: A Spotlight on Oral Contraception. J Sports Sci Med. 2018;17:229–36.

170. Lavoy ECP, Nieman DC, Henson DA, Shanely RA, Knab AM, Cialdella-Kam L, et al. Latent cytomegalovirus infection and innate immune function following a 75 km cycling time trial. Eur J Appl Physiol. 2013;113(10):2629–35.

171. Leggate M, Nowell MA, Jones SA, Nimmo MA. The response of interleukin-6 and soluble interleukin-6 receptor isoforms following intermittent high intensity and continuous moderate intensity cycling. Cell Stress Chaperones. 2010 Nov;15(6):827–33.

172. Li TL, Gleeson M. The Effect of Single and Repeated Bouts of Prolonged Cycling on Leukocyte Redistribution, Neutrophil Degranulation, IL-6, and Plasma Stress Hormone Responses. Int J Sport Nutr Exerc Metab. 2004;14(5):501–16.

173. Lira FS, dos Santos T, Caldeira RS, Inoue DS, Panissa VLG, Cabral-Santos C, et al. Short-term high- and moderate-intensity training modifies inflammatory and metabolic factors in response to acute exercise. Front Physiol. 2017;8:856.

174. Lobo LF, Morais MG de, Marcucci-Barbosa LS, Martins-Junior F de AD, Avelar LM, Vieira ELM, et al. A Single Bout of Fatiguing Aerobic Exercise Induces Similar Pronounced Immunological Responses in Both Sexes. Front Physiol. 2022;13:833580.

175. MacNeil LG, Tarnopolsky MA, Crane JD. Acute, Exercise-Induced Alterations in Cytokines and Chemokines in the Blood Distinguish Physically Active and Sedentary Aging. Journals of Gerontology - Series A Biological Sciences and Medical Sciences. 2021;76(5):811–8.

176. Maharaj A, Slusher AL, Zourdos MC, Whitehurst M, Fico BG, Huang CJ. Association of calprotectin with leukocyte chemotactic and inflammatory mediators following acute aerobic exercise. Applied Physiology, Nutrition and Metabolism. 2015;41(1):83–7.

177. Marcucci-Barbosa LS, Martins-Junior F, Lobo LF, Morais MG, Moreira JM, Vieira ELM, et al. 10 km running race induces an elevation in the plasma myokine level of nonprofessional runners. Sport Sci Health. 2020;16(2):313–21.

178. Marklund P, Mattsson CM, Wåhlin-Larsson B, Ponsot E, Lindvall B, Lindvall L, et al. Extensive inflammatory cell infiltration in human skeletal muscle in response to an ultraendurance exercise bout in experienced athletes. J Appl Physiol. 2013;114:66–72.

179. Markus I, Constantini K, Goldstein N, Amedi R, Bornstein Y, Stolkovsky Y, et al. Age Differences in Recovery Rate Following an Aerobic-Based Exercise Protocol Inducing Muscle Damage Among Amateur, Male Athletes. Front Physiol. 2022;13:916924.

180. McCormick R, Moretti D, McKay AKA, Laarakkers CM, Vanswelm R, Trinder D, et al. The Impact of Morning versus Afternoon Exercise on Iron Absorption in Athletes. Med Sci Sports Exerc. 2019;51(10):2147–55.

181. McKay AKA, McCormick R, Tee N, Peeling P. Exercise and heat stress: Inflammation and the iron regulatory response. Int J Sport Nutr Exerc Metab. 2021;31(6):460–5.

182. McKenzie MJ, Goldfarb A, Garten RS, Vervaecke L. Oxidative stress and inflammation response following aerobic exercise: Role of ethnicity. Int J Sports Med. 2014;35(10):822–7.

183. Mendham AE, Duffield R, Marino F, Coutts AJ. Differences in post-exercise inflammatory and glucose regulatory response between sedentary Indigenous Australian and Caucasian men completing a single bout of cycling. American Journal of Human Biology. 2014;26(2):208–14.

184. Mills DE, Johnson MA, Mcphilimey MJ, Williams NC, Gonzalez JT, Barnett YA, et al. The effects of inspiratory muscle training on plasma interleukin-6 concentration during cycling exercise and a volitional mimic of the exercise hyperpnea. J Appl Physiol. 2013;115:1163–72.

185. Minuzzi LG, Lira FS, de Poli RAB, Fialho Lopes VH, Zagatto AM, Suzuki K, et al. High-intensity intermittent exercise induces a potential anti-inflammatory response in healthy women across the menstrual cycle. Cytokine. 2022;154:155872.

186. Moldoveanu AI, Shephard RJ, Shek PN. Exercise elevates plasma levels but not gene expression of IL-1, IL-6, and TNF-in blood mononuclear cells. J Appl Physiol. 2000;89(4):1499–504.

187. Neidhart M, Müller-Ladner U, Frey W, Bosserhoff AK, Colombani PC, Frey-Rindova P, et al. Increased serum levels of non-collagenous matrix proteins (cartilage oligomeric matrix protein and melanoma inhibitory activity) in marathon runners. Osteoarthritis Cartilage. 2000;8(3):222–9.

188. Nelson AB, Chow LS, Stagg DB, Gillingham JR, Evans MD, Pan M, et al. Acute aerobic exercise reveals that FAHFAs distinguish the metabolomes of overweight and normal-weight runners. JCI Insight. 2022;7(7):e158037.

189. Newlin MK, Williams S, Mcnamara T, Tjalsma H, Swinkels DW, Haymes EM. The Effects of Acute Exercise Bouts on Hepcidin in Women. Int J Sport Nutr Exerc Metab. 2012;22:79–88.

190. Nieman DC, Konrad M, Henson DA, Kennerly K, Shanely RA, Wallner-Liebmann SJ. Variance in the acute inflammatory response to prolonged cycling is linked to exercise intensity. Journal of Interferon and Cytokine Research. 2012;32(1):12–7.

191. Nieman DC, Shanely RA, Luo B, Meaney MP, Dew DA, Pappan KL. Metabolomics approach to assessing plasma 13-and 9-hydroxy-octadecadienoic acid and linoleic acid metabolite responses to 75-km cycling. Am J Physiol Regul Integr Comp Physiol. 2014;307:68–74.

192. Nieman DC, Zwetsloot KA, Meaney MP, Lomiwes DD, Hurst SM, Hurst RD. Post-Exercise Skeletal Muscle Glycogen Related to Plasma Cytokines and Muscle IL-6 Protein Content, but not Muscle Cytokine mRNA Expression. Front Nutr. 2015;2(27).

193. Nieman DC, Meaney MP, John CS, Knagge KJ, Chen H. 9- and 13-Hydroxy-octadecadienoic acids (9 + 13 HODE) are inversely related to granulocyte colony stimulating factor and IL-6 in runners after 2 h running. Brain Behav Immun. 2016;56:246–52.

194. Nieman DC, Zwetsloot KA, Lomiwes DD, Meaney MP, Hurst RD. Muscle glycogen depletion following 75-km of cycling is not linked to increased muscle IL-6, IL-8, and MCP-1 mRNA expression and protein content. Front Physiol. 2016;7(SEP):431.

195. Niess AM, Fehrenbach E, Strobel G, Roecker K, Schneider EM, Buergler J, et al. Evaluation of stress responses to interval training at low and moderate altitudes. Med Sci Sports Exerc. 2003;35(2):263–9.

196. Niess AM, Fehrenbach E, Lehmann R, Opavsky L, Jesse M, Northoff H, et al. Impact of elevated ambient temperatures on the acute immune response to intensive endurance exercise. Eur J Appl Physiol. 2003;89(3–4):344–51.

197. Osborne JO, Stewart IB, Beagley KW, Minett GM. The effect of cycling in the heat on gastrointestinal-induced damage and neuromuscular fatigue. Eur J Appl Physiol. 2019;119(8):1829–40.

198. Ostrowski K, Hermann C, Bangash A, Schjerling P, Nielsen JN, Pedersen BK. A trauma-like elevation of plasma cytokines in humans in response to treadmill running. J Physiol. 1998;513(3):889–94.

199. Pasqua LA, Damasceno M V., Cruz R, Matsuda M, Martins MAG, Marquezini M V., et al. Exercising in the urban center: Inflammatory and cardiovascular effects of prolonged exercise under air pollution. Chemosphere. 2020;254.

200. Pawłowska M, Mila-Kierzenkowska C, Boraczyński T, Boraczyński M, Szewczyk-Golec K, Sutkowy P, et al. The Influence of Ambient Temperature Changes on the Indicators of Inflammation and Oxidative Damage in Blood after Submaximal Exercise. Antioxidants. 2022;11(12):2445.

201. Peake JM, Suzuki K, Wilson G, Hordern M, Nosaka K, MacKinnon L, et al. Exercise-induced muscle damage, plasma cytokines, and markers of neutrophil activation. Med Sci Sports Exerc. 2005;37(5):737–45.

202. Peake JM, Suzuki K, Hordern M, Wilson G, Nosaka K, Coombes JS. Plasma cytokine changes in relation to exercise intensity and muscle damage. Eur J Appl Physiol. 2005;95(5–6):514–21.

203. Peake J, Peiffer JJ, Abbiss CR, Nosaka K, Okutsu M, Laursen PB, et al. Body temperature and its effect on leukocyte mobilization, cytokines and markers of neutrophil activation during and after exercise. Eur J Appl Physiol. 2008;102(4):391–401.

204. Peake JM, Tan SJ, Markworth JF, Broadbent JA, Skinner TL, Cameron-Smith D. Metabolic and hormonal responses to isoenergetic high-intensity interval exercise and continuous moderate-intensity exercise. Am J Physiol Endocrinol Metab. 2014;307(7):E539–52.

205. Peeling P, Dawson B, Goodman C, Landers G, Wiegerinck ET, Swinkels DW, et al. Effects of Exercise on Hepcidin Response and Iron Metabolism During Recovery. Int J Sport Nutr Exerc Metab. 2009;19:583–97.

206. Peeling P, Dawson B, Goodman C, Landers G, Wiegerinck ET, Swinkels DW, et al. Cumulative effects of consecutive running sessions on hemolysis, inflammation and hepcidin activity. Eur J Appl Physiol. 2009;106(1):51–9.

207. Peeling P, Sim M, Badenhorst CE, Dawson B, Govus AD, Abbiss CR, et al. Iron status and the acute post-exercise hepcidin response in athletes. PLoS One. 2014;9(3):e93002.

208. Peeling P, McKay AKA, Pyne DB, Guelfi KJ, McCormick RH, Laarakkers CM, et al. Factors influencing the post-exercise hepcidin-25 response in elite athletes. Eur J Appl Physiol. 2017;117(6):1233–9.

209. Pilat C, Krüger K, Frech T, Mooren FC. Exercise-induced cytokine changes in antigen stimulated whole-blood cultures compared to serum. J Immunol Methods. 2017 Jan 1;440:58–66.

210. Panizo González N, Reque Santivañez JE, Hernando Fuster B, Collado Boira EJ, Martinez-Navarro I, Chiva Bartoll Ó, et al. Quick Recovery of Renal Alterations and Inflammatory Activation after a Marathon. Kidney Diseases. 2019;5(4):259–65.

211. Pistilli EE, Nieman DC, Henson DA, Kaminsky DE, Utter AC, Vinci DM, et al. Influence of Age on Immune Changes in Runners After a Marathon. Journal of Aging and Physical Fitness. 2002;10(4):432–42.

212. Pokora I, Kempa K, Chrapusta SJ, Langfort J. Effects of downhill and uphill exercises of equivalent submaximal intensities on selected blood cytokine levels and blood creatine kinase activity. Biol Sport. 2014;31(3):173–8.

213. Pournot H, Bieuzen F, Louis J, Fillard JR, Barbiche E, Hausswirth C. Time-Course of changes in inflammatory response after whole-body cryotherapy multi exposures following severe exercise. PLoS One. 2011;6(7).

214. Pozzolo BA, da Fonseca VF, Guedes AA, de Oliveira GL, Dietrich D, Lima EM, et al. Acute effect of aerobic exercise of different intensities in cytokines of university students. Revista Brasileira de Medicina do Esporte. 2020;26(6):493–7.

215. Proschinger S, Schenk · Alexander, Weßels I, Donath L, Rappelt · Ludwig, Metcalfe AJ, et al. Intensity-and time-matched acute interval and continuous endurance exercise similarly induce an anti-inflammatory environment in recreationally active runners: focus on PD-1 expression in T regs and the IL-6/IL-10 axis Abbreviations BMI Body mass index EDTA Ethylenediaminetetraacetic acid. Eur J Appl Physiol. 2023;123(3):2575–84.

216. Rämson R, Jürimäe J, Jürimäe T, Mäestu J. The influence of increased training volume on cytokines and ghrelin concentration in college level male rowers. Eur J Appl Physiol. 2008;104(5):839–46.

217. Reichel T, Boßlau TK, Palmowski J, Eder K, Ringseis R, Mooren FC, et al. Reliability and suitability of physiological exercise response and recovery markers. Sci Rep. 2020;10(1):1–11.

218. Rinnov A, Yfanti C, Nielsen S, Åkerström TCA, Peijs L, Zankari A, et al. Endurance training enhances skeletal muscle interleukin-15 in human male subjects. Endocrine. 2014;45(2):271–8.

219. Robson-Ansley P, Barwood M, Canavan J, Hack S, Eglin C, Davey S, et al. The effect of repeated endurance exercise on IL-6 and sIL-6R and their relationship with sensations of fatigue at rest. Cytokine. 2009;45(2):111–6.

220. Rohde T, Maclean DA, Hartkopp A, Pedersen BK, Pedersen BK. The immune system and serum glutamine during a triathlon. Eur J Appl Physiol. 1996;74:428–34.

221. Ronsen O, Lea T, Bahr R, Klarlund Pedersen B, Klar-lund Pedersen B, Ronsen O. Enhanced plasma IL-6 and IL-1ra responses to repeated vs. single bouts of prolonged cycling in elite athletes. J Appl Physiol. 2002;92:2547–53.

222. Said M, Feki Y, Aouni Z, Machghoul S, Hamza M, Amri M. Effects of sustained intensive physical activities on immune cells circulating and pro-inflammatory cytokines production in trained and untrained humans. Sci Sports. 2009;24(5):229–37.

223. Sawai A, Shida T, Hoshikawa Y, Hatanaka S, Ueda M, Kato Y, et al. Effect of acute moderate-intensity cycling on cfDNA levels considering menstrual cycle phases. Front Sports Act Living. 2024;6:1322295.

224. Scharhag J, Meyer T, Gabriel HHW, Schlick B, Faude O, Kindermann W. Does prolonged cycling of moderate intensity affect immune cell function? Br J Sports Med. 2005;39(3):171–7.

225. Scott JPR, Sale C, Greeves JP, Casey A, Dutton J, Fraser WD. Effect of exercise intensity on the cytokine response to an acute bout of running. Med Sci Sports Exerc. 2011;43(12):2297–306.

226. Scott JPR, Sale C, Greeves JP, Casey A, Dutton J, Fraser WD. Cytokine response to acute running in recreationally-active and endurance-trained men. Eur J Appl Physiol. 2013;113(7):1871–82.

227. Shojaei EA, Jafari A, Farajov A. Effect of acute moderate aerobic cycling on systemic inflammatory responses in young untrained men. Sci Sports. 2011;26(5):298–302.

228. Shojaei EA, Farajov A, Jafari A. Effect of moderate aerobic cycling on some systemic inflammatory markers in healthy active collegiate men. Int J Gen Med. 2011;4:79–84.

229. Silva Acevedo PA, Nóbrega C, Darck Carola Correia Lima J, Marques de Matos-Neto E, Carlos Amadio A, Cerqueira Leite Seelaender M, et al. Immediate effects of a real moderate interval-running training session on inflammatory profile. Cytokine. 2020;133.

230. Sim M, Dawson B, Landers G, Swinkels DW, Tjalsma H, Trinder D, et al. Effect of Exercise Modality and Intensity on Postexercise Interleukin-6 and Hepcidin Levels. Int J Sport Nutr Exerc Metab. 2013;23:178–86.

231. Sim M, Dawson B, Landers G, Swinkels DW, Tjalsma H, Yeap BB, et al. Oral contraception does not alter typical post-exercise interleukin-6 and hepcidin levels in females. J Sci Med Sport. 2015;18(1):8–12.

232. Simpson RJ, Wilson MR, Black JR, Ross JA, Whyte GP, Guy K, et al. Immune Alterations, Lipid Peroxidation, and Muscle Damage Following a Hill Race. J Appl Physiol. 2005;30(2):196–211.

233. Siqueira IR, Basso CG, Pizzolato LS, Cechinel LR, Bertoldi K, Dalpiaz M, et al. Correlation Between Inflammatory and Epigenetic Marks With Aerobic Performance in 10-km Runners. Res Q Exerc Sport. 2022;93(1):77–86.

234. Smith LL, McKune AJ, Semple SJ, Sibanda E, Steel H, Anderson R. Changes in serum cytokines after repeated bouts of downhill running. Applied Physiology, Nutrition and Metabolism. 2007;32(2):233–40.

235. Snipe RMJ, Khoo A, Kitic CM, Gibson PR, Costa RJS. The Impact of Mild Heat Stress during Prolonged Running on Gastrointestinal Integrity, Gastrointestinal Symptoms, Systemic Endotoxin and Cytokine Profiles. Int J Sports Med. 2018;39(4):255–63.

236. Spanoudaki SS, St V, Spanoudaki S, Maridaki M, Tsironi M, Baltopoulos G, et al. Immune responses during and after exercise of constant and alternating intensity above the lactate threshold. J SPORTS MED PHYS FITNESS. 2010;50:363–70.

237. Starkie RL, Angus DJ, Rolland J, Hargreaves M, Febbraio MA. Effect of prolonged, submaximal exercise and carbohydrate ingestion on monocyte intracellular cytokine production in humans. Journal of Physiology. 2000;528(3):647–55.

238. Starkie RL, Rolland J, Angus DJ, Anderson MJ, Febbraio MA, Ander MJ. Circulating monocytes are not the source of elevations in plasma IL-6 and TNF-α levels after prolonged running. Am J Physiol Cell Physiol. 2001;280:769–74.

239. Starkie RL, Hargreaves M, Rolland J, Febbraio MA. Heat stress, cytokines, and the immune response to exercise. Brain Behav Immun. 2005;19(5):404–12.

240. Starzak DE, Semple S, Smith L, Mckune AJ. Differing cytokine responses by ethnic groups to a bout of exercise-induced muscle damage: a preliminary report. 2015;6(56):665–77.

241. Steensberg A, van Hall G, Osada T, Sacchetti M, Saltin B, Pedersen BK. Production of interleukin‐6 in contracting human skeletal muscles can account for the exercise‐induced increase in plasma interleukin‐6. J Physiol. 2000 Nov;529(1):237–42.

242. Sugama K, Suzuki K, Yoshitani K, Shiraishi K, Kometani T. IL-17, neutrophil activation and muscle damage following endurance exercise. Exerc Immunol Rev. 2012;18.

243. Sugama K, Suzuki K, Yoshitani K, Shiraishi K, Kometani T. Urinary excretion of cytokines versus their plasma levels after endurance exercise. Exerc Immunol Rev. 2013;19.

244. Sumi D, Kojima C, Goto K. Impact of endurance exercise in hypoxia on muscle damage, inflammatory and performance responses. J Strength Cond Res. 2018;32(4):1053–62.

245. Sureda A, Mestre-Alfaro A, Banquells M, Riera J, Drobnic F, Camps J, et al. Exercise in a hot environment influences plasma anti-inflammatory and antioxidant status in well-trained athletes. J Therm Biol. 2015;47:91–8.

246. Suzuki K, Totsuka M, Nakaji S, Yamada M, Kudoh S, Liu Q, et al. Endurance exercise causes interaction among stress hormones, cytokines, neutrophil dynamics, and muscle damage. J Appl Physiol. 1999;87(4):1360–7.

247. Svendsen IS, Hem E, Gleeson M. Effect of acute exercise and hypoxia on markers of systemic and mucosal immunity. Eur J Appl Physiol. 2016;116(6):1219–29.

248. Tartibian B, Azadpoor N, Abbasi A. Effects of two different type of treadmill running on human blood leukocyte populations and inflammatory indices in young untrained men. Journal of sports medicine and physical fitness. 2009;49(2):214.

249. Timmons BW, Hamadeh MJ, Devries MC, Tarnopolsky MA. Inﬂuence of gender, menstrual phase, and oral contraceptive use on immunological changes in response to prolonged cycling. J Appl Physiol. 2005;99(3):979–85.

250. Timmons BW, Hamadeh MJ, Tarnopolsky MA. Two methods for determining plasma IL-6 in humans at rest and following exercise. Eur J Appl Physiol. 2009;105(1):13–8.

251. Tsuchiya Y, Goto K. Myokine secretion following moderate-intensity endurance exercise under different environmental temperatures. Cytokine. 2021;144:155553.

252. Ullum H, Martin Haahr P, Diamant M, Palmo J, Halkjaer-Kristensen J, Klarlund Pedersen B. Bicycle exercise enhances plasma IL-6 but does not change IL-la, IL-I& IL-6, or TNF-a pre-mRNA in BMNC. J Appl Physiol. 1994;1(77):93–7.

253. Vaisberg M, Bachi ALL, Latrilha C, Dioguardi GS, Bydlowski SP, Maranhão RC. Lipid transfer to HDL is higher in marathon runners than in sedentary subjects, but is acutely inhibited during the run. Lipids. 2012;47(7):679–86.

254. van De Vyver M, Myburgh KH. Cytokine and satellite cell responses to muscle damage: Interpretation and possible confounding factors in human studies. J Muscle Res Cell Motil. 2012;33(3–4):177–85.

255. van de Vyver M, Myburgh KH. Variable inflammation and intramuscular STAT3 phosphorylation and myeloperoxidase levels after downhill running. Scand J Med Sci Sports. 2014;24(5):e360–71.

256. van de Vyver M, Engelbrecht L, Smith C, Myburgh KH. Neutrophil and monocyte responses to downhill running: Intracellular contents of MPO, IL-6, IL-10, pstat3, and SOCS3. Scand J Med Sci Sports. 2016;26(6):638–47.

257. Venhorst A, Micklewright DP, Noakes TD. The Psychophysiological Regulation of Pacing Behaviour and Performance Fatigability During Long-Distance Running with Locomotor Muscle Fatigue and Exercise-Induced Muscle Damage in Highly Trained Runners. Sports Med Open. 2018;4(1).

258. Wadley AJ, Chen YW, Lip GYH, Fisher JP, Aldred S. Low volume-high intensity interval exercise elicits antioxidant and anti-inflammatory effects in humans. J Sports Sci. 2016;34(1):1–9.

259. Wadley J, Keane Tom Cullen Lynsey James G, Vautrinot Matthew J, Bethan Hussey D, Hunter ac Sarabjit Mastana J, Holliday Steen A, et al. Characterisation of extracellular redox enzyme concentrations in response to exercise in humans. J Appl Physiol. 2019;127(3):858–66.

260. Wahl P, Hein M, Achtzehn S, Bloch W, Mester J. Acute effects of superimposed electromyostimulation during cycling on myokines and markers of muscle damage. J Musculoskelet Neuronal Interact. 2015;(1):53–9.

261. Wallberg L, Mikael Mattsson C, Enqvist JK, Ekblom B. Plasma IL-6 concentration during ultra-endurance exercise. Eur J Appl Physiol. 2011;111(6):1081–8.

262. Walshe I, Robson-Ansley P, St Clair Gibson A, Lawrence C, Thompson KG, Ansley L. The reliability of the IL-6, sIL-6R and sgp130 response to a preloaded time trial. Eur J Appl Physiol. 2010;110(3):619–25.

263. Wang Y, Xu T, Zhao H, Gu C, Li Z. Effect of taurine in muscle damage markers and inflammatory cytokines in running exercise. Front Physiol. 2022;13:1008060.

264. Woo J, Min JH, Lee YH, Roh HT. Effects of hyperbaric oxygen therapy on inflammation, oxidative/antioxidant balance, and muscle damage after acute exercise in normobaric, normoxic and hypobaric, hypoxic environments: A pilot study. Int J Environ Res Public Health. 2020;17(20):1–10.

265. Zaldivar F, Wang-Rodriguez J, Nemet D, Schwindt C, Galassetti P, Mills PJ, et al. Constitutive pro-and anti-inflammatory cytokine and growth factor response to exercise in leukocytes. J Appl Physiol. 2006;100(4):1124–33.

266. Zheng H, Badenhorst CE, Lei TH, Liao YH, Muhamed AMC, Fujii N, et al. Menstrual phase and ambient temperature do not influence iron regulation in the acute exercise period. Am J Physiol Regul Integr Comp Physiol. 2021;320(6):780–90.
